# Supplementary material for: Intervention planning for the REDUCE maintenance intervention: a digital intervention to reduce reulceration risk among patients with a history of diabetic foot ulcers
Source: BMJ Open. 2018 May 18;8(5):e019865. doi: 10.1136/bmjopen-2017-019865 (PMC5961606; doi:10.1136/bmjopen-2017-019865)
Supplement: Supplementary file 1 [file bmjopen-2017-019865supp001.pdf]

## APPENDIX 1: INTERVIEW SCHEDULE AND PROMPT CARDS

### Interview schedule

Section 1: Context

Q1. Can you tell me a bit about what it has been like for you to have a foot ulcer?
Q2. Can you tell me about anything that you do to look after your feet currently?

Section 2: Acceptability and feasibility of the maintenance intervention

*[Provide explanation of initiation phase and maintenance intervention]*

1. Content of website/booklet

Card 1 (foot checking):

Q3. What do you think about these ideas? What do you like about these ideas? What do you dislike about these ideas?
Q4. The foot checker will ask you to check your feet every day for foot damage. What things might make it difficult for you to do this? Do you have any concerns about checking your feet every day?

Card 2 (help-seeking):

Q5. What do you think about these ideas? What do you like about these ideas? What do you dislike about these ideas?
Q6. The website may ask you to contact your diabetes team if you are concerned about any foot damage you have. What things might make it difficult for you to do this? Do you have any concerns about doing this?

Card 3 (physical activity):

Q7. What do you think about these ideas? What do you like about these ideas? What do you dislike about these ideas?
Q8. What things might make it difficult for you to get more active? Do you have any concerns about getting more active?

Card 4 (dealing with feelings):

Q9. What do you think about these ideas? What do you like about these ideas? What do you dislike about these ideas? Is there anything you do at the moment that helps you when you feel stressed / low?

Additional content:

Q10. What other things could we do to help you to look after your feet?

2. Delivery formats

Q11. What are your thoughts about the long-term support for maintaining habits being provided in **a booklet**? What do you like about this idea? What do you dislike about this idea?

Q12. What are your thoughts about the long-term support for maintaining habits being provided through **a website**? What do you like about this idea? What do you dislike about this idea?

Q13. If we were to deliver the programme through a website, what do you think about the idea of using **a computer or tablet**, such as an iPad, to access the website?

Q14. What do you think about the idea of using **a mobile phone** to access the website?

Card 5 (optional health professional support):

Q15. What do you think about this idea? What do you like about this idea? What do you dislike about this idea?

Q16. [If optional support would be helpful] What would you like to talk to the health professional about? Why would this be helpful?

Q17. You could contact the health professional in person, over the phone, and by email. Which one of these options would you prefer? Why?

**Prompt Cards**

Card 1 – Check your feet regularly

*Why is this important?*

It can be difficult to know when you might be developing a foot ulcer because some of the initial signs can be very small and hard to spot. It is important to examine your feet regularly so you are better able to spot any changes in your feet.

The website or booklet will:

- Ask you to check your feet every day
- Allow you to make a note of any changes in your feet

Set up regular reminders to check your feet which the foot checker can send to you by email or text messages to your mobile phone

Card 2 – What to do if you spot any foot damage

*Why is this important?*

It is important to report any changes in your feet to your diabetes team as soon as possible. This will allow them to check your feet and see if you need any treatment. The quicker your feet are treated, the more likely that any damage to your feet will heal.

The website or booklet will:

- Give you personalised advice on what to do if you spot any changes in your feet
- Advise you when you may need to contact your diabetes team

Card 3 – Getting active

*Why is this important?*

When you don't have a foot ulcer, it is safe and important to be mobile and active. This is because being active will improve your circulation and blood sugar, and reduce your chances of getting another ulcer. It is important that you pace your activity – little and often is best. Irregular activity (doing nothing and then doing too much) can be harmful because it increases the chances of injuring your feet.

The website or booklet will:

- Help you to slowly increase physical activity, such as using an exercise bike, seated exercise, walking, or any other things you like to do to be active.
- Set weekly physical activity goals, for example, going for a walk once or a few times a week.
- Ask you to enter in information about how you got on with your goals each week and provide you with personalised advice based on your progress.

A free step counter (or pedometer) that clips onto your belt and counts how many steps you take. You can use this to set yourself daily or weekly step goals if you would like to.

Card 4 – Dealing with your feelings when you get another ulcer

*Why is this important?*

We know that people who have had an ulcer can feel frustrated and cross if, and when, they get another one. Some people can feel down or stressed at this time and it can be difficult to look after yourself when you are feeling this way. Dealing with your feelings can make you feel better and will make sure you put you and your health first.

The website or booklet will:

- Teach you techniques that have been shown to help improve mood and reduce stress.
- Many people have found these techniques to be helpful.

Card 5 - Optional health professional support:

The website or booklet will also:

- Give people the option to contact a diabetes trained health professional if they wanted to.
- This contact could be in person, over the phone, or by email.
