# Supplementary material for: Intervention planning for the REDUCE maintenance intervention: a digital intervention to reduce reulceration risk among patients with a history of diabetic foot ulcers
Source: BMJ Open. 2018 May 18;8(5):e019865. doi: 10.1136/bmjopen-2017-019865 (PMC5961606; doi:10.1136/bmjopen-2017-019865)
Supplement: Supplementary file 2 [file bmjopen-2017-019865supp002.pdf]

APPENDIX 2: BEHAVIOURAL ANALYSIS OF REDUCE MAINTENANCE INTERVENTION USING THE BEHAVIOUR CHANGE WHEEL (BCW) AND BEHAVIOUR CHANGE TECHNIQUES TAXONOMY (BCTv1)

Key: DFU = Diabetic Foot Ulcer; EO = Barrier emerged from expert opinion; LR = Barrier emerged from literature review; QR = Barrier emerged from qualitative primary research; N/A = theoretical mapping not applicable; * = intervention components and BCTs identified through examination of the BCTv1 and BCW to check for additional intervention functions, target constructs, or behaviour change techniques.

**Barriers/*facilitator* to target behaviour**	**Intervention components**	**Target construct (BCW)**	**Intervention function (BCW)**	**Behaviour Change Technique (using BCTv1)**
**Target behaviour: Engaging in regular foot checking**				
Belief that foot checking will <u>do little</u> to delay getting a DFU [EO; LR]	• Provide a rationale for the necessity of regular foot checking, including evidence that it is effective for delaying DFUs	Psychological capability; Reflective motivation	Education; Persuasion	5.1 Information about health consequences<br>5.6 Information about emotional consequences
	• Provide patient stories demonstrating how regular foot checking helped other patients to take control of their DFUs	Psychological capability; Reflective motivation; Social opportunity	Education; Persuasion; Modelling	5.1 Information about health consequences<br>6.2 Social comparison<br>6.3 Information about others' approval<br>9.1 Credible source
Lack of knowledge regarding DFUs and foot checking procedures/ Lack of confidence in ability to check feet [LR, QR]	• Provide information and pictures on what DFUs look like, what signs of DFUs to look out for, and how to check their feet	Physical capability; Psychological capability	Education; Training	4.1 Instructions on how to perform the behaviour<br>5.1 Information about health consequences
	• Provide an online and printable foot health checklist so patients can spot changes in their foot health<br>• Allow patients to record any changes in their foot health	Psychological capability; Physical opportunity	Training; Environmental structuring	4.1 Instructions on how to perform the behaviour<br>12.5 Adding objects to the environment
	• Provide patient stories demonstrating how easy it was for other patients to engage in regular foot checking	Reflective motivation; Social opportunity	Persuasion; Modelling	6.2 Social comparison<br>6.3 Information about others' approval<br>9.1 Credible source
Forgetting to check your feet [EO, QR]<br><br>*Reminders to check your feet [EO,QR]*	• Allow patients to set up regular reminders to check your feet daily by email or text messages and decide on the frequency of these reminders	Physical opportunity	Environmental structuring	7.1 Prompts/cues

**Barriers/*facilitator* to target behaviour**	**Intervention components**	**Target construct (BCW)**	**Intervention function (BCW)**	**Behaviour Change Technique (using BCTv1)**
Difficulties engaging in foot checking in the long-term [LR, QR]<br><br>*Integrating foot checking into your routine [QR]*	• Allow patients to set their own daily foot checking goals<br>• Encourage patients to make a foot checking action plan	Reflective motivation	Enablement	1.1 Goal setting (behaviour)<br>1.4 Action planning<br>1.5 Review behaviour goal(s)
	• Advise patients to regularly practice foot checking in the same context (e.g. after showering, when putting socks on)	Psychological capability; Automatic motivation	Training; Enablement	8.1 Behavioural practice/rehearsal<br>8.3 Habit formation
Physical limitations, deformities and barriers (e.g. limited mobility, foot deformities, poor eyesight) [LR, QR]<br><br>*Using a mirror [QR]; Getting someone to check your feet for you [QR]*	• Provide guidance on how to check your feet if you have physical limitations, including using a mirror to check the bottom of your feet and asking someone else to check for you<br>• Provide information on the signs of DFUs and foot checking procedures for significant others who are helping with foot checking	Physical capability; Psychological capability; Social opportunity	Training; Education; Enablement	3.2 Social support (practical)<br>4.1 Instructions on how to perform the behaviour
**Target behaviour: Engaging in rapid self-referral in the event of changes in foot health**				
Belief that self-referral will do little to aid DFU healing [EO, LR] /Lack of confidence in DFU team [QR]	• Provide a rationale for the necessity of reporting any signs of foot damage, including evidence that this is effective for DFU healing	Psychological capability; Reflective motivation	Education; Persuasion	5.1 Information about health consequences<br>5.6 Information about emotional consequences
	• Provide patient stories demonstrating how rapid self-referral helped other patients to take control of their DFUs, and how they overcame previous frustrations with the DFU team and feelings that it was not worth it	Psychological capability; Reflective motivation; Social opportunity	Education; Persuasion; Modelling	5.1 Information about health consequences<br>6.2 Social comparison<br>6.3 Information about others' approval<br>9.1 Credible source
Lack of understanding regarding when to seek help and who to contact [EO, QR]	• Provide advice on when you may need to contact your diabetes team<br>• Advise patients to find out the contact details of their DFU team	Psychological capability	Education	4.1 Instructions on how to perform the behaviour

Barriers/*facilitator* to target behaviour	Intervention components	Target construct (BCW)	Intervention function (BCW)	Behaviour Change Technique (using BCTv1)
Difficulty accessing the DFU team and getting a quick appointment [EO, QR]	• Provide information on the national guidelines regarding timeline for referrals to DFU team<br>• Invite patients to refer to their local procedure for self-referrals given in their REDUCE action plan in the initiation phase<br>• Provide advice on how to communicate the reason for self-referral when contacting the DFU team	Psychological capability	Education	4.1 Instructions on how to perform the behaviour
Forgetting the contact details for the foot care team [EO]	• Invite patients to record the contact details of their foot care team and print this record to act as a reminder	Physical opportunity	Environmental structuring	7.1 Prompts/cues<br>12.5 Adding objects to the environment
Forgetting to contact their foot care team [EO]	• Invite patients to set up reminders to contact their foot care team if they record any signs of foot damage into the maintenance intervention and decide on the frequency of these reminders	Physical opportunity	Environmental structuring	7.1 Prompts/cues
Concerns about looking foolish or wasting the DFU team's time when reporting changes in foot health that turn out to be normal/<br>Not wanting to bother the DFU team [EO, QR]	• Reassure patients that health professionals would rather they were contacted early so they are better able to treat the DFU<br>• Provide patient stories on how other patients overcame feelings of being a burden<br>• Provide personalised feedback on whether or not they should self-refer, based on the answers they give to the foot health checklist	Psychological capability; Reflective motivation	Education; Persuasion	4.1 Instructions on how to perform the behaviour<br>6.3 Information about others' approval
**Target behaviour: Engaging in graded and regular physical activity**				
Belief that physical activity will do little to delay getting a DFU [EO, LR]<br><br>*Awareness of non-DFU related benefits of physical activity that are immediate and salient [EO]*	• Provide a rationale for the necessity of graded and regular physical activity and evidence that it is effective for delaying DFUs<br>• Provide a quiz about the benefits of physical activity for delaying DFUs, including other physical and mental benefits (e.g. improved sleep and energy, alleviation from aches and pains)	Psychological capability; Reflective motivation	Education; Persuasion	5.1 Information about health consequences<br>5.6 Information about emotional consequences
	• Provide patient stories demonstrating how graded and regular physical activity helped other patients to take control of their DFUs and led to other salient benefits	Reflective motivation; Social opportunity	Education; Persuasion; Modelling	5.1 Information about health consequences<br>6.2 Social comparison<br>6.3 Information about others' approval<br>9.1 Credible source

**Barriers/*facilitator* to target behaviour**	**Intervention components**	**Target construct (BCW)**	**Intervention function (BCW)**	**Behaviour Change Technique (using BCTv1)**
Concerns regarding the safety of physical activity [EO, QR]	• Reassure patients that gradual physical activity is safe (e.g. shouldn't cause too much shoe rubbing) and can be done when you do not have a DFU<br>• Address patients' individual physical activity concerns in the initiation phase	Psychological capability	Education	4.1 Instruction on how to perform the behaviour<br>5.1 Information about health consequences
Lack of belief in one's ability to engage in physical activity [LR] /Physical limitations (e.g. arthritis, breathlessness, foot discomfort/pain) [QR]/Bad weather [QR]<br><br>*Finding a suitable activity [QR]*	• Provide patient stories demonstrating how easy it was for other patients to engage in graded and regular physical activity, even though they are at high risk of developing DFUs or have health problems	Reflective motivation; Social opportunity	Persuasion; Modelling	6.2 Social comparison<br>6.3 Information about others' approval<br>9.1 Credible source
	• Provide a variety of examples of safe low-to-moderate physical activity, including activities that are non-weight bearing and can be done in bad weather	Psychological capability	Education	4.1 Instruction on how to perform the behaviour
Difficulties engaging in physical activity in the long-term [LR, QR]<br><br>*Social support [LR]; Provision of pedometers [LR, QR]; Integrating physical activity into your routine [QR]*	• Invite patients to set their own weekly physical activity goals<br>• Invite patients to self-monitor physical activity and provide personalised advice on how to modify goals based on self-monitoring<br>• Invite patients to make a physical activity action plan<br>• Invite patients to set easy-to-perform tasks and make them increasingly more difficult over time*	Reflective motivation; Psychological capability	Enablement; Training	1.1 Goal setting (behaviour)<br>1.4 Action planning<br>1.5 Review behaviour goal(s)<br>2.2 Feedback on behaviour<br>2.3 Self-monitoring of behaviour<br>8.7 Graded tasks*
	• Provide a free pedometer to those who would like one and encourage people to set daily step goals	Reflective motivation; Psychological capability; Physical opportunity	Enablement; Training; Environmental structuring	1.1 Goal setting (behaviour)<br>2.2 Feedback on behaviour<br>2.3 Self-monitoring of behaviour<br>12.5 Adding objects to the environment
	• Suggest that patients ask a friend/relative to exercise with them	Social opportunity	Enablement	3.1 Social support (unspecified)
	• Invite patients to regularly practice physical activity in the same context (e.g. after lunch)	Psychological capability; Automatic motivation	Training; Enablement	8.1 Behavioural practice/rehearsal<br>8.3 Habit formation

**Barriers/*facilitator* to target behaviour**	**Intervention components**	**Target construct (BCW)**	**Intervention function (BCW)**	**Behaviour Change Technique (using BCTv1)**
Forgetting to engage in physical activity [EO]<br><br>*Reminders [EO, LR]*	• Invite patients to set up email reminders to engage in physical activity and decide on the frequency of these reminders	Physical opportunity	Environmental structuring	7.1 Prompts/cues
**Target behaviour: Engaging in emotional management**				
Belief that emotional management will do little to delay getting a DFU or help with difficult emotions [EO; QR]	• Explain the necessity of emotional management for promoting engagement with the other foot care behaviours and provide evidence that they are effective for dealing with difficult emotions	Psychological capability; Reflective motivation	Education; Persuasion	5.1 Information about health consequences<br>5.6 Information about emotional consequences
	• Provide patient stories demonstrating how emotional management techniques helped other patients to take control of their DFUs	Psychological capability; Reflective motivation; Social opportunity	Education; Persuasion; Modelling	5.1 Information about health consequences<br>6.2 Social comparison<br>6.3 Information about others' approval<br>9.1 Credible source
Belief that emotional management is not relevant to them [QR]	• Provide emotional management as an optional part of the intervention<br>• Remind patients about emotional management at times of stress (e.g. if the foot health checklist highlights that they may have signs of getting a DFU)	N/A	N/A	N/A
Belief that the emotional management techniques do not fit with their preferable approach to emotional management [QR]	• Provide a range of techniques that may fit with a patients' preferred approach to emotional management (e.g. cognitive and behavioural techniques)	N/A	N/A	N/A
Lack of understanding regarding how to do the emotional management techniques [EO]	• Provide guidance on how to do the emotional management techniques	Psychological capability	Training	4.1 Instructions on how to perform the behaviour
	• Provide guided audio recordings of emotional management exercises	Physical opportunity	Environmental structuring	12.5 Adding objects to the environment
Lack of confidence in ability to practice emotional management techniques [EO]	• Provide patient stories demonstrating how easy it was for other patients to practice the emotional management techniques	Reflective motivation; Social opportunity	Persuasion; Modelling	6.2 Social comparison<br>6.3 Information about others' approval<br>9.1 Credible source

**Barriers/*facilitator* to target behaviour**	**Intervention components**	**Target construct (BCW)**	**Intervention function (BCW)**	**Behaviour Change Technique (using BCTv1)**
Difficulties engaging in emotional management in the long-term [EO]	• Invite patients to set their own emotional management practice goals	Reflective motivation	Enablement	1.1 Goal setting (behaviour)<br>1.4 Action planning<br>1.5 Review behaviour goal(s)
**Subsidiary behaviour: Engaging with the digital maintenance intervention**				
Low confidence in ability to use digital interventions [LR; QR]<br><br>*Technical support to use digital interventions [EO]*	• Health professionals introduce the digital maintenance intervention in the initiation phase and provide technical support as required<br>• Suggest that family and friends could assist the patient with digital intervention use, if appropriate	Physical capability; Psychological capability; Social opportunity	Training; Education; Enablement	3.2 Social support (practical)<br>4.1 Instructions on how to perform the behaviour
	• Health professionals will speak favourably about digital intervention use and outline its advantages<br>• Build patients' confidence in using the digital intervention by demonstrating how easy it is to use the intervention, and addressing any self-doubts*	Reflective motivation	Education; Persuasion	9.1 Credible source<br>15.1 Verbal persuasion about capability*
