# Supplementary material for: Intervention planning for the REDUCE maintenance intervention: a digital intervention to reduce reulceration risk among patients with a history of diabetic foot ulcers
Source: BMJ Open. 2018 May 18;8(5):e019865. doi: 10.1136/bmjopen-2017-019865 (PMC5961606; doi:10.1136/bmjopen-2017-019865)
Supplement: Author's manuscript [file bmjopen-2017-019865.draft_revisions.pdf]

BMJ Open

BMJ Open is committed to open peer review. As part of this commitment we make the peer review history of every article we publish publicly available.

When an article is published we post the peer reviewers' comments and the authors' responses online. We also post the versions of the paper that were used during peer review. These are the versions that the peer review comments apply to.

The versions of the paper that follow are the versions that were submitted during the peer review process. They are not the versions of record or the final published versions. They should not be cited or distributed as the published version of this manuscript.

BMJ Open is an open access journal and the full, final, typeset and author-corrected version of record of the manuscript is available on our site with no access controls, subscription charges or pay-per-view fees (http://bmjopen.bmj.com).

If you have any questions on BMJ Open's open peer review process please email info.bmjopen@bmj.com

Intervention planning for the REDUCE maintenance intervention: a digital intervention to reduce re-ulceration risk among patients with a history of diabetic foot ulcers

Journal:	*BMJ Open*
Manuscript ID	bmjopen-2017-019865
Article Type:	Research
Date Submitted by the Author:	29-Sep-2017
Complete List of Authors:	Greenwell, Kate; Centre for Clinical and Community Applications of Health Psychology, University of Southampton<br>Sivyer, Katy; Centre for Clinical and Community Applications of Health Psychology, University of Southampton<br>Vedhara, Kavita; University of Nottingham Division of Primary Care<br>Yardley, Lucy ; Centre for Clinical and Community Applications of Health Psychology, University of Southampton<br>Game, Frances; Derby Teaching Hospitals NHS Foundation Trust<br>Chalder, Trudie; King's College London, Department of Psychological Medicine<br>Richards, Gayle; Northern Devon Healthcare NHS Trust, Department of Diabetes<br>Drake, Nikki; Bristol Community Health<br>Gray, Katie; Derby Teaching Hospitals NHS Foundation Trust<br>Weinman, John; King's College London Institute of Pharmaceutical Science<br>Bradbury, Katherine; Centre for Clinical and Community Applications of Health Psychology, University of Southampton
**Primary Subject Heading**:	Diabetes and endocrinology
Secondary Subject Heading:	Qualitative research, Patient-centred medicine, Research methods
Keywords:	Diabetic foot < DIABETES & ENDOCRINOLOGY, Diabetes & endocrinology < INTERNAL MEDICINE, WOUND MANAGEMENT

SCHOLARONE™
Manuscripts

Intervention planning for the REDUCE maintenance intervention: a digital intervention to reduce re-ulceration risk among patients with a history of diabetic foot ulcers

Kate Greenwell[1]*[a], Katy Sivyer[1]*, Kavita Vedhara[2], Lucy Yardley[1], Frances Game[3], Trudie Chalder[4], Gayle Richards[5], Nikki Drake[6], Katie Gray[3], John Weinman[7], Katherine Bradbury[1]

*Kate Greenwell and Katy Sivyer contributed equally to this paper

[a]Corresponding author: Centre for Clinical and Community Applications of Health Psychology, University of Southampton, Shackleton Building, Highfield Campus, Southampton, SO17 1BJ, UK; Email: reduceproject@soton.ac.uk; Tel: +44 (0) 2380 5950

[1]Centre for Clinical and Community Applications of Health Psychology, University of Southampton, Southampton, UK

[2]Division of Primary Care, School of Medicine, University of Nottingham, Nottingham, UK.

[3]Derby Teaching Hospitals NHS Foundation Trust, Derby, UK

[4]Department of Psychological Medicine, King's College London, London, UK

[5]Department of Diabetes, Northern Devon Healthcare NHS Trust, Barnstaple, UK

[6]Bristol Community Health, Bristol, UK

[7]Institute of Pharmaceutical Science, King's College London, London, UK

Word count: 3972 words

For peer review only - http://bmjopen.bmj.com/site/about/guidelines.xhtml

ABSTRACT

Objectives: To develop a comprehensive intervention plan for the REDUCE maintenance intervention to support people with a history of diabetic foot ulcers (DFUs) to sustain behaviours that reduce re-ulceration risk.

Design: Theory-, evidence- and person-based approaches to intervention development were used. Evidence was collated from a scoping review of the literature and qualitative interviews with patients who have had DFUs (N=20). This was used to identify the psychosocial needs and challenges of this population, and barriers and facilitators to the intervention's target behaviours: regular foot checking, rapid self-referral in the event of changes in foot health, graded and regular physical activity, and emotional management. This evidence was then combined with expert consultation to develop brief 'guiding principles' for shaping intervention development. Theory-based 'behavioural analysis' and 'logic modelling' were used to map the evidence and intervention content onto behaviour change theory to comprehensively describe the intervention and its potential mechanisms of action.

Results: The evidence suggested that key challenges facing patients included uncertainty regarding when to self-refer, physical limitations that may affect foot checking and physical activity, and, for some, difficulties managing negative emotions following a DFU. Important considerations for the intervention design included a need to increase patients' confidence in making a self-referral and in using the MI, and a need to acknowledge that some intervention content that may be relevant to only a sub-set of patients (emotional management, physical activity). Intervention processes outlined in the behavioural analysis and logic model focused on increasing patients' skills, self-efficacy, knowledge, positive outcome expectancies, sense of personal control, social support, and physical opportunity.

For peer review only - http://bmjopen.bmj.com/site/about/guidelines.xhtml

Conclusions: This research provides a transparent description of the intervention planning for the REDUCE maintenance intervention. It provides insights into potential barriers and facilitators to the target behaviours and potentially useful behaviour change techniques to use in clinical practice.

STRENGTHS AND LIMITATIONS OF THIS STUDY

- This research will inform the development of a novel intervention to support the prevention and management of DFUs and is in keeping with recent NICE research priorities for the diabetic foot.
- The integration of theory- evidence- and person-based approaches provided complementary insights into how an intervention could be designed to maximise its acceptability, feasibility, and potential effectiveness.
- The REDUCE maintenance intervention plan is comprehensively described and the intervention's potential mechanisms of actions made explicit, thereby increasing transparency, and facilitating application of this intervention planning methodology by other intervention developers.
- Although the qualitative sample was representative of patients with a DFU (who tend to be older and may therefore be retired), few younger and employed people were recruited so their views remain less well understood.
- Although the rapid scoping review allowed scientific evidence to be quickly incorporated into the intervention plan at an early stage, it was not systematic, so it is possible that some literature may have been missed.

For peer review only

For peer review only - http://bmjopen.bmj.com/site/about/guidelines.xhtml

BACKGROUND

Foot ulceration is a common, chronic, and costly complication of diabetes.[1–3] Healing is slow and recurrence is common, with approximately 40% of patients re-ulcerating within 12 months.[4–6] The physical and emotional burden of ulceration is considerable; 20% of ulcers result in amputation and 32% of patients are depressed, which is associated with a threefold greater risk of mortality.[2,7] Although diabetic foot care has been deemed a priority,[2] there are a lack of evidence-based treatments that prevent ulceration. Systematic reviews have found no evidence that education alone improves clinical outcomes.[8–11] However, research suggests that psychosocial and behavioural factors may play a central role in healing and prevention.[12] NICE have consequently recommended the development of new interventions targeting such factors.[2]

'REDUCE' is a complex cognitive behavioural intervention [13] that aims to reduce re-ulceration risk and promote healing by modifying associated psychological and behavioural factors.[14] The intervention consists of two phases; an initiation phase of eight weekly sessions with a nurse or podiatrist to start psychological and behavioural change, and a maintenance phase involving two additional sessions held one and three months later to help sustain these changes. Although REDUCE was found to be acceptable and feasible for patients,[14] long-term maintenance of these changes may be more effective if the intervention were available indefinitely, and when patients require it. A digital maintenance intervention would provide a low-cost solution.

The key objective of the REDUCE maintenance intervention will be to provide support to people who have had diabetic foot ulcers (DFUs) to increase their ulcer free survival with limbs intact (i.e. the length of time a patient is free from ulcers without having had an amputation) through behaviour change and emotional management. It will support people to

For peer review only - http://bmjopen.bmj.com/site/about/guidelines.xhtml

maintain four behaviours targeted in the initiation phase: regular foot checking, rapid self-referral in the event of changes in foot health, graded and regular physical activity, and emotional management.

Published descriptions of complex interventions and their development process are often inadequate, providing readers with little understanding of what the intervention contains, how decisions regarding its development were made, and how the intervention is hypothesised to work.[11,15–17] This paper presents the full intervention planning process for the REDUCE maintenance intervention as an example of intervention planning methodology and to increase transparency regarding the intervention's content and hypothesised mechanisms of action.

METHODS AND RESULTS

Intervention planning methodology

The intervention planning used theory-, evidence- and person-based approaches.[13,18–20] The person-based approach recommends grounding intervention development in an in-depth understanding of the patient and their psychosocial context, gained through qualitative research.[18] A rapid scoping review of qualitative and quantitative literature was used to examine the behavioural and psychosocial needs, issues, and challenges of people who have had DFUs. This knowledge was combined with insights gained from a qualitative interview study that explored patients' perspectives on key content and design features for the maintenance intervention. Additional barriers and facilitators to the intervention's target behaviours and intervention content were identified through consultation with our multidisciplinary project team, including clinical and intervention development experts. All sources of evidence (i.e. scoping review, qualitative study results, expert opinion) were brought together to create 'guiding principles' that outline the intervention design objectives

For peer review only - http://bmjopen.bmj.com/site/about/guidelines.xhtml

and key intervention features. Theory-based ‘behavioural analysis’ and ‘logic modelling’ [17,20,21] were used to map the evidence and intervention content onto behaviour change theory to comprehensively describe the intervention and its potential mechanisms of action.

Qualitative and quantitative literature review

Purpose

To collate evidence examining the behavioural and psychosocial needs, issues, and challenges of people who have had DFUs.

Methods

A rapid scoping review of the qualitative and quantitative literature exploring patients’ and health professionals’ views and experiences of DFUs and their management was undertaken to inform the initial intervention plan. A search was undertaken in Web of Science combining the following terms (“diabetic foot ulcer”) AND (“physical activity” OR exercise), (“self-referral” OR “help seeking”), (check AND (foot OR feet)), and (“emotional management” OR “mood management”). Additional literature was identified through expert consultation and article reference lists. Data were extracted on research design, sample size, participants, and key findings. The key findings were organised into themes relating to the psychosocial and behavioural issues, needs, or challenges to be considered during intervention development.

Results

The review identified seven articles and highlighted seven themes relating to people’s beliefs around DFUs and foot care, challenges people face when engaging in the target behaviours, difficult emotions people may experience following a DFU, and concerns about digital interventions (Table 1).

For peer review only

Table 1 Key themes identified from the rapid scoping review of the psychosocial and behavioural issues, needs and challenges of people who have had DFUs

Key themes	Detail from the literature
Lack confidence in foot checking [22,23]	• Some people were uncertain about what a DFU was or looked like, what signs of DFUs to look out for, and when the DFU was serious enough to seek help from a health professional. Such uncertainties may lead to delays in seeking help.
Feelings of lack of control in preventing DFUs [22,23]	• Some people felt they had little or no control in preventing further DFUs, as DFUs still occurred even when they were engaging in foot care behaviours.<br>• Some people believed that they were unable to prevent DFUs.
Difficult emotions following a DFU [22,24–27]	• Some people were fearful or worried about developing further DFUs, losing limbs through amputation, and the impact a DFU reoccurrence might have on their lives.<br>• Some people felt down or had low self-esteem because of how the DFUs had negatively affected their everyday lives (e.g. loss of independence, inability to work and provide for the family, lifestyle changes).<br>• Some people felt a sense of hopelessness, anger, and frustration when DFUs developed despite their attempts to engage in foot care behaviours.<br>• Some people felt self-blame or guilt for not paying enough attention to their feet, not controlling their diabetes well, not following foot care advice, or not engaging in foot care behaviours, especially in the event of reoccurrence.<br>• Some people experienced social isolation (e.g. from restricted mobility, lack of employment) or felt a burden to others because they were dependent on them for daily activities (e.g. cooking and driving).<br>• Some people found it difficult to share their experiences of a DFU with friends and family.
Maintaining behaviours long-term may be challenging [22]	• Some people were not confident that they could maintain foot care behaviours in the long-term, with engagement likely to decrease over time.<br>• Some people were impatient to resume the physical activities they stopped when they had an active DFU, leading them to do too much activity and risk getting another DFU.
Physical limitations impeding foot	• Some people had joint mobility problems, neuropathy, and visual impairment that may prevent them engaging in

For peer review only - http://bmjopen.bmj.com/site/about/guidelines.xhtml

**Key themes**	**Detail from the literature**
checking [27,28]	foot care behaviours.
Concerns over using digital interventions [25]	• Some people felt they did not have the necessary computer skills for internet or computer-based interventions.

For peer review only

For peer review only - http://bmjopen.bmj.com/site/about/guidelines.xhtml

Qualitative interviews

Purpose

To explore the acceptability and feasibility of plans for the maintenance intervention from the perspective of people who have had DFUs; and to identify potential barriers and facilitators to its target behaviours.

Methods

Semi-structured interviews were undertaken with 20 adults with diabetes who had previously had a DFU. Interviews explored participants' views of the target behaviours, potential intervention features (e.g. foot checking reminders), and possible modes of intervention delivery, including booklet, website, computer tablet, and smartphones. Interviews also explored participants' views on the value of additional health professional input. Ideas for potential content and delivery modes were shown on prompt cards and participants were shown an example of an existing diabetes intervention [29] to demonstrate what a website intervention could look like. Interviews were piloted with two people who have had DFUs. See Appendix 1 for the interview schedule and prompt cards.

A total of 250 patients were contacted by letter by their local NHS podiatry service. Sixty-six patients (26%) expressed interest in the study, 53 of whom (21% of original mail-out) were eligible to participate. Eligible respondents were purposively sampled to represent a diverse set of ages (range: 45-91 years), genders, and internet use (Table 2). Interviews were carried out by KG and KS and took place at participants' homes (N=18) or the university (N=2). Participants were reimbursed for travel and given a £10 voucher. All interviews were recorded and transcribed. KG and KS used thematic analysis to identify potential barriers and facilitators to engaging with the target behaviours, and positive and negative perceptions of the potential intervention features and delivery modes. Ethical approval for this study was

For peer review only - http://bmjopen.bmj.com/site/about/guidelines.xhtml

gained from North West – Greater Manchester West Research Ethics Committee (17/NW/0024).

Table 2 Demographics of patients taking part in the qualitative interviews

**Sample Characteristics**	**Statistics**
**Basic Demographics**	**Mean (SD)**
Age	68.30 (11.54)
**Basic Demographics**	**N (%)**
Male	11 (55%)
Marital Status	
Married	7 (35%)
Single	6 (30%)
Widowed	4 (20%)
Divorced	3 (15%)
Employment Status	
Retired	15 (75%)
Redundant due to illness	3 (15%)
Housewife/husband	1 (5%)
Full-time employed	1 (5%)
Educational Status	
Secondary School	10 (50%)
College / Sixth Form / Professional Qualification	7 (35%)
Undergraduate	3 (15%)
**DFU History**	**Mean (SD)**
Years since first DFU (approx.)	6.81 (7.96)
Number of DFUs (approx.)	4.18 (3.86)
Months since last DFU (approx.)	14.65 (11.26)
Duration of last DFU in days (approx.)	298 (400.82)
**Internet Use**	**N (%)**
Access to internet at home	15 (75%)
Access to internet on tablet	7 (35%)
Access to internet on phone	3 (15%)
Frequency of access	

For peer review only - http://bmjopen.bmj.com/site/about/guidelines.xhtml

Never	3 (15%)
Less than once a month	3 (15%)
Once a week	1 (5%)
A few times a week	2 (10%)
Once a day	3 (15%)
Several times a day	8 (40%)

Results

The key findings are outlined below. Example quotes are in Table 3.

Table 3 Table of key issues arising from our qualitative study and illustrative quotes

**Issue arising from our qualitative study**	**Participant quotes**
Foot checking	
Some participants had physical limitations that make it difficult to check their feet.	"As you get older you're not so mobile so you can't see right underneath [your foot], so it's a bit of guesswork until you do go…to [the] podiatrist" (P10, Male)
Some people found it difficult to know what to look for when foot checking and when to self-refer.	"Recognising them [DFUs] I think is the hardest part" (P14, Male)<br>"Sometimes…I go [to the podiatrist] and it's not an ulcer…but I can't tell" (P8, Male)
A few participants found it difficult to keep up foot checking long-term.	"You kind of become rather lax about perhaps doing it [foot checking] properly" (P1, Male)
There were mixed views on foot checking reminders.	"I don't think I would need to be reminded. I'm doing it [foot checking] already, really" (P3, Female)<br>"It's nice to have a reminder. Sometimes you get a bit complacent and you think 'Oh, I'll do it next time'" (P10, Male)
Rapid self-referral	
Some participants found it difficult to contact and get an appointment with their DFU team.	"Sometimes you can't get appointments…By the time you are seeing somebody it's either through A&E, because you've been rushed in 'cause your foot's swollen up and changed colour" (P18, Female)
Some participants expressed concerns about self-referring.	"If you do that [point out changes in foot health] every visit and it's nothing to worry about, you're paranoid, micromanaging. But if you don't mention something you've seen previously, you're complacent and don't care about your health. You can't win" (P18, Female)
Some participants found it difficult to know which health professional to	"Who do you contact if you have a problem? Your own doctor? Or the nurse, diabetic nurse? Or the podiatrist?"

For peer review only - http://bmjopen.bmj.com/site/about/guidelines.xhtml

contact when reporting DFUs.	(P5, Male)
Physical activity	
Some participants have physical limitations that make it difficult to engage in physical activity.	"I get very breathless. I don't walk much at all. I know I should, but I don't" (P3, Female)
Some participants also expressed concerns about physical activity causing another DFU.	"Even though you might not have an ulcer, even if you go back to minimal activity…you can still get that ulcer come back" (P18, Female)
Some participants found it can be difficult to keep up with physical activity over time.	"It is easy to find something else to do [instead of physical activity]. You've got to be pretty disciplined" (P6, Female)
There were mixed views on pedometers.	"The pedometer is a really good idea though…It's like a game – you want to make sure you can get as many steps in" (P20, Female)<br><br>"[The pedometer is] almost like being spied on" (P14, Male)
Emotional management	
Emotional management was relevant and valued by some participants, but not everyone.	"I'm one o' these anxiety merchants, me. I worry for the world…so it'd [emotional management] be very helpful" (P10, Male)<br><br>"I don't think personally I would have taken it [emotional management] on board at all…it's not gonna make any difference to me…I just think I've got it [DFUs], I've got to put up with it…I don't want to sit on a couch breathing in and out, I want to get on and do something" (P2, Female)
Delivery methods	
Participants were positive about the idea of a website, but there were some concerns about computer literacy.	"Personally think the website would be far better than the booklet…It's prodding me to do it [use the intervention]…If it's in a leaflet, it just gets left" (P14, Male, internet user)<br><br>"I love…anything interactive like that [the quiz in the example website] I think is great…you feel part of it [the intervention], rather than just being dictated to…[the information] tends to sink in better" (P20, Female, internet user)<br><br>"If I was competent…I would do it on the computer. But I'm not competent" (P8, Male, infrequent internet user)
A booklet might be helpful for quick reference and for those who do not use the internet.	"A booklet is always there, you can always refer to it, you've got something in black and white" (P8, Male)
Delivering the intervention via smartphone was less acceptable.	"Mobile phone - you've got all the problems of the computer, but on a smaller screen…a lot of diabetics [have] got problems with their eyes as well" (P17, Male)

For peer review only - http://bmjopen.bmj.com/site/about/guidelines.xhtml

Participants liked the idea of additional health professional support, but not for the intended purpose of supporting behaviour maintenance.	"It'd [additional health professional support] give me the confidence to know that 'well, I am alright with my foot as it is'…because you can get a bit paranoid over it [your foot health]" (P17, Male)<br><br>"They could give…one-to-one advice on…is there anything else that you could do…better than what I'm doing myself" (P3, Female)

Regular foot checking: Generally, participants perceived foot checking as acceptable and important for preventing DFUs. Many found foot checking easy to do and already checked their feet regularly. However, many participants reported physical limitations (e.g. limited mobility) and other physical barriers (e.g. wearing casts or bandages) that restricted foot checking. While some people found it easy to spot changes in foot health, others reported difficulties knowing what to look for and in judging whether any changes were problematic. A few described how it is easy to become lax over time, forgetting to check feet regularly or not thoroughly checking. Participants identified several facilitators to foot checking, including using a mirror to check feet, getting someone else to check, and integrating foot checking into everyday routine (e.g. when putting on socks).

When discussing the planned intervention features, some people believed it would be useful to set up regular email foot checking reminders because it is easy to forget. Others felt reminders could be irritating or were unnecessary, as they, or their podiatrist, already regularly checked their feet. Generally, people thought it would be helpful to be able to make a note of any changes in their foot health to track changes in foot health over time. A few people felt this was unnecessary because they already checked their feet regularly, and knew what to look for, or believed it would be difficult to remember to note down changes.

Rapid self-referral in the event of changes in foot health: Most participants were positive about self-referral, viewing it as important. However, many people found it difficult to contact their DFU team. Long waiting times left some participants worried about how their foot health might decline in the meantime, which led one person to treat their feet themselves,

For peer review only

For peer review only - http://bmjopen.bmj.com/site/about/guidelines.xhtml

instead of self-referring. In contrast, some participants reported the opposite and found it easy to get an appointment with their DFU team. A few participants were unsure which health professional to contact when reporting DFUs (e.g. podiatrist, diabetes nurse, GP). Some expressed concerns about looking foolish or wasting health professionals' time when self-referring for changes in foot health that turned out to be normal. One person had trouble with getting her concerns taken seriously and a few people worried about being a burden to health professionals. Some participants wanted reassurance from health professionals that it was right to have sought help.

Graded and regular physical activity: Most participants were positive about physical activity, stating that they would like to or were already doing it. People generally viewed physical activity as important for general health and diabetes management. However, many participants reported physical limitations (e.g. pain, fatigue) or diabetic complications (e.g. neuropathy, residual damage to feet from previous DFUs) that made it difficult to be active. Participants reported that it was important to find the right activity to overcome their physical limitations, suggesting activities that did not put pressure on their feet, such as seated exercises. Some were concerned that physical activity might cause another DFU or exacerbate other health conditions.

Some participants stated that it could be difficult to maintain physical activity. A few mentioned that integrating physical activity into their daily routine (e.g. getting off the bus one stop early) and positive encouragement helped. Participants viewed self-monitoring, goal setting, and pedometers as helpful for maintaining motivation. However, some people disliked the idea of being 'spied on' or told what to do, expressed doubts about the accuracy of pedometers, or were unsure whether they would use them.

For peer review only - http://bmjopen.bmj.com/site/about/guidelines.xhtml

For peer review only

Emotional management: Over half of participants viewed emotional management positively and reported experiencing low mood, frustration, anger, and stress either during or after a DFU. Others had not experienced such emotions relating to their DFUs and viewed emotional management as irrelevant. A few people viewed emotional management negatively due to previous negative experiences. For example, some had experienced unhelpful reactions from doctors when discussing emotions, disliked talking about their feelings in counselling, or had received unhelpful information about emotional management (e.g. being given advice that did not consider their physical limitations). Some expressed a lack of understanding about how the emotional management would help or perceived it as contrary to their personal style of managing emotions (i.e. ignoring their problems, 'getting on with it').

Intervention delivery methods: Most participants were positive about the idea of the intervention being delivered via a booklet. Booklets were perceived as quick and easy to refer to, portable, and easily shared or distributed (e.g. with relatives or picked up from clinics). However, some participants commented that booklets were easily misplaced or forgotten. Most internet users reacted positively to the idea of a website, mainly because it was easy to access, convenient, and had interactive features (e.g. quizzes, email reminders). Nonetheless, non-users and a few infrequent internet users expressed concern about their own computer literacy. Some participants disliked reading on a computer screen and a few participants had concerns about security of web interventions. However, when participants were shown the example website, they generally viewed it positively, stating that it looked easy to use. A few participants would have liked to access the intervention using a computer tablet as they already used one or knew people who did. Most viewed delivery using a smartphone negatively because of their limited use of phones or difficulties with using small screens due to poor eyesight (caused by diabetes). A few participants commented that it might be helpful to deliver the intervention through multiple modes (booklet, website, tablet, or phone).

For peer review only - http://bmjopen.bmj.com/site/about/guidelines.xhtml

Generally, participants were in favour of additional health professional support. However, they interpreted this as support to gain reassurance about the status of their foot health, and advice on foot care or when to self-refer (which would be covered in the website/booklet), rather than support to raise motivation for engaging with the target behaviours. Very few participants said they might use this support to answer questions about information in the booklet or website.

Explanations of how the evidence from the scoping review and qualitative study informed intervention planning are provided in the next sections on Guiding Principles and Behavioural Analysis.

Guiding principles

Purpose

To develop brief guiding principles,[18] to be consulted throughout intervention development to ensure that the intervention is underpinned by a coherent focus.

Methods

Drawing upon the findings from our scoping review and qualitative study, key characteristics of target users and the key behavioural issues, needs and challenges the intervention must address were described. From this, guiding principles were created, which outline the intervention design objectives that will address these key behavioural issues, needs and challenges, and the key intervention features designed to achieve these objectives.

Results

People who have had DFUs can feel they have little or no control over preventing DFUs, as DFUs can occur even when people are engaging in foot care behaviours. This leaves people feeling hopeless and frustrated.[22] Some people may feel self-blame or guilt for not engaging in foot care behaviours, especially in the event of reoccurrence.[22] Therefore, one

For peer review only - http://bmjopen.bmj.com/site/about/guidelines.xhtml

design objective was to reduce feelings of hopelessness, frustration, self-blame, and guilt following a DFU.

People may be uncertain about the signs of a DFU and when to seek help from a health professional.[23] Our qualitative study highlighted that some people were concerned about looking foolish, being a burden, or wasting healthcare professionals' time if changes in their feet turn out to be normal. This may delay help seeking. Therefore, one design objective was to build patients' confidence in making a self-referral.

This population are likely to have physical limitations and/or co-morbidities. Our qualitative study highlighted that these challenges may make it difficult for people to engage in foot checking and physical activity. They may also be reluctant to increase activity in case it causes re-ulceration. Thus, one design objective was to acknowledge that patients may have physical limitations that make it difficult to engage in foot checking and physical activity.

Our scoping review highlighted that people may experience difficult emotions following a DFU.[22,24–27] However, some participants in our qualitative research did not experience such emotions and, therefore, did not perceive emotional management as useful. Therefore, one design objective was to acknowledge that emotional management may not be relevant for all patients.

As the physical activity and emotional management content was not relevant to all patients, these components were made optional, rather than mandatory, to avoid discouraging patients from engaging in the other target behaviours if they do not want to increase physical activity or engage in emotional management.

In our qualitative study, many reacted positively to the idea of a web-based intervention, but some participants expressed concerns about their computer literacy. These concerns were also evident in the literature.[25] Therefore, one design objective was to ensure people feel

For peer review only - http://bmjopen.bmj.com/site/about/guidelines.xhtml

confident in using the maintenance intervention. We decided to deliver the intervention using a website and provide key information and advice in a booklet for quick reference and for non-internet users. At the preceding initiation phase, health professionals will address concerns, and speak favourably of the digital intervention to encourage use. Table 4 details the REDUCE maintenance intervention guiding principles.

For peer review only

For peer review only - http://bmjopen.bmj.com/site/about/guidelines.xhtml

Table 4 The guiding principles for the development of the REDUCE maintenance intervention

**Intervention design objectives**	**Key features**
To reduce feelings of hopelessness, frustration, self-blame, and guilt following a DFU	• Emphasise target behaviours that patients can engage in to reduce their chances of getting another DFU, while acknowledging that there are precipitating factors (e.g. increased age, neuropathy, foot shape) that are out of their control.<br>• Enhance patients' confidence in the target behaviours (e.g. by providing a rationale for the necessity of the target behaviours, scientific evidence that behaviours are effective, patient stories, and a quiz on the benefits of the behaviours).<br>• Validate patients' feelings of frustration and hopelessness if a DFU does reoccur and avoid arguments that may be viewed as blaming patients for this re-occurrence.<br>• Provide links to emotional management techniques that can help people to manage difficult emotions.
To build patients' confidence in making a self-referral	• Provide links to foot checking training (e.g. by providing information and photographs on what DFUs look like, what signs to look out for, and how often feet should be checked with guided practice).<br>• Provide reassurance that self-referral is necessary (e.g. through a foot health checklist that provides personalised feedback on whether or not patients should self-refer, based on their symptoms).<br>• Address concerns around looking foolish or wasting the DFU team's time when self-referring (e.g. a) emphasise that the DFU team would rather they were contacted early so they are better able to treat any DFUs, b) provide patient stories about how other patients overcame feelings of burden).
To acknowledge that patients may have physical limitations that make it difficult to engage in foot checking and physical activity	• Provide guidance on how to check your feet if you have physical limitations, including using a mirror to check the bottom of your feet and asking someone else to check for you.<br>• Make intervention content on physical activity optional.<br>• Provide guidance about a variety of safe and low impact physical activities to enable patients to find an activity that is suitable for them.<br>• Address physical activity concerns all the way through the intervention (i.e. in the maintenance intervention and prior initiation phase) (e.g. by providing information about the safety of physical activity, patient stories about how other patients overcame these barriers).

For peer review only - http://bmjopen.bmj.com/site/about/guidelines.xhtml

**Intervention design objectives**	**Key features**
To acknowledge that emotional management may not be relevant for all patients	• Make intervention content on emotional management optional.<br>• Emphasise that some people, but not everyone, might experience difficult emotions following a DFU to avoid excluding those who may not relate to this content.<br>• Provide a variety of brief emotional management techniques (e.g. CBT, mindfulness techniques) to allow each person to find a technique that fits with their own personal style of managing emotions.
To ensure patients feel confident in using the maintenance intervention	• Keep website navigation simple and follow guidelines for maximising website usability.<br>• Health professionals at the prior initiation phase will provide technical support, address self-doubts, and speak favourably of the digital intervention to encourage use.<br>• Encourage friends and family to assist people with website use, if appropriate.<br>• Provide a booklet for quick reference and for those who do not have access to the internet.

For peer review only - http://bmjopen.bmj.com/site/about/guidelines.xhtml

For peer review only

Behavioural analysis

Purpose

To use behaviour change theory to systematically describe the maintenance intervention content, identify potential determinants of behaviour (i.e. what needs to change for a behaviour to occur), and map it onto the evidence derived from our scoping review, our qualitative study, and expert consultation.

Methods

Behavioural analysis involves comprehensively mapping out the elements of an intervention, linking the evidence-base to behaviour change theory and the intervention components. Providing a clear description of the intervention is essential for replication in research and practice, data extraction in systematic reviews, and process evaluation planning.[13,16,17] The Behaviour Change Wheel (BCW [30,31]) and Behaviour Change Techniques Taxonomy (BCTv1 [32]) were developed to standardise the classification and description of complex interventions and help identify an intervention's 'active ingredients' and behavioural determinants. Such standardisation provides a common language to avoid any confusion that may occur when different terminology are used for the same intervention technique or different techniques are referred to using the same terminology.[33] The BCW draws upon the COM-B model, which argues that behaviour is influenced by an individual's Capability, Opportunity, and Motivation to change behaviour.[31]

In addition to the four target behaviours identified from the outset, the behavioural analysis also identified one subsidiary behaviour (engaging with the digital MI) that is necessary to enact these target behaviours. Barriers and facilitators for each behaviour were identified from the primary qualitative research, scoping review, and expert opinion from the multidisciplinary project team. Intervention components that addressed each barrier and facilitator were selected. These components are reported using patient-centred, autonomy-supportive language to

For peer review only - http://bmjopen.bmj.com/site/about/guidelines.xhtml

emphasise the importance of delivering these components in a way that will enhance intrinsic motivation and ensure a positive intervention experience.[18] The intervention components were coded using the BCTv1 and mapped onto the BCW to identify their corresponding intervention function (ways an intervention can change behaviour, e.g. 'education'), and target construct (what needs to change for the behaviour to occur, e.g. 'psychological capability'). The BCTv1 and BCW were then examined to check for potentially useful additional intervention functions, target constructs, or behaviour change techniques.

Results

The behavioural analysis is presented in Appendix 2. The maintenance intervention will target all six behavioural sources included in the BCW (physical and psychological capability, reflective and automatic motivation, and physical and social opportunity), and employ six different BCW intervention functions (education, persuasion, modelling, training, enablement, environmental restructuring) using 18 different BCTs. Intervention components that received a mixed reaction from our qualitative research participants (i.e. foot checking reminders, pedometers) were made optional to promote patient autonomy.

Although participants would have liked additional health professional support, the support participants wanted was more clinical in nature, which would be provided in the website/booklet. Therefore, additional health professional support was not included in the intervention plan. One issue that arose from our qualitative study could only be addressed to a limited degree by the maintenance intervention, namely the difficulties people experienced contacting, and getting an appointment, with their DFU team. This will be addressed by educating patients about the national guidelines and local procedures for self-referrals, and how to communicate the reason for self-referral to their DFU team. However, improving local self-referral pathways or modifying health professionals' behaviour is outside of the scope of this intervention.

For peer review only - http://bmjopen.bmj.com/site/about/guidelines.xhtml

Logic model

Purpose

To model the hypothesised mechanisms of action of the maintenance intervention (i.e. how it is thought to work).[17,20,21]

Methods

The logic model draws together findings from the scoping review, qualitative study, and behavioural analysis into a testable model that outlines how the different intervention components are hypothesised to impact on subsequent components and ultimately affect outcomes.

Results

The logic model (Figure 1) can be broken down into three major components.

Intervention techniques and processes: The intervention techniques summarise the behaviour change techniques outlined in the behavioural analysis and the seven processes they are hypothesised to affect: skills, self-efficacy, knowledge, positive outcome expectancies, sense of personal control, social support and physical opportunity. These are the psychosocial factors that need to be modified for the intervention's target behaviours to change. Each set of intervention techniques is hypothesised to mainly affect one of these processes, which subsequently affect one or more of the intervention's target behaviours. They are organised in order of importance, with more integral processes that were consistently identified as key in the scoping review and qualitative study at the top and less integral processes at the bottom (e.g. optional features).

Purported mediators: Purported mediators are the target behaviours of the intervention that are hypothesised to directly affect DFUs in the long-term. These behaviours are divided into 'core behaviours' that are hypothesised to be most important in determining DFU outcomes

For peer review only - http://bmjopen.bmj.com/site/about/guidelines.xhtml

(foot checking, rapid self-referral), and 'optional behaviours' that are only relevant for some patients (physical activity, emotional management). These behaviours' may impact either directly, as in the case of physical activity, or indirectly, via their effect on the other target behaviours, as is the case in emotional management. Emotional management is hypothesised to have an indirect effect on the other behaviours due to the negative effects that low mood (or negative thoughts) can have on behavioural engagement.

Outcomes: The logic model specifies three outcomes that the intervention is ultimately trying to change, the primary outcome of interest (ulcer free survival with limbs intact), and two interim outcomes that may be affected by the target behaviours and may, directly or indirectly, affect the primary outcome (severity of DFU at presentation and time taken for DFU healing in the event of a recurrence).

DISCUSSION

This paper describes the use of theory-, evidence- and person-based approaches to developing an intervention plan for the REDUCE maintenance intervention, an intervention that aims to reduce re-ulceration risk by supporting patients to maintain behaviour change and emotional management. These different approaches provided complementary insights into how the intervention could be designed to maximise its acceptability, feasibility, and effectiveness. Our scoping review and qualitative study deepened our understanding of the psychological and behavioural needs of people who have had DFUs and highlighted several barriers and facilitators to the intervention's target behaviours (e.g. lack of knowledge regarding what to look for when foot checking and when to self-refer). It also highlighted important advantages of, and barriers to, successful use of different intervention delivery methods (e.g. lack of confidence in ability to use digital interventions). Our guiding principles succinctly summarised the distinctive design objectives and features of the maintenance intervention,

For peer review only

For peer review only - http://bmjopen.bmj.com/site/about/guidelines.xhtml

while our behavioural analysis and logic modelling comprehensively described the intervention and its potential mechanisms of action.

This is the first paper to use this methodology to provide a comprehensive plan of a DFU intervention. Transparent reporting of the intervention planning process will allow other researchers to easily understand how this methodology could be applied to different intervention contexts. The use of primary qualitative research allowed us to understand patients' views on the delivery methods for behaviour change interventions and three behaviours that have received little attention in the DFU literature to date: engaging in rapid self-referral, graded and regular physical activity, and emotional management. For example, participants had mixed reactions to some behaviours (i.e. physical activity and emotional management) and design features (e.g. email reminders), which were subsequently made optional. Participants also reported experiencing difficulties with accessing their DFU team when self-referring. Future research should further explore and address any professional and organisational barriers to self-referral.

The qualitative research used purposive sampling which enabled us to explore the acceptability and feasibility of a digital intervention across a diverse set of people, including those who were frequent and infrequent internet users. Although the sample was representative of the population of people with DFUs (who tend to be older [34] and may therefore be retired), it would be helpful to explore the views of younger and employed people, as they may report different barriers to behaviour change. The rapid scoping review allowed scientific evidence to be quickly incorporated into the intervention plan, but it was not systematic, so it is possible that some literature was missed.

Recent NICE guidelines for the prevention and management of diabetic foot problems [2] identified a need to develop and evaluate new interventions targeting psychological and

For peer review only - http://bmjopen.bmj.com/site/about/guidelines.xhtml

behavioural factors. Our research has provided a plan for such an intervention, as well as identified potential barriers to behaviour change and behaviour change techniques that are likely to be useful within clinical practice. In future work, we intend to use this intervention plan to develop the maintenance intervention and then conduct an effectiveness trial to evaluate the effectiveness and cost-effectiveness of the entire REDUCE intervention, whilst also examining if the intervention works as hypothesised.

ACKNOWLEDGMENTS

The research team acknowledges the support of the National Institute of Health Research Clinical Research Network (NIHR CRN). We are grateful to the members of the REDUCE PPI panel for their valuable input on the study materials and interview schedule, and the participants who took part in this research.

COMPETING INTERESTS

All authors have completed the ICMJE uniform disclosure form at www.icmje.org/coi_disclosure.pdf and declare: KG, KS, KV, LY, FG, TC, ND, GR and KB had financial support from NIHR for the submitted work; TC had other support from NIHR; no financial relationships with any organisations that might have an interest in the submitted work in the previous three years; and no other relationships or activities that could appear to have influenced the submitted work.

FUNDING

This article summarises independent research funded by the National Institute for Health Research (NIHR) under its Programme Development Grants Programme (Grant Reference Number RP-DG-0615-10005). The views expressed are those of the author(s) and not necessarily those of the NHS, the NIHR or the Department of Health.

For peer review only - http://bmjopen.bmj.com/site/about/guidelines.xhtml

CONTRIBUTIONS

All authors designed the study. KG, KS, KB, and LY led the intervention planning, with input from the other co-authors. KG and KS were responsible for recruitment, carrying out the interviews, and analysing the data, with support from KB. KG and KS jointly drafted the manuscript with initial support from KB and LY. All authors critically reviewed the manuscript, contributing important intellectual content, and approved the final manuscript.

DATA SHARING STATEMENT

No additional data are available.

REFERENCES

1 Singh N, Armstrong DG, Lipsky BA. Preventing foot ulcers. *J Am Med Assoc* 2005;293:217–28. doi:10.1001/jama.293.2.217

2 NICE. Diabetic foot problems: Prevention and management. 2015.https://www.nice.org.uk/guidance/ng19 (accessed 28 Sep 2017).

3 Kerr M. Improving footcare for people with diabetes and saving money: An economic study in England. 2017.https://diabetes-resources-production.s3-eu-west-1.amazonaws.com/diabetes-storage/migration/pdf/Diabetic%2520footcare%2520in%2520England%2C%2520An%2520economic%2520case%2520study%2520%28January%25202017%29.pdf (accessed 28 Sep 2017).

4 Margolis D, Kantor J, Berlin J. Healing of diabetic neuropathic foot ulcers receiving standard treatment. *Diabetes Care* 1999;22:692–5.

5 Boulton AJM. The diabetic foot: Grand overview, epidemiology and pathogenesis. *Diabetes Metab Res Rev* 2008;24:S3–6. doi:10.1002/dmrr.833

For peer review only - http://bmjopen.bmj.com/site/about/guidelines.xhtml

6 Armstrong DG, Boulton AJM, Bus SA. Diabetic foot ulcers and their recurrence. *N Engl J Med* 2017;376:2367–75. doi:10.1056/NEJMra1615439

7 Ismail K, Winkley K, Stahl D, *et al.* A cohort study of people with diabetes and their first foot ulcer. *Diabetes Care* 2007;30:1473–9. doi:10.2337/dc06-2313

8 Mason J, O'Keeffe C, McIntosh A, *et al.* A systematic review of foot ulcer in patients with Type 2 diabetes mellitus. I: Prevention. *Diabet Med* 1999;16:801–12. doi:10.1046/j.1464-5491.1999.00133.x

9 Dorresteijn JAN, Kriegsman DMW, Assendelft WJJ, *et al.* Patient education for preventing diabetic foot ulceration. *Cochrane Database Syst Rev* 2014:CD001488. doi:10.1002/14651858.CD001488.pub2

10 Hunt DL. Diabetes: Foot ulcers and amputations. *BMJ Clin Evid* 2010;2009:602.

11 Hoogeveen RC, Dorresteijn JA, Kriegsman DM, *et al.* Complex interventions for preventing diabetic foot ulceration. *Cochrane Database Syst Rev* 2015:CD007610. doi:10.1002/14651858.CD007610.pub3

12 Vileikyte L. Psychosocial and behavioral aspects of diabetic foot lesions. *Curr Diab Rep* 2008;8:119–25. doi:10.1007/s11892-008-0022-1

13 Craig P, Dieppe P, Macintyre S, *et al.* Developing and evaluating complex interventions: New guidance. 2008.www.mrc.ac.uk/complexinterventionsguidance (accessed 28 Sep 2017).

14 Vedhara K, Beattie A, Metcalfe C, *et al.* Development and preliminary evaluation of a psychosocial intervention for modifying psychosocial risk factors associated with foot re-ulceration in diabetes. *Behav Res Ther* 2012;50:323–32. doi:10.1016/j.brat.2012.02.013

For peer review only - http://bmjopen.bmj.com/site/about/guidelines.xhtml

15 Michie S, Abraham C. Advancing the science of behaviour change: A plea for scientific reporting. *Addiction* 2008;103:1409–10. doi:10.1111/j.1360-0443.2008.02291.x

16 Michie S, Abraham C. Interventions to change health behaviours: Evidence-based or evidence-inspired? *Psychol Health* 2004;19:29–49. doi:10.1080/0887044031000141199

17 Moore G, Audrey S, Barker M, *et al.* Process evaluation of complex interventions: UK medical research council (MRC) guidance. 2014.https://www.mrc.ac.uk/documents/pdf/mrc-phsrn-process-evaluation-guidance-final/ (accessed 3 Mar 2017).

18 Yardley L, Morrison LG, Bradbury K, *et al.* The person-based approach to intervention development: Application to digital health-related behavior change interventions. *J Med Internet Res* 2015;17:e30. doi:10.2196/jmir.4055

19 Kok G, Schaalma H. Using theory in psychological interventions. In: Michie S, Abraham C, eds. Health Psychology In Practice. Oxford: Blackwell Publishing 2004:203–29. doi:10.1002/9780470694008.ch11

20 Band R, Bradbury K, Morton K, *et al.* Intervention planning for a digital intervention for self-management of hypertension: A theory-, evidence- and person-based approach. *Implement Sci* 2017;12:25.

21 Baxter SK, Blank L, Woods HB, *et al.* Using logic model methods in systematic review synthesis: describing complex pathways in referral management interventions. *BMC Med Res Methodol* 2014;14:62. doi:10.1186/1471-2288-14-62

22 Beattie AM, Campbell R, Vedhara K. 'What ever I do it's a lost cause.' The emotional

For peer review only - http://bmjopen.bmj.com/site/about/guidelines.xhtml

and behavioural experiences of individuals who are ulcer free living with the threat of developing further diabetic foot ulcers: A qualitative interview study. *Heal Expect* 2014;17:429–39. doi:10.1111/j.1369-7625.2012.00768.x

23 Searle A, Gale L, Campbell R, *et al.* Reducing the burden of chronic wounds: Prevention and management of the diabetic foot in the context of clinical guidelines. *J Health Serv Res Policy* 2008;13:82–91. doi:10.1258/jhsrp.2008.008011

24 Hjelm K, Nyberg P, Apelqvist J. Gender influences beliefs about health and illness in diabetic subjects with severe foot lesions. *J Adv Nurs* 2002;40:673–84. doi:10.1046/j.1365-2648.2002.02427.x

25 Burnside J, Wells W, Smith M, *et al.* Education for the prevention of foot ulcers: Views of those with neuropathy. 2006.

26 Kinmond K, McGee P, Gough S, *et al.* 'Loss of self': A psychosocial study of the quality of life of adults with diabetic ulceration. *World Wide Wounds* 2003;13:6–16. doi:10.1016/S0965-206X(03)80025-6

27 Searle A, Campbell R, Tallon D, *et al.* A qualitative approach to understanding the experience of ulceration and healing in the diabetic foot: Patient and podiatrist perspective. 2005;66:1–12.

28 Pollock RD, Unwin NC, Connolly V. Knowledge and practice of foot care in people with diabetes. *Diabetes Res Clin Pract* 2004;64:117–22. doi:10.1016/j.diabres.2003.10.014

29 Rowsell A, Muller I, Murray E, *et al.* Views of people with high and low levels of health literacy about a digital intervention to promote physical activity for diabetes: A qualitative study in five countries. *J Med Internet Res* 2015;17:e230.

For peer review only

For peer review only - http://bmjopen.bmj.com/site/about/guidelines.xhtml

doi:10.2196/jmir.4999

30 Michie S, Atkins L, West R. *The Behaviour Change Wheel: A Guide to Designing Interventions*. London: Silverback Publishing 2014

31 Michie S, Van Stralen MM, West R. The behaviour change wheel: A new method for characterising and designing behaviour change interventions. *Implement Sci* 2015;6:42.

32 Michie S, Richardson M, Johnston M, *et al.* The behavior change technique taxonomy (v1) of 93 hierarchically clustered techniques: Building an international consensus for the reporting of behavior change interventions. *Ann Behav Med* 2013;46:81–95. doi:10.1007/s12160-013-9486-6

33 Michie S, Wood CE, Johnston M, *et al.* Behaviour change techniques: The development and evaluation of a taxonomic method for reporting and describing behaviour change interventions (a suite of five studies involving consensus methods, randomised controlled trials and analysis of qualitative da. *Heal Technol Assess* 2015;19:1–188. doi:10.3310/hta19990

34 Clincal Audit and Registries Management Service. National Diabetes Foot Care Audit Report 2014-2015. 2016.http://content.digital.nhs.uk/catalogue/PUB20343/nati-diab-foot-care-audit-14-15-rep.pdf (accessed 28 Sep 2017).

FIGURE LEGEND

Figure 1 REDUCE Maintenance Intervention Logic Model

SUPPLEMENTARY MATERIAL

Appendix 1: Interview schedule and prompt cards

Appendix 2: Behavioural analysis table

For peer review only - http://bmjopen.bmj.com/site/about/guidelines.xhtml

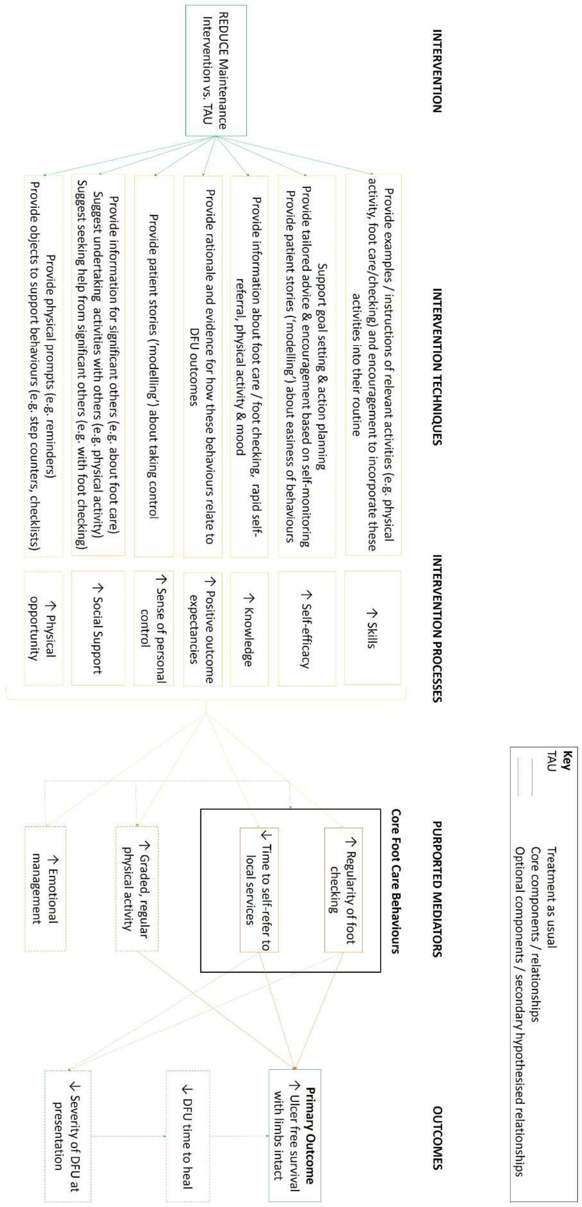

Figure 1 REDUCE Maintenance Intervention Logic Model

119x248mm (600 x 600 DPI)

APPENDIX 1: INTERVIEW SCHEDULE AND PROMPT CARDS

Interview schedule

Section 1: Context

Q1. Can you tell me a bit about what it has been like for you to have a foot ulcer?
Q2. Can you tell me about anything that you do to look after your feet currently?

Section 2: Acceptability and feasibility of the maintenance intervention

[Provide explanation of initiation phase and maintenance intervention]

1. Content of website/booklet

Card 1 (foot checking):

Q3. What do you think about these ideas? What do you like about these ideas? What do you dislike about these ideas?
Q4. The foot checker will ask you to check your feet every day for foot damage. What things might make it difficult for you to do this? Do you have any concerns about checking your feet every day?

Card 2 (help-seeking):

Q5. What do you think about these ideas? What do you like about these ideas? What do you dislike about these ideas?
Q6. The website may ask you to contact your diabetes team if you are concerned about any foot damage you have. What things might make it difficult for you to do this? Do you have any concerns about doing this?

Card 3 (physical activity):

Q7. What do you think about these ideas? What do you like about these ideas? What do you dislike about these ideas?
Q8. What things might make it difficult for you to get more active? Do you have any concerns about getting more active?

Card 4 (dealing with feelings):

Q9. What do you think about these ideas? What do you like about these ideas? What do you dislike about these ideas? Is there anything you do at the moment that helps you when you feel stressed / low?

Additional content:

Q10. What other things could we do to help you to look after your feet?

2. Delivery formats

Q11. What are your thoughts about the long-term support for maintaining habits being provided in **a booklet**? What do you like about this idea? What do you dislike about this idea?

For peer review only - http://bmjopen.bmj.com/site/about/guidelines.xhtml

Q12. What are your thoughts about the long-term support for maintaining habits being provided through **a website**? What do you like about this idea? What do you dislike about this idea?

Q13. If we were to deliver the programme through a website, what do you think about the idea of using **a computer or tablet**, such as an iPad, to access the website?

Q14. What do you think about the idea of using **a mobile phone** to access the website?

Card 5 (optional health professional support):

Q15. What do you think about this idea? What do you like about this idea? What do you dislike about this idea?

Q16. [If optional support would be helpful] What would you like to talk to the health professional about? Why would this be helpful?

Q17. You could contact the health professional in person, over the phone, and by email. Which one of these options would you prefer? Why?

Prompt Cards

Card 1 – Check your feet regularly

Why is this important?

It can be difficult to know when you might be developing a foot ulcer because some of the initial signs can be very small and hard to spot. It is important to examine your feet regularly so you are better able to spot any changes in your feet.

The website or booklet will:

- Ask you to check your feet every day
- Allow you to make a note of any changes in your feet

Set up regular reminders to check your feet which the foot checker can send to you by email or text messages to your mobile phone

Card 2 – What to do if you spot any foot damage

Why is this important?

It is important to report any changes in your feet to your diabetes team as soon as possible. This will allow them to check your feet and see if you need any treatment. The quicker your feet are treated, the more likely that any damage to your feet will heal.

The website or booklet will:

- Give you personalised advice on what to do if you spot any changes in your feet
- Advise you when you may need to contact your diabetes team

For peer review only

Card 3 – Getting active

Why is this important?

When you don't have a foot ulcer, it is safe and important to be mobile and active. This is because being active will improve your circulation and blood sugar, and reduce your chances of getting another ulcer. It is important that you pace your activity – little and often is best. Irregular activity (doing nothing and then doing too much) can be harmful because it increases the chances of injuring your feet.

The website or booklet will:

- Help you to slowly increase physical activity, such as using an exercise bike, seated exercise, walking, or any other things you like to do to be active.
- Set weekly physical activity goals, for example, going for a walk once or a few times a week.
- Ask you to enter in information about how you got on with your goals each week and provide you with personalised advice based on your progress.

A free step counter (or pedometer) that clips onto your belt and counts how many steps you take. You can use this to set yourself daily or weekly step goals if you would like to.

Card 4 – Dealing with your feelings when you get another ulcer

Why is this important?

We know that people who have had an ulcer can feel frustrated and cross if, and when, they get another one. Some people can feel down or stressed at this time and it can be difficult to look after yourself when you are feeling this way. Dealing with your feelings can make you feel better and will make sure you put you and your health first.

The website or booklet will:

- Teach you techniques that have been shown to help improve mood and reduce stress.
- Many people have found these techniques to be helpful.

Card 5 - Optional health professional support:

The website or booklet will also:

- Give people the option to contact a diabetes trained health professional if they wanted to.
- This contact could be in person, over the phone, or by email.

For peer review only

APPENDIX 2: BEHAVIOURAL ANALYSIS OF REDUCE MAINTENANCE INTERVENTION USING THE BEHAVIOUR CHANGE WHEEL (BCW) AND BEHAVIOUR CHANGE TECHNIQUES TAXONOMY (BCTv1)

Key: DFU = Diabetic Foot Ulcer; EO = Barrier emerged from expert opinion; LR = Barrier emerged from literature review; QR = Barrier emerged from qualitative primary research; N/A = theoretical mapping not applicable; * = intervention components and BCTs identified through examination of the BCTv1 and BCW to check for additional intervention functions, target constructs, or behaviour change techniques.

**Barriers/*facilitator* to target behaviour**	**Intervention components**	**Target construct (BCW)**	**Intervention function (BCW)**	**Behaviour Change Technique (using BCTv1)**
**Target behaviour: Engaging in regular foot checking**				
Belief that foot checking will do little to delay getting a DFU [EO; LR]	• Provide a rationale for the necessity of regular foot checking, including evidence that it is effective for delaying DFUs	Psychological capability; Reflective motivation	Education; Persuasion	5.1 Information about health consequences<br>5.6 Information about emotional consequences
	• Provide patient stories demonstrating how regular foot checking helped other patients to take control of their DFUs	Psychological capability; Reflective motivation; Social opportunity	Education; Persuasion; Modelling	5.1 Information about health consequences<br>6.2 Social comparison<br>6.3 Information about others' approval<br>9.1 Credible source
Lack of knowledge regarding DFUs and foot checking procedures/ Lack of confidence in ability to check feet [LR, QR]	• Provide information and pictures on what DFUs look like, what signs of DFUs to look out for, and how to check their feet	Physical capability; Psychological capability	Education; Training	4.1 Instructions on how to perform the behaviour<br>5.1 Information about health consequences
	• Provide an online and printable foot health checklist so patients can spot changes in their foot health<br>• Allow patients to record any changes in their foot health	Psychological capability; Physical opportunity	Training; Environmental structuring	4.1 Instructions on how to perform the behaviour<br>12.5 Adding objects to the environment
	• Provide patient stories demonstrating how easy it was for other patients to engage in regular foot checking	Reflective motivation; Social opportunity	Persuasion; Modelling	6.2 Social comparison<br>6.3 Information about others' approval<br>9.1 Credible source
Forgetting to check your feet [EO, QR]<br><br>*Reminders to check your feet [EO,QR]*	• Allow patients to set up regular reminders to check your feet daily by email or text messages and decide on the frequency of these reminders	Physical opportunity	Environmental structuring	7.1 Prompts/cues

**Barriers/*facilitator* to target behaviour**	**Intervention components**	**Target construct (BCW)**	**Intervention function (BCW)**	**Behaviour Change Technique (using BCTv1)**
Difficulties engaging in foot checking in the long-term [LR, QR]<br><br>*Integrating foot checking into your routine [QR]*	• Allow patients to set their own daily foot checking goals<br>• Encourage patients to make a foot checking action plan	Reflective motivation	Enablement	1.1 Goal setting (behaviour)<br>1.4 Action planning<br>1.5 Review behaviour goal(s)
	• Advise patients to regularly practice foot checking in the same context (e.g. after showering, when putting socks on)	Psychological capability; Automatic motivation	Training; Enablement	8.1 Behavioural practice/rehearsal<br>8.3 Habit formation
Physical limitations, deformities and barriers (e.g. limited mobility, foot deformities, poor eyesight) [LR, QR]<br><br>*Using a mirror [QR]; Getting someone to check your feet for you [QR]*	• Provide guidance on how to check your feet if you have physical limitations, including using a mirror to check the bottom of your feet and asking someone else to check for you<br>• Provide information on the signs of DFUs and foot checking procedures for significant others who are helping with foot checking	Physical capability; Psychological capability; Social opportunity	Training; Education; Enablement	3.2 Social support (practical)<br>4.1 Instructions on how to perform the behaviour
**Target behaviour: Engaging in rapid self-referral in the event of changes in foot health**				
Belief that self-referral will <u>do little</u> to aid DFU healing [EO, LR] /Lack of confidence in DFU team [QR]	• Provide a rationale for the necessity of reporting any signs of foot damage, including evidence that this is effective for DFU healing	Psychological capability; Reflective motivation	Education; Persuasion	5.1 Information about health consequences<br>5.6 Information about emotional consequences
	• Provide patient stories demonstrating how rapid self-referral helped other patients to take control of their DFUs, and how they overcame previous frustrations with the DFU team and feelings that it was not worth it	Psychological capability; Reflective motivation; Social opportunity	Education; Persuasion; Modelling	5.1 Information about health consequences<br>6.2 Social comparison<br>6.3 Information about others' approval<br>9.1 Credible source
Lack of understanding regarding when to seek help and who to contact [EO, QR]	• Provide advice on when you may need to contact your diabetes team<br>• Advise patients to find out the contact details of their DFU team	Psychological capability	Education	4.1 Instructions on how to perform the behaviour

For peer review only - http://bmjopen.bmj.com/site/about/guidelines.xhtml

**Barriers/*facilitator* to target behaviour**	**Intervention components**	**Target construct (BCW)**	**Intervention function (BCW)**	**Behaviour Change Technique (using BCTv1)**
Difficulty accessing the DFU team and getting a quick appointment [EO, QR]	• Provide information on the national guidelines regarding timeline for referrals to DFU team<br>• Invite patients to refer to their local procedure for self-referrals given in their REDUCE action plan in the initiation phase<br>• Provide advice on how to communicate the reason for self-referral when contacting the DFU team	Psychological capability	Education	4.1 Instructions on how to perform the behaviour
Forgetting the contact details for the foot care team [EO]	• Invite patients to record the contact details of their foot care team and print this record to act as a reminder	Physical opportunity	Environmental structuring	7.1 Prompts/cues<br>12.5 Adding objects to the environment
Forgetting to contact their foot care team [EO]	• Invite patients to set up reminders to contact their foot care team if they record any signs of foot damage into the maintenance intervention and decide on the frequency of these reminders	Physical opportunity	Environmental structuring	7.1 Prompts/cues
Concerns about looking foolish or wasting the DFU team's time when reporting changes in foot health that turn out to be normal/ Not wanting to bother the DFU team [EO, QR]	• Reassure patients that health professionals would rather they were contacted early so they are better able to treat the DFU<br>• Provide patient stories on how other patients overcame feelings of being a burden<br>• Provide personalised feedback on whether or not they should self-refer, based on the answers they give to the foot health checklist	Psychological capability; Reflective motivation	Education; Persuasion	4.1 Instructions on how to perform the behaviour<br>6.3 Information about others' approval
**Target behaviour: Engaging in graded and regular physical activity**				
Belief that physical activity will do little to delay getting a DFU [EO, LR]<br><br>*Awareness of non-DFU related benefits of physical activity that are immediate and salient [EO]*	• Provide a rationale for the necessity of graded and regular physical activity and evidence that it is effective for delaying DFUs<br>• Provide a quiz about the benefits of physical activity for delaying DFUs, including other physical and mental benefits (e.g. improved sleep and energy, alleviation from aches and pains)	Psychological capability; Reflective motivation	Education; Persuasion	5.1 Information about health consequences<br>5.6 Information about emotional consequences
	• Provide patient stories demonstrating how graded and regular physical activity helped other patients to take control of their DFUs and led to other salient benefits	Reflective motivation; Social opportunity	Education; Persuasion; Modelling	5.1 Information about health consequences<br>6.2 Social comparison<br>6.3 Information about others' approval<br>9.1 Credible source

For peer review only - http://bmjopen.bmj.com/site/about/guidelines.xhtml

**Barriers/*facilitator* to target behaviour**	**Intervention components**	**Target construct (BCW)**	**Intervention function (BCW)**	**Behaviour Change Technique (using BCTv1)**
Concerns regarding the safety of physical activity [EO, QR]	• Reassure patients that gradual physical activity is safe (e.g. shouldn't cause too much shoe rubbing) and can be done when you do not have a DFU<br>• Address patients' individual physical activity concerns in the initiation phase	Psychological capability	Education	4.1 Instruction on how to perform the behaviour<br>5.1 Information about health consequences
Lack of belief in one's ability to engage in physical activity [LR] /Physical limitations (e.g. arthritis, breathlessness, foot discomfort/pain) [QR]/Bad weather [QR]<br><br>*Finding a suitable activity [QR]*	• Provide patient stories demonstrating how easy it was for other patients to engage in graded and regular physical activity, even though they are at high risk of developing DFUs or have health problems	Reflective motivation; Social opportunity	Persuasion; Modelling	6.2 Social comparison<br>6.3 Information about others' approval<br>9.1 Credible source
	• Provide a variety of examples of safe low-to-moderate physical activity, including activities that are non-weight bearing and can be done in bad weather	Psychological capability	Education	4.1 Instruction on how to perform the behaviour
Difficulties engaging in physical activity in the long-term [LR, QR]<br><br>*Social support [LR]; Provision of pedometers [LR, QR]; Integrating physical activity into your routine [QR]*	• Invite patients to set their own weekly physical activity goals<br>• Invite patients to self-monitor physical activity and provide personalised advice on how to modify goals based on self-monitoring<br>• Invite patients to make a physical activity action plan<br>• Invite patients to set easy-to-perform tasks and make them increasingly more difficult over time*	Reflective motivation; Psychological capability	Enablement; Training	1.1 Goal setting (behaviour)<br>1.4 Action planning<br>1.5 Review behaviour goal(s)<br>2.2 Feedback on behaviour<br>2.3 Self-monitoring of behaviour<br>8.7 Graded tasks*
	• Provide a free pedometer to those who would like one and encourage people to set daily step goals	Reflective motivation; Psychological capability; Physical opportunity	Enablement; Training; Environmental structuring	1.1 Goal setting (behaviour)<br>2.2 Feedback on behaviour<br>2.3 Self-monitoring of behaviour<br>12.5 Adding objects to the environment
	• Suggest that patients ask a friend/relative to exercise with them	Social opportunity	Enablement	3.1 Social support (unspecified)
	• Invite patients to regularly practice physical activity in the same context (e.g. after lunch)	Psychological capability; Automatic motivation	Training; Enablement	8.1 Behavioural practice/rehearsal<br>8.3 Habit formation

For peer review only

**Barriers/*facilitator* to target behaviour**	**Intervention components**	**Target construct (BCW)**	**Intervention function (BCW)**	**Behaviour Change Technique (using BCTv1)**
Forgetting to engage in physical activity [EO]<br><br>*Reminders [EO, LR]*	• Invite patients to set up email reminders to engage in physical activity and decide on the frequency of these reminders	Physical opportunity	Environmental structuring	7.1 Prompts/cues
**Target behaviour: Engaging in emotional management**				
Belief that emotional management will do little to delay getting a DFU or help with difficult emotions [EO; QR]	• Explain the necessity of emotional management for promoting engagement with the other foot care behaviours and provide evidence that they are effective for dealing with difficult emotions	Psychological capability; Reflective motivation	Education; Persuasion	5.1 Information about health consequences<br>5.6 Information about emotional consequences
	• Provide patient stories demonstrating how emotional management techniques helped other patients to take control of their DFUs	Psychological capability; Reflective motivation; Social opportunity	Education; Persuasion; Modelling	5.1 Information about health consequences<br>6.2 Social comparison<br>6.3 Information about others' approval<br>9.1 Credible source
Belief that emotional management is not relevant to them [QR]	• Provide emotional management as an optional part of the intervention<br>• Remind patients about emotional management at times of stress (e.g. if the foot health checklist highlights that they may have signs of getting a DFU)	N/A	N/A	N/A
Belief that the emotional management techniques do not fit with their preferable approach to emotional management [QR]	• Provide a range of techniques that may fit with a patients' preferred approach to emotional management (e.g. cognitive and behavioural techniques)	N/A	N/A	N/A
Lack of understanding regarding how to do the emotional management techniques [EO]	• Provide guidance on how to do the emotional management techniques	Psychological capability	Training	4.1 Instructions on how to perform the behaviour
	• Provide guided audio recordings of emotional management exercises	Physical opportunity	Environmental structuring	12.5 Adding objects to the environment
Lack of confidence in ability to practice emotional management techniques [EO]	• Provide patient stories demonstrating how easy it was for other patients to practice the emotional management techniques	Reflective motivation; Social opportunity	Persuasion; Modelling	6.2 Social comparison<br>6.3 Information about others' approval<br>9.1 Credible source

For peer review only - http://bmjopen.bmj.com/site/about/guidelines.xhtml

Barriers/*facilitator* to target behaviour	Intervention components	Target construct (BCW)	Intervention function (BCW)	Behaviour Change Technique (using BCTv1)
Difficulties engaging in emotional management in the long-term [EO]	• Invite patients to set their own emotional management practice goals	Reflective motivation	Enablement	1.1 Goal setting (behaviour)<br>1.4 Action planning<br>1.5 Review behaviour goal(s)
**Subsidiary behaviour: Engaging with the digital maintenance intervention**				
Low confidence in ability to use digital interventions [LR; QR]<br><br>*Technical support to use digital interventions [EO]*	• Health professionals introduce the digital maintenance intervention in the initiation phase and provide technical support as required<br>• Suggest that family and friends could assist the patient with digital intervention use, if appropriate	Physical capability; Psychological capability; Social opportunity	Training; Education; Enablement	3.2 Social support (practical)<br>4.1 Instructions on how to perform the behaviour
	• Health professionals will speak favourably about digital intervention use and outline its advantages<br>• Build patients' confidence in using the digital intervention by demonstrating how easy it is to use the intervention, and addressing any self-doubts*	Reflective motivation	Education; Persuasion	9.1 Credible source<br>15.1 Verbal persuasion about capability*

For peer review only

For peer review only - http://bmjopen.bmj.com/site/about/guidelines.xhtml

BMJ Open

Intervention planning for the REDUCE maintenance intervention: a digital intervention to reduce re-ulceration risk among patients with a history of diabetic foot ulcers

Journal:	*BMJ Open*
Manuscript ID	bmjopen-2017-019865.R1
Article Type:	Research
Date Submitted by the Author:	11-Jan-2018
Complete List of Authors:	Greenwell, Kate; Centre for Clinical and Community Applications of Health Psychology, University of Southampton<br>Sivyer, Katy; Centre for Clinical and Community Applications of Health Psychology, University of Southampton<br>Vedhara, Kavita; University of Nottingham Division of Primary Care<br>Yardley, Lucy ; Centre for Clinical and Community Applications of Health Psychology, University of Southampton<br>Game, Frances; Derby Teaching Hospitals NHS Foundation Trust<br>Chalder, Trudie; King's College London, Department of Psychological Medicine<br>Richards, Gayle; Northern Devon Healthcare NHS Trust, Department of Diabetes<br>Drake, Nikki; Bristol Community Health<br>Gray, Katie; Derby Teaching Hospitals NHS Foundation Trust<br>Weinman, John; King's College London Institute of Pharmaceutical Science<br>Bradbury, Katherine; Centre for Clinical and Community Applications of Health Psychology, University of Southampton
**Primary Subject Heading**:	Diabetes and endocrinology
Secondary Subject Heading:	Qualitative research, Patient-centred medicine, Research methods
Keywords:	Diabetic foot < DIABETES & ENDOCRINOLOGY, Diabetes & endocrinology < INTERNAL MEDICINE, WOUND MANAGEMENT

SCHOLARONE™
Manuscripts

Intervention planning for the REDUCE maintenance intervention: a digital intervention to reduce re-ulceration risk among patients with a history of diabetic foot ulcers

Kate Greenwell[1]*[a], Katy Sivyer[1]*, Kavita Vedhara[2], Lucy Yardley[1], Frances Game[3], Trudie Chalder[4], Gayle Richards[5], Nikki Drake[6], Katie Gray[3], John Weinman[7], Katherine Bradbury[1]

*Kate Greenwell and Katy Sivyer contributed equally to this paper

[a]Corresponding author: Centre for Clinical and Community Applications of Health Psychology, University of Southampton, Shackleton Building, Highfield Campus, Southampton, SO17 1BJ, UK; Email: reduceproject@soton.ac.uk; Tel: +44 (0) 2380 5950

[1]Centre for Clinical and Community Applications of Health Psychology, University of Southampton, Southampton, UK

[2]Division of Primary Care, School of Medicine, University of Nottingham, Nottingham, UK.

[3]Derby Teaching Hospitals NHS Foundation Trust, Derby, UK

[4]Department of Psychological Medicine, King's College London, London, UK

[5]Department of Diabetes, Northern Devon Healthcare NHS Trust, Barnstaple, UK

[6]Bristol Community Health, Bristol, UK

[7]Institute of Pharmaceutical Science, King's College London, London, UK

Word count: 4701 words

For peer review only - http://bmjopen.bmj.com/site/about/guidelines.xhtml

For peer review only

ABSTRACT

Objectives: To develop a comprehensive intervention plan for the REDUCE maintenance intervention to support people who have had diabetic foot ulcers (DFUs) to sustain behaviours that reduce re-ulceration risk.

Design: Theory-, evidence- and person-based approaches to intervention development were used. In phase 1 of intervention planning, evidence was collated from a scoping review of the literature and qualitative interviews with patients who have had DFUs (N=20). This was used to identify the psychosocial needs and challenges of this population, and barriers and facilitators to the intervention's target behaviours: regular foot checking, rapid self-referral in the event of changes in foot health, graded and regular physical activity, and emotional management. In phase 2, this evidence was combined with expert consultation to develop the intervention plan. Brief 'guiding principles' for shaping intervention development were created. 'Behavioural analysis' and 'logic modelling' were used to map intervention content onto behaviour change theory to comprehensively describe the intervention and its hypothesised mechanisms.

Results: Key challenges to the interventions' target behaviours included patients' uncertainty regarding when to self-refer, physical limitations affecting foot checking and physical activity, and, for some, difficulties managing negative emotions. Important considerations for the intervention design included a need to increase patients' confidence in making a self-referral and in using the maintenance intervention, and a need to acknowledge that some intervention content might be relevant to only some patients (emotional management, physical activity). The behavioural analysis identified the following processes hypothesised to facilitate long-term behaviour maintenance including; increasing patients' skills, self-

For peer review only - http://bmjopen.bmj.com/site/about/guidelines.xhtml

efficacy, knowledge, positive outcome expectancies, sense of personal control, social support, and physical opportunity.

Conclusions: This research provides a transparent description of the intervention planning for the REDUCE maintenance intervention. It provides insights into potential barriers and facilitators to the target behaviours and potentially useful behaviour change techniques to use in clinical practice.

STRENGTHS AND LIMITATIONS OF THIS STUDY

- This research will inform the development of a novel intervention to support the prevention and management of DFUs and is in keeping with recent NICE research priorities for the diabetic foot.
- The integration of theory- evidence- and person-based approaches provided complementary insights into how an intervention could be designed to maximise its acceptability, feasibility, and potential effectiveness.
- The REDUCE maintenance intervention plan is comprehensively described and the intervention's potential mechanisms of actions made explicit, thereby increasing transparency, and facilitating application of this intervention planning methodology by other intervention developers.
- Although the qualitative sample was representative of patients with a DFU (who tend to be older and may therefore be retired), few younger and employed people were recruited so their views remain less well understood.
- Although the rapid scoping review allowed scientific evidence to be quickly incorporated into the intervention plan at an early stage, it was not systematic, so it is possible that some literature may have been missed.

For peer review only - http://bmjopen.bmj.com/site/about/guidelines.xhtml

BACKGROUND

Foot ulceration is a common, chronic, and costly complication of diabetes.[1–3] Healing is slow and recurrence is common, with approximately 40% of patients re-ulcerating within 12 months.[4–6] The physical and emotional burden of ulceration is considerable; 20% of ulcers result in amputation and 32% of patients are depressed, which is associated with a threefold greater risk of mortality.[2,7] Although diabetic foot care has been deemed a priority,[2] treatments to prevent ulceration are based largely on expert opinion and small, underpowered, studies.[2,8] Systematic reviews have found no evidence that education alone improves clinical outcomes.[9–12] However, research suggests that psychosocial and behavioural factors may play a central role in healing and prevention.[13]

Evidence suggests an association between longer delays in help seeking and increased ulcer severity, highlighting the importance of regular foot-checking and rapid self-referral.[14] Although physical activity is generally encouraged in diabetes to promote glycaemic control and reduce cardiovascular risk, there is a common assumption that greater physical activity may increase ulceration risk in people at risk of DFUs. However, research suggests that moderate, regular activity may decrease risk, or at worst, be unrelated to risk.[15,16] Emotional management may also play a role. Following a diabetic foot ulcer (DFU), people may experience difficult emotions, including depression, blame, and guilt.[17] Depression has been associated with greater ulcer incidence and recurrence, and a slower rate of ulcer healing.[18–20] NICE have consequently recommended the development of new interventions targeting such factors.[2]

‘REDUCE’, a novel complex cognitive behavioural intervention,[21] was developed to reduce re-ulceration risk and promote healing by modifying associated psychological and behavioural factors.[22] These factors include; non-adherence to recommended foot care

For peer review only - http://bmjopen.bmj.com/site/about/guidelines.xhtml

procedures (e.g. foot checking), delayed help-seeking for changes in foot health, low or irregular levels of physical activity, and difficulties in managing negative emotions.

REDUCE consists of two phases; an initiation phase of eight weekly sessions with a nurse or podiatrist to start psychological and behavioural change, and a maintenance phase involving two additional sessions held one and three months later to help sustain these changes. A full description of the intervention can be found in Vedhara et al.[22]A feasibility study found REDUCE to be acceptable and feasible for patients and preliminary descriptive findings suggested that patients experienced changes in many of the psychological and behavioural factors targeted by the intervention.[22] However, long-term maintenance of these changes may be more effective if the intervention were available indefinitely, and when patients require it. Low-intensity interventions delivered by websites, smartphones, or a booklet provide a low-cost solution. This paper describes the planning process for an intervention that will replace the face-to-face maintenance sessions of the original intervention.

The key objective of the REDUCE maintenance intervention will be to provide support to people who have had diabetic foot ulcers (DFUs) to increase their ulcer free survival with limbs intact (i.e. the length of time a patient is free from ulcers without having had an amputation). In keeping with recent NICE research priorities, this will be done through behaviour change and emotional management. It will support people to maintain four behaviours targeted in the initiation phase: regular foot checking, rapid self-referral in the event of changes in foot health, graded and regular physical activity, and emotional management.

Published descriptions of complex interventions and their development process are often inadequate, providing readers with little understanding of what the intervention contains, how decisions regarding its development were made, and how the intervention is hypothesised to

For peer review only

work.[12,23–25] This paper presents the full intervention planning process for the REDUCE maintenance intervention as an example of intervention planning methodology and to increase transparency regarding the intervention's content and hypothesised mechanisms of action. This intervention plan will subsequently inform the development of the REDUCE maintenance intervention.

METHODS AND RESULTS

Intervention planning methodology

The intervention planning used theory-, evidence- and person-based approaches.[21,26–28] The person-based approach recommends grounding intervention development in an in-depth understanding of the patient and their psychosocial context, gained through qualitative research.[26] Intervention planning included two phases: collating and analysing evidence; and creating the intervention plan. Phase one includes two elements: a qualitative and quantitative scoping review, and a qualitative interview study. Phase two includes three elements: 1) creating guiding principles; 2) behavioural analysis; and 3) logic modelling.

In phase one, a rapid scoping review of qualitative and quantitative literature was used to examine the behavioural and psychosocial needs, issues, and challenges of people who have had DFUs. This knowledge was combined with insights gained from a qualitative interview study that explored patients' perspectives on key content and design features for the maintenance intervention. These two studies are both person- and evidence-based approaches as they aim to develop an in-depth understanding of the patients' perspective (person-based approach), while identifying, summarising, and incorporating the evidence-base on the barriers and facilitators to the target behaviours (evidence-based approach). The findings of these two studies were given equal weight when creating the intervention plan.

For peer review only - http://bmjopen.bmj.com/site/about/guidelines.xhtml

Additional barriers and facilitators to the intervention's target behaviours and intervention content were identified through consultation with our multidisciplinary project team who had expertise in diabetic foot ulcers, behaviour change, and intervention development. This team included one diabetologist, two diabetes specialist podiatrists, one diabetes specialist nurse, one cognitive behavioural psychotherapist, five health psychologists, and one research psychologist. Expert opinion was gained through iterative consultation at regular teleconferences and feedback on drafts of the intervention plan.

In line with a person-based approach,[26] all sources of evidence (i.e. scoping review, qualitative study results, expert opinion) were brought together in phase two to create 'guiding principles' that outline the intervention design objectives and key intervention features. Theory-based 'behavioural analysis' and 'logic modelling' [25,28,29] were used to map the evidence and intervention content onto behaviour change theory to comprehensively describe the intervention and its potential mechanisms of action.

Collating and analysing evidence

Qualitative and quantitative scoping review

Purpose

To review evidence examining the behavioural and psychosocial needs, issues, and challenges of people who have had DFUs.

Methods

A rapid scoping review of the qualitative and quantitative literature exploring patients' and health professionals' views and experiences of DFUs and their management was undertaken. This was done to ensure that the initial intervention plan was informed by existing evidence from an early stage. A search was undertaken in Web of Science (covering 1970-2017) to ensure coverage of a range of multidisciplinary journals, easily enabling rapid review. This

For peer review only - http://bmjopen.bmj.com/site/about/guidelines.xhtml

search combined the following terms (“diabetic foot ulcer”) AND (“physical activity” OR exercise), (“self-referral” OR “help seeking”), (check AND (foot OR feet)), and (“emotional management” OR “mood management”). It incorporated any published research that included patients who had previously had a diabetic foot ulcer. Findings regarding beliefs around foot care were excluded if they were only relevant to foot care behaviours not targeted in the REDUCE maintenance intervention (e.g. barriers to adherence to prescription footwear). Articles with a biological focus were excluded. Additional literature was identified through expert consultation and article reference lists. Data were extracted on research design, sample size, participants, and key findings. Using thematic analysis, the key findings were organised into themes relating to the psychosocial and behavioural issues, needs, or challenges to be considered during intervention development.

Results

The review identified seven articles and highlighted six themes relating to people’s beliefs around DFUs and the target behaviours, challenges people face when engaging in the target behaviours, difficult emotions people may experience following a DFU, and concerns about digital interventions (Table 1).

For peer review only

For peer review only - http://bmjopen.bmj.com/site/about/guidelines.xhtml

Table 1 Key themes identified from the rapid scoping review of the psychosocial and behavioural issues, needs and challenges of people who have had DFUs

Key themes	Detail from the literature
Lack confidence in foot checking [17,30]	• Some patients were uncertain about what a DFU was or looked like, what signs of DFUs to look out for, and when the DFU was serious enough to seek help from a health professional. Such uncertainties may lead to delays in seeking help.
Feelings of lack of control in preventing DFUs [17,30]	• Some patients felt they had little or no control in preventing further DFUs, as DFUs still occurred even when they were engaging in foot care behaviours.<br>• Some patients believed that they were unable to prevent DFUs.
Difficult emotions following a DFU [17,31–34]	• Some patients were fearful or worried about developing further DFUs, losing limbs through amputation, and the impact a DFU reoccurrence might have on their lives.<br>• Some patients felt down or had low self-esteem because of how the DFUs had negatively affected their everyday lives (e.g. loss of independence, inability to work and provide for the family, lifestyle changes).<br>• Some patients felt a sense of hopelessness, anger, and frustration when DFUs developed despite their attempts to engage in foot care behaviours.<br>• Some patients felt self-blame or guilt for not paying enough attention to their feet, not controlling their diabetes well, not following foot care advice, or not engaging in foot care behaviours, especially in the event of reoccurrence.<br>• Some patients experienced social isolation (e.g. from restricted mobility, lack of employment) or felt a burden to others because they were dependent on them for daily activities (e.g. cooking and driving).<br>• Some patients found it difficult to share their experiences of a DFU with friends and family.<br>• Some podiatrists acknowledged the emotional impact of DFUs on their patients, specifically the presence of anger, depression, anxiety, and frustration.
Maintaining behaviours long-term may be challenging [17]	• Some patients were not confident that they could maintain foot care behaviours in the long-term, with engagement likely to decrease over time.<br>• Some patients were impatient to resume the physical activities they stopped when they had an active DFU, leading them to do too much activity and risk getting another DFU.
Physical limitations impeding foot checking [34,35]	• Some patients and podiatrists reported physical limitations that prevented patients from engaging in foot care behaviours, including joint mobility problems, neuropathy, and visual impairment.
Concerns over using digital interventions [32]	• Some patients felt they did not have the necessary computer skills for internet or computer-based interventions.

For peer review only - http://bmjopen.bmj.com/site/about/guidelines.xhtml

Qualitative interviews

Purpose

To explore the acceptability and feasibility of initial ideas regarding the content and delivery of the maintenance intervention from the perspective of people who have had DFUs; and to identify potential barriers and facilitators to its target behaviours.

Methods

A total of 250 adult (aged 18+ years) patients with diabetes who had previously had a DFU were contacted by letter by their local NHS podiatry service. Participants were excluded if they had a DFU in the previous two weeks. Sixty-six patients (26%) expressed interest in the study, 53 of whom (21% of original mail-out) were eligible to participate. Eligible respondents were purposively sampled to represent a diverse set of ages (range: 45-91 years), genders, and internet use (Table 2). Twenty participants took part in a single semi-structured interview.

Interviews explored participants' views of the target behaviours and potential intervention features, including foot checking reminders, facilities for note-taking, personalised advice about when to self-refer, advice on pacing physical activity, goal setting, provision of free pedometers, and emotional management techniques. Interviews also explored participants' views on possible modes of intervention delivery, including booklet, website, computer tablet, and smartphones, and the value of additional health professional input. Ideas for potential content, intervention features, and delivery modes were shown on prompt cards. Ideas for intervention features (e.g. pedometers) were chosen based on the multidisciplinary team's knowledge of the evidence for the acceptability and effectiveness of these features for changing the target behaviours. Participants were shown an example of an existing diabetes intervention [36] to demonstrate what a website intervention could look like. Interviews were

For peer review only - http://bmjopen.bmj.com/site/about/guidelines.xhtml

piloted with two people who have had DFUs. See Appendix 1 for the interview schedule and prompt cards.

Interviews were carried out by KG and KS and took place at participants' homes (N=18) or the university (N=2). Participants were reimbursed for travel and given a £10 voucher. All interviews were recorded and transcribed. KG and KS used thematic analysis to identify potential barriers and facilitators to engaging with the target behaviours, and positive and negative perceptions of the potential intervention features and delivery modes. Ethical approval for this study was gained from North West – Greater Manchester West Research Ethics Committee (17/NW/0024).

Table 2 Demographics of patients taking part in the qualitative interviews

Sample Characteristics	Statistics
**Basic Demographics**	**Mean (SD)**
Age	68.30 (11.54)
**Basic Demographics**	**N (%)**
Male	11 (55%)
Marital Status	
Married	7 (35%)
Single	6 (30%)
Widowed	4 (20%)
Divorced	3 (15%)
Employment Status	
Retired	15 (75%)
Redundant due to illness	3 (15%)
Housewife/husband	1 (5%)
Full-time employed	1 (5%)
Educational Status	
Secondary School	10 (50%)
College / Sixth Form / Professional Qualification	7 (35%)
Undergraduate	3 (15%)
**DFU History**	**Mean (SD)**
Years since first DFU (approx.)	6.81 (7.96)
Number of DFUs (approx.)	4.18 (3.86)
Months since last DFU (approx.)	14.65 (11.26)
Duration of last DFU in days (approx.)	298 (400.82)
**Internet Use**	**N (%)**
Access to internet at home	15 (75%)
Access to internet on tablet	7 (35%)
Access to internet on phone	3 (15%)
Frequency of access	
Never	3 (15%)
Less than once a month	3 (15%)
Once a week	1 (5%)

For peer review only - http://bmjopen.bmj.com/site/about/guidelines.xhtml

A few times a week	2 (10%)
Once a day	3 (15%)
Several times a day	8 (40%)

Results

The key findings are outlined below. Example quotes are in Table 3.

Regular foot checking: Generally, participants perceived foot checking as acceptable and important for preventing DFUs. Many found foot checking easy to do and already checked their feet regularly. However, many participants reported physical limitations (e.g. limited mobility) and other physical barriers (e.g. wearing casts or bandages) that restricted foot checking. While some people found it easy to spot changes in foot health, others reported difficulties knowing what to look for and in judging whether any changes were problematic. A few described how it is easy to become lax over time, forgetting to check feet regularly or not thoroughly checking. Participants identified several facilitators to foot checking, including using a mirror to check feet, getting someone else to check, and integrating foot checking into everyday routine (e.g. when putting on socks).

When discussing the planned intervention features (e.g. foot checking reminders, facilities for note-taking), some people believed it would be useful to set up regular email foot checking reminders because it is easy to forget. Others felt reminders could be irritating or were unnecessary, as they, or their podiatrist, already regularly checked their feet. Generally, people thought it would be helpful to be able to make a note of any changes in their foot health to track changes in foot health over time. A few people felt this was unnecessary because they already checked their feet regularly, and knew what to look for, or believed it would be difficult to remember to note down changes.

Table 3 Table of key issues arising from our qualitative study and illustrative quotes

Issue arising from our qualitative study	Participant quotes
Foot checking	

For peer review only - http://bmjopen.bmj.com/site/about/guidelines.xhtml

Some participants had physical limitations that make it difficult to check their feet.	"As you get older you're not so mobile so you can't see right underneath [your foot], so it's a bit of guesswork until you do go…to [the] podiatrist" (P10, Male)
Some people found it difficult to know what to look for when foot checking and when to self-refer.	"Recognising them [DFUs] I think is the hardest part" (P14, Male)<br>"Sometimes…I go [to the podiatrist] and it's not an ulcer…but I can't tell" (P8, Male)
A few participants found it difficult to keep up foot checking long-term.	"You kind of become rather lax about perhaps doing it [foot checking] properly" (P1, Male)
There were mixed views on foot checking reminders.	"I don't think I would need to be reminded. I'm doing it [foot checking] already, really" (P3, Female)<br>"It's nice to have a reminder. Sometimes you get a bit complacent and you think 'Oh, I'll do it next time'" (P10, Male)
Rapid self-referral	
Some participants found it difficult to contact and get an appointment with their DFU team.	"Sometimes you can't get appointments…By the time you are seeing somebody it's either through A&E, because you've been rushed in 'cause your foot's swollen up and changed colour" (P18, Female)
Some participants expressed concerns about self-referring.	"If you do that [point out changes in foot health] every visit and it's nothing to worry about, you're paranoid, micromanaging. But if you don't mention something you've seen previously, you're complacent and don't care about your health. You can't win" (P18, Female)
Some participants found it difficult to know which health professional to contact when reporting DFUs.	"Who do you contact if you have a problem? Your own doctor? Or the nurse, diabetic nurse? Or the podiatrist?" (P5, Male)
Physical activity	
Some participants have physical limitations that make it difficult to engage in physical activity.	"I get very breathless. I don't walk much at all. I know I should, but I don't" (P3, Female)
Some participants also expressed concerns about physical activity causing another DFU.	"Even though you might not have an ulcer, even if you go back to minimal activity…you can still get that ulcer come back" (P18, Female)
Some participants found it can be difficult to keep up with physical activity over time.	"It is easy to find something else to do [instead of physical activity]. You've got to be pretty disciplined" (P6, Female)
There were mixed views on pedometers.	"The pedometer is a really good idea though…It's like a game – you want to make sure you can get as many steps in" (P20, Female)<br>"[The pedometer is] almost like being spied on" (P14, Male)
Emotional management	
Emotional management was relevant and valued by some participants, but not everyone.	"I'm one o' these anxiety merchants, me. I worry for the world…so it'd [emotional management] be very helpful" (P10, Male)<br>"I don't think personally I would have taken it [emotional management] on board at all…it's not gonna make any difference to me…I just think I've got it [DFUs], I've got to put up with it…I don't want to sit on a couch breathing in and out, I want to get on and do something" (P2, Female)
Delivery methods	
Participants were positive about the	"Personally think the website would be far better than the

For peer review only - http://bmjopen.bmj.com/site/about/guidelines.xhtml

idea of a website, but there were some concerns about computer literacy.	booklet…It's prodding me to do it [use the intervention]…If it's in a leaflet, it just gets left" (P14, Male, internet user)<br>"I love…anything interactive like that [the quiz in the example website] I think is great…you feel part of it [the intervention], rather than just being dictated to…[the information] tends to sink in better" (P20, Female, internet user)<br>"If I was competent…I would do it on the computer. But I'm not competent" (P8, Male, infrequent internet user)
A booklet might be helpful for quick reference and for those who do not use the internet.	"A booklet is always there, you can always refer to it, you've got something in black and white" (P8, Male)
Delivering the intervention via smartphone was less acceptable.	"Mobile phone - you've got all the problems of the computer, but on a smaller screen…a lot of diabetics [have] got problems with their eyes as well" (P17, Male)
Participants liked the idea of additional health professional support, but not for the intended purpose of supporting behaviour maintenance.	"It'd [additional health professional support] give me the confidence to know that 'well, I am alright with my foot as it is'…because you can get a bit paranoid over it [your foot health]" (P17, Male)<br>"They could give…one-to-one advice on…is there anything else that you could do…better than what I'm doing myself" (P3, Female)

Rapid self-referral in the event of changes in foot health: Most participants were positive about self-referral, viewing it as important. However, many people found it difficult to contact their DFU team. Long waiting times left some participants worried about how their foot health might decline in the meantime, which led one person to treat their feet themselves, instead of self-referring. In contrast, some participants reported the opposite and found it easy to get an appointment with their DFU team. A few participants were unsure which health professional to contact when reporting DFUs (e.g. podiatrist, diabetes nurse, GP). Some expressed concerns about looking foolish or wasting health professionals' time when self-referring for changes in foot health that turned out to be normal. One person had trouble with getting her concerns taken seriously and a few people worried about being a burden to health professionals. Some participants wanted reassurance from health professionals that it was right to have sought help.

Graded and regular physical activity: Most participants were positive about physical activity, stating that they would like to or were already doing it. People generally viewed

For peer review only

For peer review only - http://bmjopen.bmj.com/site/about/guidelines.xhtml

physical activity as important for general health and diabetes management. However, many participants reported physical limitations (e.g. pain, fatigue) or diabetic complications (e.g. neuropathy, residual damage to feet from previous DFUs) that made it difficult to be active. Participants reported that it was important to find the right activity to overcome their physical limitations, suggesting activities that did not put pressure on their feet, such as seated exercises. Some were concerned that physical activity might cause another DFU or exacerbate other health conditions.

Some participants stated that it could be difficult to maintain physical activity. A few mentioned that integrating physical activity into their daily routine (e.g. getting off the bus one stop early) and positive encouragement helped. Participants viewed self-monitoring, goal setting, and pedometers as helpful for maintaining motivation. However, some people disliked the idea of being 'spied on' or told what to do, expressed doubts about the accuracy of pedometers, or were unsure whether they would use them.

Emotional management: Over half of participants viewed emotional management positively and reported experiencing low mood, frustration, anger, and stress either during or after a DFU. Others had not experienced such emotions relating to their DFUs and viewed emotional management as irrelevant. A few people viewed emotional management negatively due to previous negative experiences. For example, some had experienced unhelpful reactions from doctors when discussing emotions, disliked talking about their feelings in counselling, or had received unhelpful information about emotional management (e.g. being given advice that did not consider their physical limitations). Some expressed a lack of understanding about how the emotional management would help or perceived it as contrary to their personal style of managing emotions (i.e. ignoring their problems, 'getting on with it').

For peer review only - http://bmjopen.bmj.com/site/about/guidelines.xhtml

Intervention delivery methods: Most participants were positive about the idea of the intervention being delivered via a booklet. Booklets were perceived as quick and easy to refer to, portable, and easily shared or distributed (e.g. with relatives or picked up from clinics). However, some participants commented that booklets were easily misplaced or forgotten. Most internet users reacted positively to the idea of a website, mainly because it was easy to access, convenient, and had interactive features (e.g. quizzes, email reminders). Nonetheless, non-users and a few infrequent internet users expressed concern about their own computer literacy. Some participants disliked reading on a computer screen and a few participants had concerns about security of web interventions. However, when participants were shown the example website, they generally viewed it positively, stating that it looked easy to use. A few participants would have liked to access the intervention using a computer tablet as they already used one or knew people who did. Most viewed delivery using a smartphone negatively because of their limited use of phones or difficulties with using small screens due to poor eyesight (caused by diabetes). A few participants commented that it might be helpful to deliver the intervention through multiple modes (booklet, website, tablet, or phone).

Generally, participants were in favour of additional health professional support. However, they interpreted this as support to gain reassurance about the status of their foot health, and advice on foot care or when to self-refer (which would be covered in the website/booklet), rather than support to raise motivation for engaging with the target behaviours. Very few participants said they might use this support to answer questions about information in the booklet or website.

Explanations of how the evidence from the scoping review and qualitative study informed intervention planning are provided in the next sections on Guiding Principles and Behavioural Analysis.

For peer review only - http://bmjopen.bmj.com/site/about/guidelines.xhtml

Creating the intervention plan

Creating guiding principles

Purpose

In line with the person-based approach,[26] brief guiding principles are developed and consulted throughout intervention development to ensure that the intervention is underpinned by a coherent focus.

Methods

Drawing upon the findings from our scoping review and qualitative study, key characteristics of target users and the key behavioural issues, needs and challenges the intervention must address were described. From this, guiding principles were created, which outline the intervention design objectives that will address these key behavioural issues, needs and challenges, and the key intervention features designed to achieve these objectives. The multidisciplinary team decided on the key features based on their ability to address the intervention objectives.

Results

People who have had DFUs can feel they have little or no control over preventing DFUs, as DFUs can occur even when people are engaging in foot care behaviours. This leaves people feeling hopeless and frustrated.[17] Some people may feel self-blame or guilt for not engaging in foot care behaviours, especially in the event of reoccurrence.[17] Therefore, one design objective was to reduce feelings of hopelessness, frustration, self-blame, and guilt following a DFU.

People may be uncertain about the signs of a DFU and when to seek help from a health professional.[30] Our qualitative study highlighted that some people were concerned about

For peer review only - http://bmjopen.bmj.com/site/about/guidelines.xhtml

looking foolish, being a burden, or wasting healthcare professionals' time if changes in their feet turn out to be normal. This may delay help seeking. Therefore, one design objective was to build patients' confidence in making a self-referral.

This population are likely to have physical limitations and/or co-morbidities. Our qualitative study highlighted that these challenges may make it difficult for people to engage in foot checking and physical activity. They may also be reluctant to increase activity in case it causes re-ulceration. Thus, one design objective was to acknowledge that patients may have physical limitations that make it difficult to engage in foot checking and physical activity.

Our scoping review highlighted that people may experience difficult emotions following a DFU.[17,31–34] However, some participants in our qualitative research did not experience such emotions and, therefore, did not perceive emotional management as useful. Therefore, one design objective was to acknowledge that emotional management may not be relevant for all patients.

As the physical activity and emotional management content was not relevant to all patients, these components were made optional, rather than mandatory, to avoid discouraging patients from engaging in the other target behaviours if they do not want to increase physical activity or engage in emotional management.

In our qualitative study, many reacted positively to the idea of a web-based intervention, but some participants expressed concerns about their computer literacy. These concerns were also evident in the literature.[32] Therefore, one design objective was to ensure people feel confident in using the maintenance intervention. We decided to deliver the intervention using a website and provide key information and advice in a booklet for quick reference and for non-internet users. At the preceding initiation phase, health professionals will address

For peer review only - http://bmjopen.bmj.com/site/about/guidelines.xhtml

concerns, and speak favourably of the digital intervention to encourage use. Table 4 details the REDUCE maintenance intervention guiding principles.

For peer review only

For peer review only - http://bmjopen.bmj.com/site/about/guidelines.xhtml

Table 4 The guiding principles for the development of the REDUCE maintenance intervention

Intervention design objectives	Key features
To reduce feelings of hopelessness, frustration, self-blame, and guilt following a DFU	• Emphasise target behaviours that patients can engage in to reduce their chances of getting another DFU, while acknowledging that there are precipitating factors (e.g. increased age, neuropathy, foot shape) that are out of their control.<br>• Enhance patients' confidence in the target behaviours (e.g. by providing a rationale for the necessity of the target behaviours, scientific evidence that behaviours are effective, patient stories, and a quiz on the benefits of the behaviours).<br>• Validate patients' feelings of frustration and hopelessness if a DFU does reoccur and avoid arguments that may be viewed as blaming patients for this re-occurrence.<br>• Provide links to emotional management techniques that can help people to manage difficult emotions.
To build patients' confidence in making a self-referral	• Provide links to foot checking training (e.g. by providing information and photographs on what DFUs look like, what signs to look out for, and how often feet should be checked with guided practice).<br>• Provide reassurance that self-referral is necessary (e.g. through a foot health checklist that provides personalised feedback on whether or not patients should self-refer, based on their symptoms).<br>• Address concerns around looking foolish or wasting the DFU team's time when self-referring (e.g. a) emphasise that the DFU team would rather they were contacted early so they are better able to treat any DFUs, b) provide patient stories about how other patients overcame feelings of burden).
To acknowledge that patients may have physical limitations that make it difficult to engage in foot checking and physical activity	• Provide guidance on how to check your feet if you have physical limitations, including using a mirror to check the bottom of your feet and asking someone else to check for you.<br>• Make intervention content on physical activity optional.<br>• Provide guidance about a variety of safe and low impact physical activities to enable patients to find an activity that is suitable for them.<br>• Address physical activity concerns all the way through the intervention (i.e. in the maintenance intervention and prior initiation phase) (e.g. by providing information about the safety of physical activity, patient stories about how other patients overcame these barriers).
To acknowledge that emotional management may not be relevant for all patients	• Make intervention content on emotional management optional.<br>• Emphasise that some people, but not everyone, might experience difficult emotions following a DFU to avoid excluding those who may not relate to this content.<br>• Provide a variety of brief emotional management techniques (e.g. CBT, mindfulness techniques) to allow each person to find a technique that fits with their own personal style of managing emotions.
To ensure patients feel confident in using the maintenance intervention	• Keep website navigation simple and follow guidelines for maximising website usability.<br>• Health professionals at the prior initiation phase will provide technical support, address self-doubts, and

For peer review only - http://bmjopen.bmj.com/site/about/guidelines.xhtml

Intervention design objectives	Key features
	speak favourably of the digital intervention to encourage use.<br>• Encourage friends and family to assist people with website use, if appropriate.<br>• Provide a booklet for quick reference and for those who do not have access to the internet.

For peer review only

For peer review only - http://bmjopen.bmj.com/site/about/guidelines.xhtml

Behavioural analysis

Purpose

To use behaviour change theory to systematically describe the maintenance intervention content, identify potential determinants of behaviour (i.e. what needs to change for a behaviour to occur), and map it onto the evidence derived from our scoping review, our qualitative study, and expert consultation.

Methods

Behavioural analysis involves comprehensively mapping out the elements of an intervention, linking the evidence-base to behaviour change theory and the intervention components. Providing a clear description of the intervention is essential for replication in research and practice, data extraction in systematic reviews, and process evaluation planning.[21,24,25] The Behaviour Change Wheel (BCW [37,38]) and Behaviour Change Techniques Taxonomy (BCTv1 [39]) were developed to standardise the classification and description of complex interventions and help identify an intervention's 'active ingredients' and behavioural determinants. Such standardisation provides a common language to avoid any confusion that may occur when different terminology are used for the same intervention technique or different techniques are referred to using the same terminology.[40] The BCW draws upon the COM-B model, which argues that behaviour is influenced by an individual's Capability, Opportunity, and Motivation to change behaviour.[38]

In addition to the four target behaviours identified from the outset, the behavioural analysis also identified one subsidiary behaviour (engaging with the digital MI) that is necessary to enact these target behaviours. Barriers and facilitators for each behaviour were identified from the primary qualitative research, scoping review, and expert opinion from the multidisciplinary project team. Intervention components that addressed each barrier and facilitator were selected.

For peer review only - http://bmjopen.bmj.com/site/about/guidelines.xhtml

These components are reported using patient-centred, autonomy-supportive language to emphasise the importance of delivering these components in a way that will enhance intrinsic motivation and ensure a positive intervention experience.[26] The intervention components were coded using the BCTv1 and mapped onto the BCW to identify their corresponding intervention function (ways an intervention can change behaviour, e.g. 'education'), and target construct (what needs to change for the behaviour to occur, e.g. 'psychological capability'). The BCTv1 and BCW were then examined to check for potentially useful additional intervention functions, target constructs, or behaviour change techniques.

Results

The behavioural analysis is presented in Appendix 2. The maintenance intervention will target all six behavioural sources included in the BCW (physical and psychological capability, reflective and automatic motivation, and physical and social opportunity), and employ six different BCW intervention functions (education, persuasion, modelling, training, enablement, environmental restructuring) using 18 different BCTs. Intervention components that received a mixed reaction from our qualitative research participants (i.e. foot checking reminders, pedometers) were made optional to promote patient autonomy.

Although participants would have liked additional health professional support, the support participants wanted was more clinical in nature (e.g. advice about foot health or when to self-refer). As such support would be provided in the website/booklet, this form of health professional support was deemed superfluous. Therefore, additional health professional support was not included in the intervention plan. One issue that arose from our qualitative study could only be addressed to a limited degree by the maintenance intervention, namely the difficulties people experienced contacting, and getting an appointment, with their DFU team. This will be addressed by educating patients about the national guidelines and local procedures for self-referrals, and how to communicate the reason for self-referral to their

For peer review only - http://bmjopen.bmj.com/site/about/guidelines.xhtml

DFU team. However, improving local self-referral pathways or modifying health professionals' behaviour is outside of the scope of this intervention.

Logic modelling

Purpose

To model the hypothesised mechanisms of action of the maintenance intervention (i.e. how it is thought to work).[25,28,29]

Methods

The logic model draws together findings from the scoping review, qualitative study, and behavioural analysis into a testable model that outlines how the different intervention components are hypothesised to impact on subsequent components and ultimately affect outcomes.

Results

The logic model (Figure 1) can be broken down into three major components.

Intervention techniques and processes: The intervention techniques summarise the behaviour change techniques outlined in the behavioural analysis and the seven processes they are hypothesised to affect: skills, self-efficacy, knowledge, positive outcome expectancies, sense of personal control, social support and physical opportunity. These are the psychosocial factors that need to be modified for the intervention's target behaviours to change and were identified through the behavioural analysis.

Each set of intervention techniques is hypothesised to mainly affect one of these processes, which subsequently affect one or more of the intervention's target behaviours. They are organised in order of importance, with more integral processes that were consistently

For peer review only - http://bmjopen.bmj.com/site/about/guidelines.xhtml

identified as key in the scoping review and qualitative study at the top and less integral processes at the bottom (e.g. optional features).

Purported mediators: Purported mediators are the target behaviours of the intervention that are hypothesised to directly affect DFUs in the long-term. These behaviours are divided into ‘core behaviours’ that are hypothesised to be most important in determining DFU outcomes (foot checking, rapid self-referral), and ‘optional behaviours’ that are only relevant for some patients (physical activity, emotional management). These behaviours’ may impact either directly, as in the case of physical activity, or indirectly, via their effect on the other target behaviours, as is the case in emotional management. Emotional management is hypothesised to have an indirect effect on the other behaviours due to the negative effects that low mood (or negative thoughts) can have on behavioural engagement.

Outcomes: The logic model specifies three outcomes that the intervention is ultimately trying to change, the primary outcome of interest (ulcer free survival with limbs intact), and two interim outcomes that may be affected by the target behaviours and may, directly or indirectly, affect the primary outcome (severity of DFU at presentation and time taken for DFU healing in the event of a recurrence).

DISCUSSION

This paper describes the use of theory-, evidence- and person-based approaches [28] to developing an intervention plan for the REDUCE maintenance intervention, an intervention that aims to reduce re-ulceration risk by supporting patients to maintain behaviour change and emotional management. These different approaches provided complementary insights into how the intervention could be designed to maximise its acceptability, feasibility, and effectiveness. For example, the scoping review highlighted that patients experience difficult emotions following DFUs,[17,31–34], however, the qualitative interviews suggested that this

For peer review only - http://bmjopen.bmj.com/site/about/guidelines.xhtml

was only relevant for some patients, suggesting that this content should be made optional. In line with person- and evidence-based approaches, our scoping review and qualitative study deepened our understanding of the psychological and behavioural needs of people who have had DFUs and highlighted several barriers and facilitators to the intervention's target behaviours, some of which had been highlighted in the literature (e.g. lack of knowledge regarding what to look for when foot checking [17,30]) and some which had received little prior attention (e.g. lack of knowledge about when to self-refer). It also highlighted important advantages of, and barriers to, successful use of different intervention delivery methods (e.g. lack of confidence in ability to use digital interventions). Our qualitative study updated prior research published over a decade ago that highlighted concerns regarding limited computer access and poor computer skills among people at risk of DFUs.[32] Our guiding principles succinctly summarised the distinctive design objectives and features of the maintenance intervention, while our behavioural analysis and logic modelling comprehensively described the intervention and its potential mechanisms of action.

This is the first paper to use this methodology to provide a comprehensive plan of a DFU intervention. Transparent reporting of the intervention planning process will allow other researchers to easily understand how this methodology could be applied to different intervention contexts and facilitate comparison between different interventions.[12,23–25] The use of primary qualitative research allowed us to understand patients' views on the delivery methods for behaviour change interventions and three behaviours that have received little attention in the DFU literature to date: engaging in rapid self-referral, graded and regular physical activity, and emotional management. For example, participants had mixed reactions to some behaviours (i.e. physical activity and emotional management) and design features (e.g. email reminders), which were subsequently made optional. Participants also reported experiencing difficulties with accessing their DFU team when self-referring. Future

For peer review only - http://bmjopen.bmj.com/site/about/guidelines.xhtml

research should further explore and address any professional and organisational barriers to self-referral.

The qualitative research used purposive sampling which enabled us to explore the acceptability and feasibility of a digital intervention across a diverse set of people, including those who were frequent and infrequent internet users. Although the sample was representative of the population of people with DFUs (who tend to be older [14] and may therefore be retired), it would be helpful to explore the views of younger and employed people, as they may report different barriers to behaviour change. The rapid scoping review allowed scientific evidence to be quickly incorporated into the intervention plan, but it was not systematic, so it is possible that some literature was missed.

Recent NICE guidelines for the prevention and management of diabetic foot problems [2] identified a need to develop and evaluate new interventions targeting psychological and behavioural factors. Our research has provided a plan for such an intervention, as well as identified potential barriers to behaviour change and behaviour change techniques that are likely to be useful within clinical practice. In future work, we intend to use this intervention plan to develop the maintenance intervention and then conduct an effectiveness trial to evaluate the effectiveness and cost-effectiveness of the entire REDUCE intervention, whilst also examining if the intervention works as hypothesised.

ACKNOWLEDGMENTS

The research team acknowledges the support of the National Institute of Health Research Clinical Research Network (NIHR CRN). We are grateful to the members of the REDUCE PPI panel for their valuable input on the study materials and interview schedule, and the participants who took part in this research.

COMPETING INTERESTS

For peer review only - http://bmjopen.bmj.com/site/about/guidelines.xhtml

All authors have completed the ICMJE uniform disclosure form at www.icmje.org/coi_disclosure.pdf and declare: KG, KS, KV, LY, FG, TC, ND, GR and KB had financial support from NIHR for the submitted work; TC had other support from NIHR; no financial relationships with any organisations that might have an interest in the submitted work in the previous three years; and no other relationships or activities that could appear to have influenced the submitted work.

FUNDING

This article summarises independent research funded by the National Institute for Health Research (NIHR) under its Programme Development Grants Programme (Grant Reference Number RP-DG-0615-10005). The views expressed are those of the author(s) and not necessarily those of the NHS, the NIHR or the Department of Health.

CONTRIBUTIONS

KGre, KS, KV, LY, FG, TC, GR, ND, KGra, JW and KB designed the study. KG, KS, KB, and LY led the intervention planning, with input from the other co-authors. KGre and KS were responsible for recruitment, carrying out the interviews, and analysing the data, with support from KB. KGre and KS jointly drafted the manuscript with initial support from KB and LY. KGre, KS, KV, LY, FG, TC, GR, ND, KGra, JW and KB critically reviewed the manuscript, contributing important intellectual content, and approved the final manuscript.

DATA SHARING STATEMENT

No additional data are available.

REFERENCES

1 Singh N, Armstrong DG, Lipsky BA. Preventing foot ulcers. *J Am Med Assoc* 2005;293:217–28. doi:10.1001/jama.293.2.217

For peer review only - http://bmjopen.bmj.com/site/about/guidelines.xhtml

2 NICE. Diabetic foot problems: prevention and management. 2015

3 Kerr M. Improving footcare for people with diabetes and saving money: an economic study in England. 2017

4 Margolis D, Kantor J, Berlin J. Healing of diabetic neuropathic foot ulcers receiving standard treatment. *Diabetes Care* 1999;22:692–5.

5 Boulton AJM. The diabetic foot: Grand overview, epidemiology and pathogenesis. *Diabetes Metab Res Rev* 2008;24:S3–6. doi:10.1002/dmrr.833

6 Armstrong DG, Boulton AJM, Bus SA. Diabetic foot ulcers and their recurrence. *N Engl J Med* 2017;376:2367–75. doi:10.1056/NEJMra1615439

7 Ismail K, Winkley K, Stahl D, *et al.* A cohort study of people with diabetes and their first foot ulcer. *Diabetes Care* 2007;30:1473–9.

8 van Netten JJ, Price PE, Lavery LA, *et al.* Prevention of foot ulcers in the at-risk patient with diabetes: a systematic review. *Diabetes Metab Res Rev* 2016;32:84–98. doi:10.1002/dmrr.2701

9 Mason J, O'Keeffe C, McIntosh A, *et al.* A systematic review of foot ulcer in patients with Type 2 diabetes mellitus. I: prevention. *Diabet Med* 1999;16:801–12. doi:10.1046/j.1464-5491.1999.00133.x

10 Dorresteijn J, Kriegsman D, Assendelft W, *et al.* Patient Education for Preventing Diabetic Foot Ulceration. *Cochrane Database Syst Rev* Published Online First: 2012. doi:10.1002/14651858.CD001488.pub2

11 Hunt DL. Diabetes: foot ulcers and amputations. *BMJ Clin Evid* 2011;2011:1–44.

12 Hoogeveen RC, Dorresteijn JA, Kriegsman DM, *et al.* Complex interventions for

For peer review only

For peer review only - http://bmjopen.bmj.com/site/about/guidelines.xhtml

preventing diabetic foot ulceration. *Cochrane Database Syst Rev* 2015;8:1–42. doi:10.1002/14651858.CD007610.pub3

13 Vileikyte L. Psychosocial and behavioral aspects of diabetic foot lesions. *Curr Diab Rep* 2008;8:119–25. doi:10.1007/s11892-008-0022-1

14 Clincal Audit and Registries Management Service. National diabetes foot care audit report 2014-2015. 2016 doi:9781783866762

15 Armstrong DG, Lavery LA, Holtz-Neiderer K, *et al.* Variability in activity may precede diabetic foot ulceration. *Diabetes Care* 2004;27:1980–4.

16 Lemaster JW, Mueller MJ, Reiber GE, *et al.* Effect of weight-bearing activity on foot ulcer incidence in people with diabetic peripheral neuropathy: feet first randomized controlled trial. *Phys Ther* 2008;88:1385–98. doi:10.2522/ptj.20080019

17 Beattie AM, Campbell R, Vedhara K. 'What ever I do it's a lost cause.' The emotional and behavioural experiences of individuals who are ulcer free living with the threat of developing further diabetic foot ulcers: a qualitative interview study. *Heal Expect* 2014;17:429–39. doi:10.1111/j.1369-7625.2012.00768.x

18 Winkley K, Stahl D, Chalder T, *et al.* Risk factors associated with adverse outcomes in a population-based prospective cohort study of people with their first diabetic foot ulcer. *J Diabetes Complications* 2007;21:341–9. doi:10.1016/j.jdiacomp.2007.09.004

19 Monami M, Longo R, Desideri CM, *et al.* The diabetic person beyond a foot ulcer: healing, recurrence, and depressive symptoms. *J Am Podiatr Med Assoc* 2008;98:130–6. doi:98/2/130 [pii]

20 Vedhara K, Miles JN V, Wetherell MA, *et al.* Coping style and depression influence the healing of diabetic foot ulcers: observational and mechanistic evidence.

For peer review only - http://bmjopen.bmj.com/site/about/guidelines.xhtml

Diabetologia 2010;53:1590–8. doi:10.1007/s00125-010-1743-7

21 Craig P, Dieppe P, Macintyre S, *et al.* Developing and evaluating complex interventions: New guidance. 2008.

22 Vedhara K, Beattie A, Metcalfe C, *et al.* Development and preliminary evaluation of a psychosocial intervention for modifying psychosocial risk factors associated with foot re-ulceration in diabetes. *Behav Res Ther* 2012;50:323–32. doi:10.1016/j.brat.2012.02.013

23 Michie S, Abraham C. Advancing the science of behaviour change: a plea for scientific reporting. *Addiction* 2008;103:1409–10. doi:10.1111/j.1360-0443.2008.02291.x

24 Michie S, Abraham C. Interventions to change health behaviours: evidence-based or evidence-inspired? *Psychol Health* 2004;19:29–49. doi:10.1080/0887044031000141199

25 Moore G, Audrey S, Barker M, *et al.* Process evaluation of complex interventions. UK Medical Research Council (MRC) guidance. 2012.

26 Yardley L, Morrison L, Bradbury K, *et al.* The person-based approach to intervention development: application to digital health-related behavior change interventions. *J Med Internet Res* 2015;17:e30. doi:10.2196/jmir.4055

27 Kok G, Schaalma H. Using theory in psychological interventions. In: Michie S, Abraham C, eds. Health Psychology in Practice. 2004:203–29. doi:10.1002/9780470694008.ch11

28 Band R, Bradbury K, Morton K, *et al.* Intervention planning for a digital intervention for self-management of hypertension: a theory-, evidence- and person-based approach.

For peer review only - http://bmjopen.bmj.com/site/about/guidelines.xhtml

Implement Sci 2017;12:25. doi:10.1186/s13012-017-0553-4

29 Baxter SK, Blank L, Woods HB, *et al.* Using logic model methods in systematic review synthesis: describing complex pathways in referral management interventions. *BMC Med Res Methodol* 2014;14:62. doi:10.1186/1471-2288-14-62

30 Searle A, Gale L, Campbell R, *et al.* Reducing the burden of chronic wounds: prevention and management of the diabetic foot in the context of clinical guidelines. *J Health Serv Res Policy* 2008;13 Suppl 3:82–91. doi:10.1258/jhsrp.2008.008011

31 Hjelm K, Nyberg P, Apelqvist J. Gender influences beliefs about health and illness in diabetic subjects with severe foot lesions. *J Adv Nurs* 2002;40:673–84. doi:10.1046/j.1365-2648.2002.02427.x

32 Burnside J, Wells W, Smith M, *et al.* Education for the prevention of foot ulcers: views of those with neuropathy. 2006.

33 Kinmond K, McGee P, Gough S, *et al.* 'Loss of self': a psychosocial study of the quality of life of adults with diabetic ulceration. *World Wide Wounds* 2003;13:6–16. doi:10.1016/S0965-206X(03)80025-6

34 Searle A, Campbell R, Tallon D, *et al.* A qualitative approach to understanding the experience of ulceration and healing in the diabetic foot: Patient and podiatrist perspective. *Wounds* 2005;66:1–12.

35 Pollock RD, Unwin NC, Connolly V. Knowledge and practice of foot care in people with diabetes. *Diabetes Res Clin Pract* 2004;64:117–22. doi:10.1016/j.diabres.2003.10.014

36 Rowsell A, Muller I, Murray E, *et al.* Views of people with high and low levels of health literacy about a digital intervention to promote physical activity for diabetes: A

For peer review only

For peer review only - http://bmjopen.bmj.com/site/about/guidelines.xhtml

qualitative study in five countries. *J Med Internet Res* 2015;17:e230. doi:10.2196/jmir.4999

37 Michie S, Atkins L, West R. *The Behaviour Change Wheel: A Guide to Designing Interventions*. London: Silverback Publishing 2014

38 Michie S, van Stralen MM, West R. The behaviour change wheel: a new method for characterising and designing behaviour change interventions. *Implement Sci* 2011;6:42. doi:10.1186/1748-5908-6-42

39 Michie S, Richardson M, Johnston M, *et al.* The behavior change technique taxonomy (v1) of 93 hierarchically clustered techniques: building an international consensus for the reporting of behavior change interventions. *Ann Behav Med* 2013;46:81–95. doi:10.1007/s12160-013-9486-6

40 Michie S, Wood CE, Johnston M, *et al.* Behaviour change techniques: the development and evaluation of a taxonomic method for reporting and describing behaviour change interventions (a suite of five studies involving consensus methods, randomised controlled trials and analysis of qualitative data). *Health Technol Assess* 2015;19:1–188. doi:10.3310/hta19990

For peer review only

FIGURE LEGEND

Figure 1 REDUCE Maintenance Intervention Logic Model

SUPPLEMENTARY MATERIAL

Appendix 1: Interview schedule and prompt cards

Appendix 2: Behavioural analysis table

For peer review only - http://bmjopen.bmj.com/site/about/guidelines.xhtml

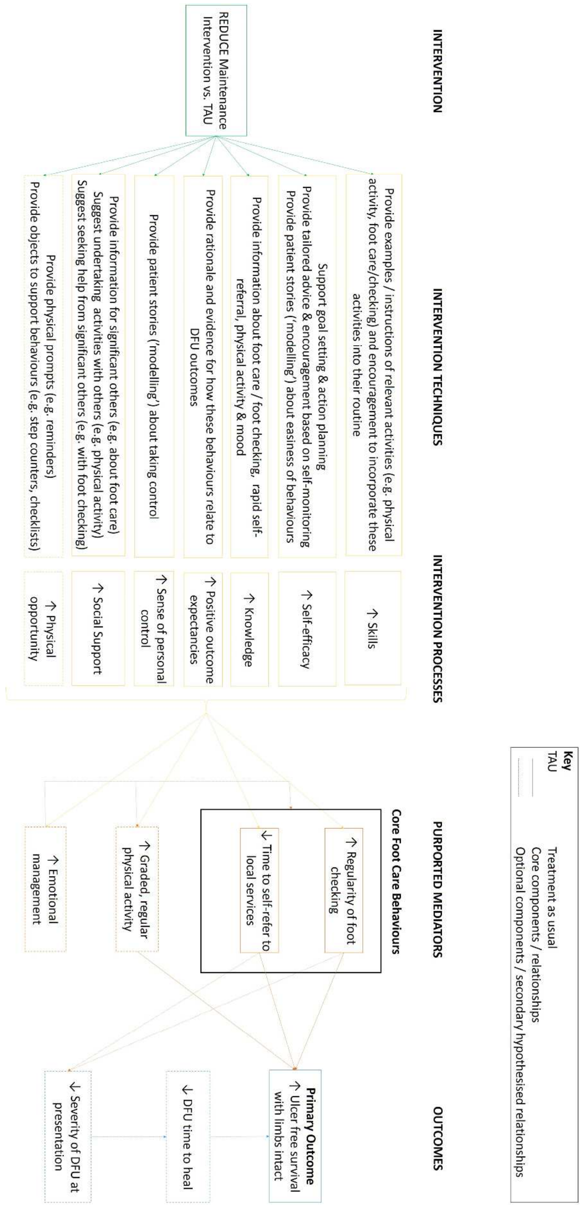

Figure 1 REDUCE Maintenance Intervention Logic Model

119x248mm (600 x 600 DPI)

APPENDIX 1: INTERVIEW SCHEDULE AND PROMPT CARDS

Interview schedule

Section 1: Context

Q1. Can you tell me a bit about what it has been like for you to have a foot ulcer?
Q2. Can you tell me about anything that you do to look after your feet currently?

Section 2: Acceptability and feasibility of the maintenance intervention

[Provide explanation of initiation phase and maintenance intervention]

1. Content of website/booklet

Card 1 (foot checking):

Q3. What do you think about these ideas? What do you like about these ideas? What do you dislike about these ideas?

Q4. The foot checker will ask you to check your feet every day for foot damage. What things might make it difficult for you to do this? Do you have any concerns about checking your feet every day?

Card 2 (help-seeking):

Q5. What do you think about these ideas? What do you like about these ideas? What do you dislike about these ideas?

Q6. The website may ask you to contact your diabetes team if you are concerned about any foot damage you have. What things might make it difficult for you to do this? Do you have any concerns about doing this?

Card 3 (physical activity):

Q7. What do you think about these ideas? What do you like about these ideas? What do you dislike about these ideas?

Q8. What things might make it difficult for you to get more active? Do you have any concerns about getting more active?

Card 4 (dealing with feelings):

Q9. What do you think about these ideas? What do you like about these ideas? What do you dislike about these ideas? Is there anything you do at the moment that helps you when you feel stressed / low?

Additional content:

Q10. What other things could we do to help you to look after your feet?

2. Delivery formats

Q11. What are your thoughts about the long-term support for maintaining habits being provided in **a booklet**? What do you like about this idea? What do you dislike about this idea?

For peer review only - http://bmjopen.bmj.com/site/about/guidelines.xhtml

Q12. What are your thoughts about the long-term support for maintaining habits being provided through **a website**? What do you like about this idea? What do you dislike about this idea?

Q13. If we were to deliver the programme through a website, what do you think about the idea of using **a computer or tablet**, such as an iPad, to access the website?

Q14. What do you think about the idea of using **a mobile phone** to access the website?

Card 5 (optional health professional support):

Q15. What do you think about this idea? What do you like about this idea? What do you dislike about this idea?

Q16. [If optional support would be helpful] What would you like to talk to the health professional about? Why would this be helpful?

Q17. You could contact the health professional in person, over the phone, and by email. Which one of these options would you prefer? Why?

Prompt Cards

Card 1 – Check your feet regularly

Why is this important?

It can be difficult to know when you might be developing a foot ulcer because some of the initial signs can be very small and hard to spot. It is important to examine your feet regularly so you are better able to spot any changes in your feet.

The website or booklet will:

- Ask you to check your feet every day
- Allow you to make a note of any changes in your feet

Set up regular reminders to check your feet which the foot checker can send to you by email or text messages to your mobile phone

Card 2 – What to do if you spot any foot damage

Why is this important?

It is important to report any changes in your feet to your diabetes team as soon as possible. This will allow them to check your feet and see if you need any treatment. The quicker your feet are treated, the more likely that any damage to your feet will heal.

The website or booklet will:

- Give you personalised advice on what to do if you spot any changes in your feet
- Advise you when you may need to contact your diabetes team

For peer review only - http://bmjopen.bmj.com/site/about/guidelines.xhtml

Card 3 – Getting active

Why is this important?

When you don't have a foot ulcer, it is safe and important to be mobile and active. This is because being active will improve your circulation and blood sugar, and reduce your chances of getting another ulcer. It is important that you pace your activity – little and often is best. Irregular activity (doing nothing and then doing too much) can be harmful because it increases the chances of injuring your feet.

The website or booklet will:

- Help you to slowly increase physical activity, such as using an exercise bike, seated exercise, walking, or any other things you like to do to be active.
- Set weekly physical activity goals, for example, going for a walk once or a few times a week.
- Ask you to enter in information about how you got on with your goals each week and provide you with personalised advice based on your progress.

A free step counter (or pedometer) that clips onto your belt and counts how many steps you take. You can use this to set yourself daily or weekly step goals if you would like to.

Card 4 – Dealing with your feelings when you get another ulcer

Why is this important?

We know that people who have had an ulcer can feel frustrated and cross if, and when, they get another one. Some people can feel down or stressed at this time and it can be difficult to look after yourself when you are feeling this way. Dealing with your feelings can make you feel better and will make sure you put you and your health first.

The website or booklet will:

- Teach you techniques that have been shown to help improve mood and reduce stress.
- Many people have found these techniques to be helpful.

Card 5 - Optional health professional support:

The website or booklet will also:

- Give people the option to contact a diabetes trained health professional if they wanted to.
- This contact could be in person, over the phone, or by email.

For peer review only

APPENDIX 2: BEHAVIOURAL ANALYSIS OF REDUCE MAINTENANCE INTERVENTION USING THE BEHAVIOUR CHANGE WHEEL (BCW) AND BEHAVIOUR CHANGE TECHNIQUES TAXONOMY (BCTv1)

Key: DFU = Diabetic Foot Ulcer; EO = Barrier emerged from expert opinion; LR = Barrier emerged from literature review; QR = Barrier emerged from qualitative primary research; N/A = theoretical mapping not applicable; * = intervention components and BCTs identified through examination of the BCTv1 and BCW to check for additional intervention functions, target constructs, or behaviour change techniques.

**Barriers/*facilitator* to target behaviour**	**Intervention components**	**Target construct (BCW)**	**Intervention function (BCW)**	**Behaviour Change Technique (using BCTv1)**
**Target behaviour: Engaging in regular foot checking**				
Belief that foot checking will do little to delay getting a DFU [EO; LR]	• Provide a rationale for the necessity of regular foot checking, including evidence that it is effective for delaying DFUs	Psychological capability; Reflective motivation	Education; Persuasion	5.1 Information about health consequences<br>5.6 Information about emotional consequences
	• Provide patient stories demonstrating how regular foot checking helped other patients to take control of their DFUs	Psychological capability; Reflective motivation; Social opportunity	Education; Persuasion; Modelling	5.1 Information about health consequences<br>6.2 Social comparison<br>6.3 Information about others' approval<br>9.1 Credible source
Lack of knowledge regarding DFUs and foot checking procedures/ Lack of confidence in ability to check feet [LR, QR]	• Provide information and pictures on what DFUs look like, what signs of DFUs to look out for, and how to check their feet	Physical capability; Psychological capability	Education; Training	4.1 Instructions on how to perform the behaviour<br>5.1 Information about health consequences
	• Provide an online and printable foot health checklist so patients can spot changes in their foot health<br>• Allow patients to record any changes in their foot health	Psychological capability; Physical opportunity	Training; Environmental structuring	4.1 Instructions on how to perform the behaviour<br>12.5 Adding objects to the environment
	• Provide patient stories demonstrating how easy it was for other patients to engage in regular foot checking	Reflective motivation; Social opportunity	Persuasion; Modelling	6.2 Social comparison<br>6.3 Information about others' approval<br>9.1 Credible source
Forgetting to check your feet [EO, QR]<br><br>*Reminders to check your feet [EO,QR]*	• Allow patients to set up regular reminders to check your feet daily by email or text messages and decide on the frequency of these reminders	Physical opportunity	Environmental structuring	7.1 Prompts/cues

For peer review only - http://bmjopen.bmj.com/site/about/guidelines.xhtml

Barriers/*facilitator* to target behaviour	Intervention components	Target construct (BCW)	Intervention function (BCW)	Behaviour Change Technique (using BCTv1)
Difficulties engaging in foot checking in the long-term [LR, QR]<br><br>*Integrating foot checking into your routine [QR]*	• Allow patients to set their own daily foot checking goals<br>• Encourage patients to make a foot checking action plan	Reflective motivation	Enablement	1.1 Goal setting (behaviour)<br>1.4 Action planning<br>1.5 Review behaviour goal(s)
	• Advise patients to regularly practice foot checking in the same context (e.g. after showering, when putting socks on)	Psychological capability; Automatic motivation	Training; Enablement	8.1 Behavioural practice/rehearsal<br>8.3 Habit formation
Physical limitations, deformities and barriers (e.g. limited mobility, foot deformities, poor eyesight) [LR, QR]<br><br>*Using a mirror [QR]; Getting someone to check your feet for you [QR]*	• Provide guidance on how to check your feet if you have physical limitations, including using a mirror to check the bottom of your feet and asking someone else to check for you<br>• Provide information on the signs of DFUs and foot checking procedures for significant others who are helping with foot checking	Physical capability; Psychological capability; Social opportunity	Training; Education; Enablement	3.2 Social support (practical)<br>4.1 Instructions on how to perform the behaviour
**Target behaviour: Engaging in rapid self-referral in the event of changes in foot health**				
Belief that self-referral will <u>do little</u> to aid DFU healing [EO, LR] /Lack of confidence in DFU team [QR]	• Provide a rationale for the necessity of reporting any signs of foot damage, including evidence that this is effective for DFU healing	Psychological capability; Reflective motivation	Education; Persuasion	5.1 Information about health consequences<br>5.6 Information about emotional consequences
	• Provide patient stories demonstrating how rapid self-referral helped other patients to take control of their DFUs, and how they overcame previous frustrations with the DFU team and feelings that it was not worth it	Psychological capability; Reflective motivation; Social opportunity	Education; Persuasion; Modelling	5.1 Information about health consequences<br>6.2 Social comparison<br>6.3 Information about others' approval<br>9.1 Credible source
Lack of understanding regarding when to seek help and who to contact [EO, QR]	• Provide advice on when you may need to contact your diabetes team<br>• Advise patients to find out the contact details of their DFU team	Psychological capability	Education	4.1 Instructions on how to perform the behaviour

For peer review only - http://bmjopen.bmj.com/site/about/guidelines.xhtml

**Barriers/*facilitator* to target behaviour**	**Intervention components**	**Target construct (BCW)**	**Intervention function (BCW)**	**Behaviour Change Technique (using BCTv1)**
Difficulty accessing the DFU team and getting a quick appointment [EO, QR]	• Provide information on the national guidelines regarding timeline for referrals to DFU team<br>• Invite patients to refer to their local procedure for self-referrals given in their REDUCE action plan in the initiation phase<br>• Provide advice on how to communicate the reason for self-referral when contacting the DFU team	Psychological capability	Education	4.1 Instructions on how to perform the behaviour
Forgetting the contact details for the foot care team [EO]	• Invite patients to record the contact details of their foot care team and print this record to act as a reminder	Physical opportunity	Environmental structuring	7.1 Prompts/cues<br>12.5 Adding objects to the environment
Forgetting to contact their foot care team [EO]	• Invite patients to set up reminders to contact their foot care team if they record any signs of foot damage into the maintenance intervention and decide on the frequency of these reminders	Physical opportunity	Environmental structuring	7.1 Prompts/cues
Concerns about looking foolish or wasting the DFU team's time when reporting changes in foot health that turn out to be normal/ Not wanting to bother the DFU team [EO, QR]	• Reassure patients that health professionals would rather they were contacted early so they are better able to treat the DFU<br>• Provide patient stories on how other patients overcame feelings of being a burden<br>• Provide personalised feedback on whether or not they should self-refer, based on the answers they give to the foot health checklist	Psychological capability; Reflective motivation	Education; Persuasion	4.1 Instructions on how to perform the behaviour<br>6.3 Information about others' approval
**Target behaviour: Engaging in graded and regular physical activity**				
Belief that physical activity will do little to delay getting a DFU [EO, LR]<br><br>*Awareness of non-DFU related benefits of physical activity that are immediate and salient [EO]*	• Provide a rationale for the necessity of graded and regular physical activity and evidence that it is effective for delaying DFUs<br>• Provide a quiz about the benefits of physical activity for delaying DFUs, including other physical and mental benefits (e.g. improved sleep and energy, alleviation from aches and pains)	Psychological capability; Reflective motivation	Education; Persuasion	5.1 Information about health consequences<br>5.6 Information about emotional consequences
	• Provide patient stories demonstrating how graded and regular physical activity helped other patients to take control of their DFUs and led to other salient benefits	Reflective motivation; Social opportunity	Education; Persuasion; Modelling	5.1 Information about health consequences<br>6.2 Social comparison<br>6.3 Information about others' approval<br>9.1 Credible source

For peer review only - http://bmjopen.bmj.com/site/about/guidelines.xhtml

**Barriers/*facilitator* to target behaviour**	**Intervention components**	**Target construct (BCW)**	**Intervention function (BCW)**	**Behaviour Change Technique (using BCTv1)**
Concerns regarding the safety of physical activity [EO, QR]	• Reassure patients that gradual physical activity is safe (e.g. shouldn't cause too much shoe rubbing) and can be done when you do not have a DFU<br>• Address patients' individual physical activity concerns in the initiation phase	Psychological capability	Education	4.1 Instruction on how to perform the behaviour<br>5.1 Information about health consequences
Lack of belief in one's ability to engage in physical activity [LR] /Physical limitations (e.g. arthritis, breathlessness, foot discomfort/pain) [QR]/Bad weather [QR]<br><br>*Finding a suitable activity [QR]*	• Provide patient stories demonstrating how easy it was for other patients to engage in graded and regular physical activity, even though they are at high risk of developing DFUs or have health problems	Reflective motivation; Social opportunity	Persuasion; Modelling	6.2 Social comparison<br>6.3 Information about others' approval<br>9.1 Credible source
	• Provide a variety of examples of safe low-to-moderate physical activity, including activities that are non-weight bearing and can be done in bad weather	Psychological capability	Education	4.1 Instruction on how to perform the behaviour
Difficulties engaging in physical activity in the long-term [LR, QR]<br><br>*Social support [LR]; Provision of pedometers [LR, QR]; Integrating physical activity into your routine [QR]*	• Invite patients to set their own weekly physical activity goals<br>• Invite patients to self-monitor physical activity and provide personalised advice on how to modify goals based on self-monitoring<br>• Invite patients to make a physical activity action plan<br>• Invite patients to set easy-to-perform tasks and make them increasingly more difficult over time*	Reflective motivation; Psychological capability	Enablement; Training	1.1 Goal setting (behaviour)<br>1.4 Action planning<br>1.5 Review behaviour goal(s)<br>2.2 Feedback on behaviour<br>2.3 Self-monitoring of behaviour<br>8.7 Graded tasks*
	• Provide a free pedometer to those who would like one and encourage people to set daily step goals	Reflective motivation; Psychological capability; Physical opportunity	Enablement; Training; Environmental structuring	1.1 Goal setting (behaviour)<br>2.2 Feedback on behaviour<br>2.3 Self-monitoring of behaviour<br>12.5 Adding objects to the environment
	• Suggest that patients ask a friend/relative to exercise with them	Social opportunity	Enablement	3.1 Social support (unspecified)
	• Invite patients to regularly practice physical activity in the same context (e.g. after lunch)	Psychological capability; Automatic motivation	Training; Enablement	8.1 Behavioural practice/rehearsal<br>8.3 Habit formation

For peer review only - http://bmjopen.bmj.com/site/about/guidelines.xhtml

**Barriers/*facilitator* to target behaviour**	**Intervention components**	**Target construct (BCW)**	**Intervention function (BCW)**	**Behaviour Change Technique (using BCTv1)**
Forgetting to engage in physical activity [EO]<br><br>*Reminders [EO, LR]*	• Invite patients to set up email reminders to engage in physical activity and decide on the frequency of these reminders	Physical opportunity	Environmental structuring	7.1 Prompts/cues
**Target behaviour: Engaging in emotional management**				
Belief that emotional management will do little to delay getting a DFU or help with difficult emotions [EO; QR]	• Explain the necessity of emotional management for promoting engagement with the other foot care behaviours and provide evidence that they are effective for dealing with difficult emotions	Psychological capability; Reflective motivation	Education; Persuasion	5.1 Information about health consequences<br>5.6 Information about emotional consequences
	• Provide patient stories demonstrating how emotional management techniques helped other patients to take control of their DFUs	Psychological capability; Reflective motivation; Social opportunity	Education; Persuasion; Modelling	5.1 Information about health consequences<br>6.2 Social comparison<br>6.3 Information about others' approval<br>9.1 Credible source
Belief that emotional management is not relevant to them [QR]	• Provide emotional management as an optional part of the intervention<br>• Remind patients about emotional management at times of stress (e.g. if the foot health checklist highlights that they may have signs of getting a DFU)	N/A	N/A	N/A
Belief that the emotional management techniques do not fit with their preferable approach to emotional management [QR]	• Provide a range of techniques that may fit with a patients' preferred approach to emotional management (e.g. cognitive and behavioural techniques)	N/A	N/A	N/A
Lack of understanding regarding how to do the emotional management techniques [EO]	• Provide guidance on how to do the emotional management techniques	Psychological capability	Training	4.1 Instructions on how to perform the behaviour
	• Provide guided audio recordings of emotional management exercises	Physical opportunity	Environmental structuring	12.5 Adding objects to the environment
Lack of confidence in ability to practice emotional management techniques [EO]	• Provide patient stories demonstrating how easy it was for other patients to practice the emotional management techniques	Reflective motivation; Social opportunity	Persuasion; Modelling	6.2 Social comparison<br>6.3 Information about others' approval<br>9.1 Credible source

For peer review only

**Barriers/*facilitator* to target behaviour**	**Intervention components**	**Target construct (BCW)**	**Intervention function (BCW)**	**Behaviour Change Technique (using BCTv1)**
Difficulties engaging in emotional management in the long-term [EO]	• Invite patients to set their own emotional management practice goals	Reflective motivation	Enablement	1.1 Goal setting (behaviour)<br>1.4 Action planning<br>1.5 Review behaviour goal(s)
**Subsidiary behaviour: Engaging with the digital maintenance intervention**				
Low confidence in ability to use digital interventions [LR; QR]<br><br>*Technical support to use digital interventions [EO]*	• Health professionals introduce the digital maintenance intervention in the initiation phase and provide technical support as required<br>• Suggest that family and friends could assist the patient with digital intervention use, if appropriate	Physical capability; Psychological capability; Social opportunity	Training; Education; Enablement	3.2 Social support (practical)<br>4.1 Instructions on how to perform the behaviour
	• Health professionals will speak favourably about digital intervention use and outline its advantages<br>• Build patients' confidence in using the digital intervention by demonstrating how easy it is to use the intervention, and addressing any self-doubts*	Reflective motivation	Education; Persuasion	9.1 Credible source<br>15.1 Verbal persuasion about capability*

For peer review only

Greenwell, Sivyer et al. Intervention planning for the REDUCE maintenance intervention: a digital intervention to reduce re-ulceration risk among patients with a history of diabetic foot ulcers

COREQ checklist

No	Item	Guide questions/description		Location in manuscript
**Domain 1: Research team and reflexivity**				
Personal Characteristics				
1.	Interviewer/facilitator	Which author/s conducted the interview or focus group?	KG & KS	Qualitative interviews Methods – Page 11
2.	Credentials	What were the researcher's credentials? *E.g. PhD, MD*	KG – PhD, MSc, CPsychol.<br>KS – DPhil (graduand), MSc	-
3.	Occupation	What was their occupation at the time of the study?	KG – Health Psychologist & Research Fellow<br>KS – Research Fellow	-
4.	Gender	Was the researcher male or female?	Both female	-
5.	Experience and training	What experience or training did the researcher have?	Both interviewers received training on qualitative research methods in their MScs and have carried out several qualitative interview research projects.	-
Relationship with participants				
6.	Relationship established	Was a relationship established prior to study commencement?	Participants were not known to the interviewers prior to recruitment.	-
7.	Participant knowledge of the interviewer	What did the participants know about the researcher? e.*g. personal goals, reasons for doing the research*	Participants were told that the research findings were being used to develop an intervention to support them to look after their feet and improve their foot health.	-
8.	Interviewer characteristics	What characteristics were reported about the interviewer/facilitator? e.g. *Bias, assumptions, reasons and interests in the research topic*	Both interviewers are psychologists with a specialist interest in digital health interventions, which could be a potential source of bias. No other interviewer-related biases identified.	-

For peer review only

Greenwell, Sivyer et al. Intervention planning for the REDUCE maintenance intervention: a digital intervention to reduce re-ulceration risk among patients with a history of diabetic foot ulcers

**Domain 2: study design**				
Theoretical framework				
9.	Methodological orientation and Theory	What methodological orientation was stated to underpin the study? *e.g. grounded theory, discourse analysis, ethnography, phenomenology, content analysis*	Pragmatism & thematic analysis.	Qualitative interviews Methods – Page 11
Participant selection				
10.	Sampling	How were participants selected? *e.g. purposive, convenience, consecutive, snowball*	Purposive sampling.	Qualitative interviews Methods – Page 10
11.	Method of approach	How were participants approached? e.g. *face-to-face, telephone, mail, email*	Mail. We also advertised the research via social media channels and the website for a national charity, and used opportunistic recruitment by health professionals during consultations, however, no participants were recruited using these methods.	Qualitative interviews Methods – Page 10
12.	Sample size	How many participants were in the study?	20	Qualitative interviews Methods – Page 10
13.	Non-participation	How many people refused to participate or dropped out? Reasons?	Of those approached, 26% (66/250) expressed an interest to take part. Of the 21 eligible people we contacted for interview, only one did not go on to complete the interview due to competing time commitments. There were no withdrawals.	Qualitative interviews Methods – Page 10
Setting				
14.	Setting of data collection	Where was the data collected? e.g. *home, clinic, workplace*	At participants houses or the university	Qualitative interviews Methods – Page 11
15.	Presence of non-participants	Was anyone else present besides the	Relatives were occasionally present	-

For peer review only

Greenwell, Sivyer et al. Intervention planning for the REDUCE maintenance intervention: a digital intervention to reduce re-ulceration risk among patients with a history of diabetic foot ulcers

		participants and researchers?	during the interviews.	
16.	Description of sample	What are the important characteristics of the sample? *e.g. demographic data, date*	Demographic information can be found in Table 2. Interviews were carried out between April to May 2017.	Qualitative interviews Methods – Table 2 – Pages 11&12
Data collection				
17.	Interview guide	Were questions, prompts, guides provided by the authors? Was it pilot tested?	The interview schedule and prompt cards can be found in Appendix 1. They were pilot tested with two people with a history of diabetic foot ulcers.	Qualitative interviews Methods – Pages 10&11
18.	Repeat interviews	Were repeat interviews carried out? If yes, how many?	Only single interviews were carried out.	Qualitative interviews Methods – Page 10
19.	Audio/visual recording	Did the research use audio or visual recording to collect the data?	The audio from the interviews was digitally recorded.	Qualitative interviews Methods – Page 11
20.	Field notes	Were field notes made during and/or after the interview or focus group?	No	-
21.	Duration	What was the duration of the interviews or focus group?	Interviews lasted between 36 and 99 minutes	-
22.	Data saturation	Was data saturation discussed?	Participants were recruited until data saturation was reached.	-
23.	Transcripts returned	Were transcripts returned to participants for comment and/or correction?	No	-
**Domain 3: analysis and findings**				
Data analysis				
24.	Number of data coders	How many data coders coded the data?	Two (KS & KG)	Qualitative interviews Methods – Page 11
25.	Description of the coding tree	Did authors provide a description of the coding tree?	No	-
26.	Derivation of themes	Were themes identified in advance or derived from the	Major themes were identified in advance.	-

For peer review only

Greenwell, Sivyer et al. Intervention planning for the REDUCE maintenance intervention: a digital intervention to reduce re-ulceration risk among patients with a history of diabetic foot ulcers

		data?		
27.	Software	What software, if applicable, was used to manage the data?	QSR's NVivo 11 was used.	-
28.	Participant checking	Did participants provide feedback on the findings?	No	-
Reporting				
29.	Participant checking	Were participant quotations presented to illustrate the themes / findings? Was each quotation identified? e.g. *participant number*	Quotations were provided to illustrate each key finding. They are identified by a participant number and their gender is noted.	Qualitative interviews Results – Table 3 – Pages 12&13
30.	Data and findings consistent	Was there consistency between the data presented and the findings?	Yes	Qualitative interviews Results – Table 3 – Pages 12&13
31.	Clarity of major themes	Were major themes clearly presented in the findings?	Yes	Qualitative interviews Results – Table 3 – Pages 12&13
32.	Clarity of minor themes	Is there a description of diverse cases or discussion of minor themes?	Diverse cases are discussed.	Qualitative interviews Results – Pages 12-16

For peer review only

BMJ Open

Intervention planning for the REDUCE maintenance intervention: a digital intervention to reduce re-ulceration risk among patients with a history of diabetic foot ulcers

Journal:	*BMJ Open*
Manuscript ID	bmjopen-2017-019865.R2
Article Type:	Research
Date Submitted by the Author:	12-Feb-2018

Complete List of Authors:	Greenwell, Kate; Centre for Clinical and Community Applications of Health Psychology, University of Southampton<br>Sivyer, Katy; Centre for Clinical and Community Applications of Health Psychology, University of Southampton<br>Vedhara, Kavita; University of Nottingham Division of Primary Care<br>Yardley, Lucy ; Centre for Clinical and Community Applications of Health Psychology, University of Southampton<br>Game, Frances; Derby Teaching Hospitals NHS Foundation Trust<br>Chalder, Trudie; King's College London, Department of Psychological Medicine<br>Richards, Gayle; Northern Devon Healthcare NHS Trust, Department of Diabetes<br>Drake, Nikki; Bristol Community Health<br>Gray, Katie; Derby Teaching Hospitals NHS Foundation Trust<br>Weinman, John; King's College London Institute of Pharmaceutical Science<br>Bradbury, Katherine; Centre for Clinical and Community Applications of Health Psychology, University of Southampton

**Primary Subject Heading**:	Diabetes and endocrinology
Secondary Subject Heading:	Qualitative research, Patient-centred medicine, Research methods
Keywords:	Diabetic foot < DIABETES & ENDOCRINOLOGY, Diabetes & endocrinology < INTERNAL MEDICINE, WOUND MANAGEMENT

SCHOLARONE™
Manuscripts

Intervention planning for the REDUCE maintenance intervention: a digital intervention to reduce re-ulceration risk among patients with a history of diabetic foot ulcers

Kate Greenwell[1]*[a], Katy Sivyer[1]*, Kavita Vedhara[2], Lucy Yardley[1], Frances Game[3], Trudie Chalder[4], Gayle Richards[5], Nikki Drake[6], Katie Gray[3], John Weinman[7], Katherine Bradbury[1]

*Kate Greenwell and Katy Sivyer contributed equally to this paper

[a]Corresponding author: Centre for Clinical and Community Applications of Health Psychology, University of Southampton, Shackleton Building, Highfield Campus, Southampton, SO17 1BJ, UK; Email: reduceproject@soton.ac.uk; Tel: +44 (0) 2380 5950

[1]Centre for Clinical and Community Applications of Health Psychology, University of Southampton, Southampton, UK

[2]Division of Primary Care, School of Medicine, University of Nottingham, Nottingham, UK.

[3]Derby Teaching Hospitals NHS Foundation Trust, Derby, UK

[4]Department of Psychological Medicine, King's College London, London, UK

[5]Department of Diabetes, Northern Devon Healthcare NHS Trust, Barnstaple, UK

[6]Bristol Community Health, Bristol, UK

[7]Institute of Pharmaceutical Science, King's College London, London, UK

Word count: 4705 words

For peer review only

For peer review only - http://bmjopen.bmj.com/site/about/guidelines.xhtml

ABSTRACT

Objectives: To develop a comprehensive intervention plan for the REDUCE maintenance intervention to support people who have had diabetic foot ulcers (DFUs) to sustain behaviours that reduce re-ulceration risk.

Methods: Theory-, evidence- and person-based approaches to intervention development were used. In phase 1 of intervention planning, evidence was collated from a scoping review of the literature and qualitative interviews with patients who have had DFUs (N=20). This was used to identify the psychosocial needs and challenges of this population, and barriers and facilitators to the intervention's target behaviours: regular foot checking, rapid self-referral in the event of changes in foot health, graded and regular physical activity, and emotional management. In phase 2, this evidence was combined with expert consultation to develop the intervention plan. Brief 'guiding principles' for shaping intervention development were created. 'Behavioural analysis' and 'logic modelling' were used to map intervention content onto behaviour change theory to comprehensively describe the intervention and its hypothesised mechanisms.

Results: Key challenges to the interventions' target behaviours included patients' uncertainty regarding when to self-refer, physical limitations affecting foot checking and physical activity, and, for some, difficulties managing negative emotions. Important considerations for the intervention design included a need to increase patients' confidence in making a self-referral and in using the maintenance intervention, and a need to acknowledge that some intervention content might be relevant to only some patients (emotional management, physical activity). The behavioural analysis identified the following processes hypothesised to facilitate long-term behaviour maintenance including; increasing patients' skills, self-

For peer review only - http://bmjopen.bmj.com/site/about/guidelines.xhtml

efficacy, knowledge, positive outcome expectancies, sense of personal control, social support, and physical opportunity.

Conclusions: This research provides a transparent description of the intervention planning for the REDUCE maintenance intervention. It provides insights into potential barriers and facilitators to the target behaviours and potentially useful behaviour change techniques to use in clinical practice.

STRENGTHS AND LIMITATIONS OF THIS STUDY

- This research will inform the development of a novel intervention to support the prevention and management of DFUs and is in keeping with recent NICE research priorities for the diabetic foot.
- The integration of theory- evidence- and person-based approaches provided complementary insights into how an intervention could be designed to maximise its acceptability, feasibility, and potential effectiveness.
- The REDUCE maintenance intervention plan is comprehensively described and the intervention's potential mechanisms of actions made explicit, thereby increasing transparency, and facilitating application of this intervention planning methodology by other intervention developers.
- Although the qualitative sample was representative of patients with a DFU (who tend to be older and may therefore be retired), few younger and employed people were recruited so their views remain less well understood.
- Although the rapid scoping review allowed scientific evidence to be quickly incorporated into the intervention plan at an early stage, it was not systematic, so it is possible that some literature may have been missed.

For peer review only - http://bmjopen.bmj.com/site/about/guidelines.xhtml

BACKGROUND

Foot ulceration is a common, chronic, and costly complication of diabetes.[1–3] Healing is slow and recurrence is common, with approximately 40% of patients re-ulcerating within 12 months.[4–6] The physical and emotional burden of ulceration is considerable; 20% of ulcers result in amputation and 32% of patients are depressed, which is associated with a threefold greater risk of mortality.[2,7] Although diabetic foot care has been deemed a priority,[2] treatments to prevent ulceration are based largely on expert opinion and small, underpowered, studies.[2,8] Systematic reviews have found no evidence that education alone improves clinical outcomes.[9–12] However, research suggests that psychosocial and behavioural factors may play a central role in healing and prevention.[13]

Evidence suggests an association between longer delays in help seeking and increased ulcer severity, highlighting the importance of regular foot-checking and rapid self-referral.[14] Although physical activity is generally encouraged in diabetes to promote glycaemic control and reduce cardiovascular risk, there is a common assumption that greater physical activity may increase ulceration risk in people at risk of DFUs. However, research suggests that moderate, regular activity may decrease risk, or at worst, be unrelated to risk.[15,16] Emotional management may also play a role. Following a diabetic foot ulcer (DFU), people may experience difficult emotions, including depression, blame, and guilt.[17] Depression has been associated with greater ulcer incidence and recurrence, and a slower rate of ulcer healing.[18–20] NICE have consequently recommended the development of new interventions targeting such factors.[2]

'REDUCE', a novel complex cognitive behavioural intervention,[21] was developed to reduce re-ulceration risk and promote healing by modifying associated psychological and behavioural factors.[22] These factors include; non-adherence to recommended foot care

For peer review only - http://bmjopen.bmj.com/site/about/guidelines.xhtml

procedures (e.g. foot checking), delayed help-seeking for changes in foot health, low or irregular levels of physical activity, and difficulties in managing negative emotions.

REDUCE consists of two phases; an initiation phase of eight weekly sessions with a nurse or podiatrist to start psychological and behavioural change, and a maintenance phase involving two additional sessions held one and three months later to help sustain these changes. A full description of the intervention can be found in Vedhara et al.[22]A feasibility study found REDUCE to be acceptable and feasible for patients and preliminary descriptive findings suggested that patients experienced changes in many of the psychological and behavioural factors targeted by the intervention.[22] However, long-term maintenance of these changes may be more effective if the intervention were available indefinitely, and when patients require it. Low-intensity interventions delivered by websites, smartphones, or a booklet provide a low-cost solution. This paper describes the planning process for an intervention that will replace the face-to-face maintenance sessions of the original intervention.

The key objective of the REDUCE maintenance intervention will be to provide support to people who have had diabetic foot ulcers (DFUs) to increase their ulcer free survival with limbs intact (i.e. the length of time a patient is free from ulcers without having had an amputation). In keeping with recent NICE research priorities, this will be done through behaviour change and emotional management. It will support people to maintain four behaviours targeted in the initiation phase: regular foot checking, rapid self-referral in the event of changes in foot health, graded and regular physical activity, and emotional management.

Published descriptions of complex interventions and their development process are often inadequate, providing readers with little understanding of what the intervention contains, how decisions regarding its development were made, and how the intervention is hypothesised to

For peer review only - http://bmjopen.bmj.com/site/about/guidelines.xhtml

work.[12,23–25] This paper presents the full intervention planning process for the REDUCE maintenance intervention as an example of intervention planning methodology and to increase transparency regarding the intervention's content and hypothesised mechanisms of action. This intervention plan will subsequently inform the development of the REDUCE maintenance intervention.

METHODS AND RESULTS

Intervention planning methodology

The intervention planning used theory-, evidence- and person-based approaches.[21,26–28] The person-based approach recommends grounding intervention development in an in-depth understanding of the patient and their psychosocial context, gained through qualitative research.[26] Intervention planning included two phases: collating and analysing evidence; and creating the intervention plan. Phase one includes two elements: a qualitative and quantitative scoping review, and a qualitative interview study. Phase two includes three elements: 1) creating guiding principles; 2) behavioural analysis; and 3) logic modelling.

In phase one, a rapid scoping review of qualitative and quantitative literature was used to examine the behavioural and psychosocial needs, issues, and challenges of people who have had DFUs. This knowledge was combined with insights gained from a qualitative interview study that explored patients' perspectives on key content and design features for the maintenance intervention. These two studies are both person- and evidence-based approaches as they aim to develop an in-depth understanding of the patients' perspective (person-based approach), while identifying, summarising, and incorporating the evidence-base on the barriers and facilitators to the target behaviours (evidence-based approach). The findings of these two studies were given equal weight when creating the intervention plan.

For peer review only - http://bmjopen.bmj.com/site/about/guidelines.xhtml

We also consulted with experts in diabetic foot ulcers, behaviour change, and intervention development who belonged to our multidisciplinary project team using regular teleconferences to discuss and gain feedback on drafts of the intervention plan. This team included one diabetologist, two diabetes specialist podiatrists, one diabetes specialist nurse, one cognitive behavioural psychotherapist, five health psychologists, and one research psychologist specialising in health. From this, additional barriers and facilitators were identified, and suggestions or refinements to intervention content were made.

In line with a person-based approach,[26] all sources of evidence (i.e. scoping review, qualitative study results, expert opinion) were brought together in phase two to create 'guiding principles' that outline the intervention design objectives and key intervention features. Theory-based 'behavioural analysis' and 'logic modelling' [25,28,29] were used to map the evidence and intervention content onto behaviour change theory to comprehensively describe the intervention and its potential mechanisms of action.

Collating and analysing evidence

Qualitative and quantitative scoping review

Purpose

To review evidence examining the behavioural and psychosocial needs, issues, and challenges of people who have had DFUs.

Methods

A rapid scoping review of the qualitative and quantitative literature exploring patients' and health professionals' views and experiences of DFUs and their management was undertaken. This was done to ensure that the initial intervention plan was informed by existing evidence from an early stage. A search was undertaken in Web of Science (covering 1970-2017) to ensure coverage of a range of multidisciplinary journals, easily enabling rapid review. This

For peer review only - http://bmjopen.bmj.com/site/about/guidelines.xhtml

search combined the following terms (“diabetic foot ulcer”) AND (“physical activity” OR exercise), (“self-referral” OR “help seeking”), (check AND (foot OR feet)), and (“emotional management” OR “mood management”). It incorporated any published research that included patients who had previously had a diabetic foot ulcer. Findings regarding beliefs around foot care were excluded if they were only relevant to foot care behaviours not targeted in the REDUCE maintenance intervention (e.g. barriers to adherence to prescription footwear). Articles with a biological focus were excluded. Additional literature was identified through expert consultation and article reference lists. Data were extracted on research design, sample size, participants, and key findings. Using thematic analysis, the key findings were organised into themes relating to the psychosocial and behavioural issues, needs, or challenges to be considered during intervention development.

Results

The review identified seven articles and highlighted six themes relating to people’s beliefs around DFUs and the target behaviours, challenges people face when engaging in the target behaviours, difficult emotions people may experience following a DFU, and concerns about digital interventions (Table 1).

For peer review only

For peer review only - http://bmjopen.bmj.com/site/about/guidelines.xhtml

Table 1 Key themes identified from the rapid scoping review of the psychosocial and behavioural issues, needs and challenges of people who have had DFUs

Key themes	Detail from the literature
Lack confidence in foot checking [17,30]	• Some patients were uncertain about what a DFU was or looked like, what signs of DFUs to look out for, and when the DFU was serious enough to seek help from a health professional. Such uncertainties may lead to delays in seeking help.
Feelings of lack of control in preventing DFUs [17,30]	• Some patients felt they had little or no control in preventing further DFUs, as DFUs still occurred even when they were engaging in foot care behaviours.<br>• Some patients believed that they were unable to prevent DFUs.
Difficult emotions following a DFU [17,31–34]	• Some patients were fearful or worried about developing further DFUs, losing limbs through amputation, and the impact a DFU reoccurrence might have on their lives.<br>• Some patients felt down or had low self-esteem because of how the DFUs had negatively affected their everyday lives (e.g. loss of independence, inability to work and provide for the family, lifestyle changes).<br>• Some patients felt a sense of hopelessness, anger, and frustration when DFUs developed despite their attempts to engage in foot care behaviours.<br>• Some patients felt self-blame or guilt for not paying enough attention to their feet, not controlling their diabetes well, not following foot care advice, or not engaging in foot care behaviours, especially in the event of reoccurrence.<br>• Some patients experienced social isolation (e.g. from restricted mobility, lack of employment) or felt a burden to others because they were dependent on them for daily activities (e.g. cooking and driving).<br>• Some patients found it difficult to share their experiences of a DFU with friends and family.<br>• Some podiatrists acknowledged the emotional impact of DFUs on their patients, specifically the presence of anger, depression, anxiety, and frustration.
Maintaining behaviours long-term may be challenging [17]	• Some patients were not confident that they could maintain foot care behaviours in the long-term, with engagement likely to decrease over time.<br>• Some patients were impatient to resume the physical activities they stopped when they had an active DFU, leading them to do too much activity and risk getting another DFU.
Physical limitations impeding foot checking [34,35]	• Some patients and podiatrists reported physical limitations that prevented patients from engaging in foot care behaviours, including joint mobility problems, neuropathy, and visual impairment.
Concerns over using digital interventions [32]	• Some patients felt they did not have the necessary computer skills for internet or computer-based interventions.

For peer review only - http://bmjopen.bmj.com/site/about/guidelines.xhtml

Qualitative interviews

Purpose

To explore the acceptability and feasibility of initial ideas regarding the content and delivery of the maintenance intervention from the perspective of people who have had DFUs; and to identify potential barriers and facilitators to its target behaviours.

Methods

A total of 250 adult (aged 18+ years) patients with diabetes who had previously had a DFU were contacted by letter by their local NHS podiatry service. Participants were excluded if they had a DFU in the previous two weeks. Sixty-six patients (26%) expressed interest in the study, 53 of whom (21% of original mail-out) were eligible to participate. Eligible respondents were purposively sampled to represent a diverse set of ages (range: 45-91 years), genders, and internet use (Table 2). Twenty participants took part in a single semi-structured interview.

Interviews explored participants' views of the target behaviours and potential intervention features, including foot checking reminders, facilities for note-taking, personalised advice about when to self-refer, advice on pacing physical activity, goal setting, provision of free pedometers, and emotional management techniques. Interviews also explored participants' views on possible modes of intervention delivery, including booklet, website, computer tablet, and smartphones, and the value of additional health professional input. Ideas for potential content, intervention features, and delivery modes were shown on prompt cards. Ideas for intervention features (e.g. pedometers) were chosen based on the multidisciplinary team's knowledge of the evidence for the acceptability and effectiveness of these features for changing the target behaviours. Participants were shown an example of an existing diabetes intervention [36] to demonstrate what a website intervention could look like. Interviews were

For peer review only - http://bmjopen.bmj.com/site/about/guidelines.xhtml

piloted with two people who have had DFUs. See Appendix 1 for the interview schedule and prompt cards.

Interviews were carried out by KG and KS and took place at participants' homes (N=18) or the university (N=2). Participants were reimbursed for travel and given a £10 voucher. All interviews were recorded and transcribed. KG and KS used thematic analysis to identify potential barriers and facilitators to engaging with the target behaviours, and positive and negative perceptions of the potential intervention features and delivery modes. Ethical approval for this study was gained from North West – Greater Manchester West Research Ethics Committee (17/NW/0024).

Table 2 Demographics of patients taking part in the qualitative interviews

Sample Characteristics	Statistics
**Basic Demographics**	**Mean (SD)**
Age	68.30 (11.54)
**Basic Demographics**	**N (%)**
Male	11 (55%)
Marital Status	
Married	7 (35%)
Single	6 (30%)
Widowed	4 (20%)
Divorced	3 (15%)
Employment Status	
Retired	15 (75%)
Redundant due to illness	3 (15%)
Housewife/husband	1 (5%)
Full-time employed	1 (5%)
Educational Status	
Secondary School	10 (50%)
College / Sixth Form / Professional Qualification	7 (35%)
Undergraduate	3 (15%)
**DFU History**	**Mean (SD)**
Years since first DFU (approx.)	6.81 (7.96)
Number of DFUs (approx.)	4.18 (3.86)
Months since last DFU (approx.)	14.65 (11.26)
Duration of last DFU in days (approx.)	298 (400.82)
**Internet Use**	**N (%)**
Access to internet at home	15 (75%)
Access to internet on tablet	7 (35%)
Access to internet on phone	3 (15%)
Frequency of access	
Never	3 (15%)
Less than once a month	3 (15%)
Once a week	1 (5%)

For peer review only - http://bmjopen.bmj.com/site/about/guidelines.xhtml

A few times a week	2 (10%)
Once a day	3 (15%)
Several times a day	8 (40%)

Results

The key findings are outlined below. Example quotes are in Table 3.

Regular foot checking: Generally, participants perceived foot checking as acceptable and important for preventing DFUs. Many found foot checking easy to do and already checked their feet regularly. However, many participants reported physical limitations (e.g. limited mobility) and other physical barriers (e.g. wearing casts or bandages) that restricted foot checking. While some people found it easy to spot changes in foot health, others reported difficulties knowing what to look for and in judging whether any changes were problematic. A few described how it is easy to become lax over time, forgetting to check feet regularly or not thoroughly checking. Participants identified several facilitators to foot checking, including using a mirror to check feet, getting someone else to check, and integrating foot checking into everyday routine (e.g. when putting on socks).

When discussing the planned intervention features (e.g. foot checking reminders, facilities for note-taking), some people believed it would be useful to set up regular email foot checking reminders because it is easy to forget. Others felt reminders could be irritating or were unnecessary, as they, or their podiatrist, already regularly checked their feet. Generally, people thought it would be helpful to be able to make a note of any changes in their foot health to track changes in foot health over time. A few people felt this was unnecessary because they already checked their feet regularly, and knew what to look for, or believed it would be difficult to remember to note down changes.

Table 3 Table of key issues arising from our qualitative study and illustrative quotes

Issue arising from our qualitative study	Participant quotes
Foot checking	

For peer review only - http://bmjopen.bmj.com/site/about/guidelines.xhtml

Some participants had physical limitations that make it difficult to check their feet.	"As you get older you're not so mobile so you can't see right underneath [your foot], so it's a bit of guesswork until you do go…to [the] podiatrist" (P10, Male)
Some people found it difficult to know what to look for when foot checking and when to self-refer.	"Recognising them [DFUs] I think is the hardest part" (P14, Male)<br>"Sometimes…I go [to the podiatrist] and it's not an ulcer…but I can't tell" (P8, Male)
A few participants found it difficult to keep up foot checking long-term.	"You kind of become rather lax about perhaps doing it [foot checking] properly" (P1, Male)
There were mixed views on foot checking reminders.	"I don't think I would need to be reminded. I'm doing it [foot checking] already, really" (P3, Female)<br>"It's nice to have a reminder. Sometimes you get a bit complacent and you think 'Oh, I'll do it next time'" (P10, Male)
Rapid self-referral	
Some participants found it difficult to contact and get an appointment with their DFU team.	"Sometimes you can't get appointments…By the time you are seeing somebody it's either through A&E, because you've been rushed in 'cause your foot's swollen up and changed colour" (P18, Female)
Some participants expressed concerns about self-referring.	"If you do that [point out changes in foot health] every visit and it's nothing to worry about, you're paranoid, micromanaging. But if you don't mention something you've seen previously, you're complacent and don't care about your health. You can't win" (P18, Female)
Some participants found it difficult to know which health professional to contact when reporting DFUs.	"Who do you contact if you have a problem? Your own doctor? Or the nurse, diabetic nurse? Or the podiatrist?" (P5, Male)
Physical activity	
Some participants have physical limitations that make it difficult to engage in physical activity.	"I get very breathless. I don't walk much at all. I know I should, but I don't" (P3, Female)
Some participants also expressed concerns about physical activity causing another DFU.	"Even though you might not have an ulcer, even if you go back to minimal activity…you can still get that ulcer come back" (P18, Female)
Some participants found it can be difficult to keep up with physical activity over time.	"It is easy to find something else to do [instead of physical activity]. You've got to be pretty disciplined" (P6, Female)
There were mixed views on pedometers.	"The pedometer is a really good idea though…It's like a game – you want to make sure you can get as many steps in" (P20, Female)<br>"[The pedometer is] almost like being spied on" (P14, Male)
Emotional management	
Emotional management was relevant and valued by some participants, but not everyone.	"I'm one o' these anxiety merchants, me. I worry for the world…so it'd [emotional management] be very helpful" (P10, Male)<br>"I don't think personally I would have taken it [emotional management] on board at all…it's not gonna make any difference to me…I just think I've got it [DFUs], I've got to put up with it…I don't want to sit on a couch breathing in and out, I want to get on and do something" (P2, Female)
Delivery methods	
Participants were positive about the	"Personally think the website would be far better than the

For peer review only - http://bmjopen.bmj.com/site/about/guidelines.xhtml

idea of a website, but there were some concerns about computer literacy.	booklet…It's prodding me to do it [use the intervention]…If it's in a leaflet, it just gets left" (P14, Male, internet user)<br>"I love…anything interactive like that [the quiz in the example website] I think is great…you feel part of it [the intervention], rather than just being dictated to…[the information] tends to sink in better" (P20, Female, internet user)<br>"If I was competent…I would do it on the computer. But I'm not competent" (P8, Male, infrequent internet user)
A booklet might be helpful for quick reference and for those who do not use the internet.	"A booklet is always there, you can always refer to it, you've got something in black and white" (P8, Male)
Delivering the intervention via smartphone was less acceptable.	"Mobile phone - you've got all the problems of the computer, but on a smaller screen…a lot of diabetics [have] got problems with their eyes as well" (P17, Male)
Participants liked the idea of additional health professional support, but not for the intended purpose of supporting behaviour maintenance.	"It'd [additional health professional support] give me the confidence to know that 'well, I am alright with my foot as it is'…because you can get a bit paranoid over it [your foot health]" (P17, Male)<br>"They could give…one-to-one advice on…is there anything else that you could do…better than what I'm doing myself" (P3, Female)

Rapid self-referral in the event of changes in foot health: Most participants were positive about self-referral, viewing it as important. However, many people found it difficult to contact their DFU team. Long waiting times left some participants worried about how their foot health might decline in the meantime, which led one person to treat their feet themselves, instead of self-referring. In contrast, some participants reported the opposite and found it easy to get an appointment with their DFU team. A few participants were unsure which health professional to contact when reporting DFUs (e.g. podiatrist, diabetes nurse, GP). Some expressed concerns about looking foolish or wasting health professionals' time when self-referring for changes in foot health that turned out to be normal. One person had trouble with getting her concerns taken seriously and a few people worried about being a burden to health professionals. Some participants wanted reassurance from health professionals that it was right to have sought help.

Graded and regular physical activity: Most participants were positive about physical activity, stating that they would like to or were already doing it. People generally viewed

For peer review only - http://bmjopen.bmj.com/site/about/guidelines.xhtml

physical activity as important for general health and diabetes management. However, many participants reported physical limitations (e.g. pain, fatigue) or diabetic complications (e.g. neuropathy, residual damage to feet from previous DFUs) that made it difficult to be active. Participants reported that it was important to find the right activity to overcome their physical limitations, suggesting activities that did not put pressure on their feet, such as seated exercises. Some were concerned that physical activity might cause another DFU or exacerbate other health conditions.

Some participants stated that it could be difficult to maintain physical activity. A few mentioned that integrating physical activity into their daily routine (e.g. getting off the bus one stop early) and positive encouragement helped. Participants viewed self-monitoring, goal setting, and pedometers as helpful for maintaining motivation. However, some people disliked the idea of being 'spied on' or told what to do, expressed doubts about the accuracy of pedometers, or were unsure whether they would use them.

Emotional management: Over half of participants viewed emotional management positively and reported experiencing low mood, frustration, anger, and stress either during or after a DFU. Others had not experienced such emotions relating to their DFUs and viewed emotional management as irrelevant. A few people viewed emotional management negatively due to previous negative experiences. For example, some had experienced unhelpful reactions from doctors when discussing emotions, disliked talking about their feelings in counselling, or had received unhelpful information about emotional management (e.g. being given advice that did not consider their physical limitations). Some expressed a lack of understanding about how the emotional management would help or perceived it as contrary to their personal style of managing emotions (i.e. ignoring their problems, 'getting on with it').

For peer review only - http://bmjopen.bmj.com/site/about/guidelines.xhtml

Intervention delivery methods: Most participants were positive about the idea of the intervention being delivered via a booklet. Booklets were perceived as quick and easy to refer to, portable, and easily shared or distributed (e.g. with relatives or picked up from clinics). However, some participants commented that booklets were easily misplaced or forgotten. Most internet users reacted positively to the idea of a website, mainly because it was easy to access, convenient, and had interactive features (e.g. quizzes, email reminders). Nonetheless, non-users and a few infrequent internet users expressed concern about their own computer literacy. Some participants disliked reading on a computer screen and a few participants had concerns about security of web interventions. However, when participants were shown the example website, they generally viewed it positively, stating that it looked easy to use. A few participants would have liked to access the intervention using a computer tablet as they already used one or knew people who did. Most viewed delivery using a smartphone negatively because of their limited use of phones or difficulties with using small screens due to poor eyesight (caused by diabetes). A few participants commented that it might be helpful to deliver the intervention through multiple modes (booklet, website, tablet, or phone).

Generally, participants were in favour of additional health professional support. However, they interpreted this as support to gain reassurance about the status of their foot health, and advice on foot care or when to self-refer (which would be covered in the website/booklet), rather than support to raise motivation for engaging with the target behaviours. Very few participants said they might use this support to answer questions about information in the booklet or website.

Explanations of how the evidence from the scoping review and qualitative study informed intervention planning are provided in the next sections on Guiding Principles and Behavioural Analysis.

For peer review only - http://bmjopen.bmj.com/site/about/guidelines.xhtml

Creating the intervention plan

Creating guiding principles

Purpose

In line with the person-based approach,[26] brief guiding principles are developed and consulted throughout intervention development to ensure that the intervention is underpinned by a coherent focus.

Methods

Drawing upon the findings from our scoping review and qualitative study, key characteristics of target users and the key behavioural issues, needs and challenges the intervention must address were described. From this, guiding principles were created, which outline the intervention design objectives that will address these key behavioural issues, needs and challenges, and the key intervention features designed to achieve these objectives. The multidisciplinary team decided on the key features based on their ability to address the intervention objectives.

Results

People who have had DFUs can feel they have little or no control over preventing DFUs, as DFUs can occur even when people are engaging in foot care behaviours. This leaves people feeling hopeless and frustrated.[17] Some people may feel self-blame or guilt for not engaging in foot care behaviours, especially in the event of reoccurrence.[17] Therefore, one design objective was to reduce feelings of hopelessness, frustration, self-blame, and guilt following a DFU.

People may be uncertain about the signs of a DFU and when to seek help from a health professional.[30] Our qualitative study highlighted that some people were concerned about

For peer review only - http://bmjopen.bmj.com/site/about/guidelines.xhtml

looking foolish, being a burden, or wasting healthcare professionals' time if changes in their feet turn out to be normal. This may delay help seeking. Therefore, one design objective was to build patients' confidence in making a self-referral.

This population are likely to have physical limitations and/or co-morbidities. Our qualitative study highlighted that these challenges may make it difficult for people to engage in foot checking and physical activity. They may also be reluctant to increase activity in case it causes re-ulceration. Thus, one design objective was to acknowledge that patients may have physical limitations that make it difficult to engage in foot checking and physical activity.

Our scoping review highlighted that people may experience difficult emotions following a DFU.[17,31–34] However, some participants in our qualitative research did not experience such emotions and, therefore, did not perceive emotional management as useful. Therefore, one design objective was to acknowledge that emotional management may not be relevant for all patients.

As the physical activity and emotional management content was not relevant to all patients, these components were made optional, rather than mandatory, to avoid discouraging patients from engaging in the other target behaviours if they do not want to increase physical activity or engage in emotional management.

In our qualitative study, many reacted positively to the idea of a web-based intervention, but some participants expressed concerns about their computer literacy. These concerns were also evident in the literature.[32] Therefore, one design objective was to ensure people feel confident in using the maintenance intervention. We decided to deliver the intervention using a website and provide key information and advice in a booklet for quick reference and for non-internet users. At the preceding initiation phase, health professionals will address

For peer review only - http://bmjopen.bmj.com/site/about/guidelines.xhtml

concerns, and speak favourably of the digital intervention to encourage use. Table 4 details the REDUCE maintenance intervention guiding principles.

For peer review only

For peer review only - http://bmjopen.bmj.com/site/about/guidelines.xhtml

Table 4 The guiding principles for the development of the REDUCE maintenance intervention

Intervention design objectives	Key features
To reduce feelings of hopelessness, frustration, self-blame, and guilt following a DFU	• Emphasise target behaviours that patients can engage in to reduce their chances of getting another DFU, while acknowledging that there are precipitating factors (e.g. increased age, neuropathy, foot shape) that are out of their control.<br>• Enhance patients' confidence in the target behaviours (e.g. by providing a rationale for the necessity of the target behaviours, scientific evidence that behaviours are effective, patient stories, and a quiz on the benefits of the behaviours).<br>• Validate patients' feelings of frustration and hopelessness if a DFU does reoccur and avoid arguments that may be viewed as blaming patients for this re-occurrence.<br>• Provide links to emotional management techniques that can help people to manage difficult emotions.
To build patients' confidence in making a self-referral	• Provide links to foot checking training (e.g. by providing information and photographs on what DFUs look like, what signs to look out for, and how often feet should be checked with guided practice).<br>• Provide reassurance that self-referral is necessary (e.g. through a foot health checklist that provides personalised feedback on whether or not patients should self-refer, based on their symptoms).<br>• Address concerns around looking foolish or wasting the DFU team's time when self-referring (e.g. a) emphasise that the DFU team would rather they were contacted early so they are better able to treat any DFUs, b) provide patient stories about how other patients overcame feelings of burden).
To acknowledge that patients may have physical limitations that make it difficult to engage in foot checking and physical activity	• Provide guidance on how to check your feet if you have physical limitations, including using a mirror to check the bottom of your feet and asking someone else to check for you.<br>• Make intervention content on physical activity optional.<br>• Provide guidance about a variety of safe and low impact physical activities to enable patients to find an activity that is suitable for them.<br>• Address physical activity concerns all the way through the intervention (i.e. in the maintenance intervention and prior initiation phase) (e.g. by providing information about the safety of physical activity, patient stories about how other patients overcame these barriers).
To acknowledge that emotional management may not be relevant for all patients	• Make intervention content on emotional management optional.<br>• Emphasise that some people, but not everyone, might experience difficult emotions following a DFU to avoid excluding those who may not relate to this content.<br>• Provide a variety of brief emotional management techniques (e.g. CBT, mindfulness techniques) to allow each person to find a technique that fits with their own personal style of managing emotions.
To ensure patients feel confident in using the maintenance intervention	• Keep website navigation simple and follow guidelines for maximising website usability.<br>• Health professionals at the prior initiation phase will provide technical support, address self-doubts, and

For peer review only - http://bmjopen.bmj.com/site/about/guidelines.xhtml

Intervention design objectives	Key features
	speak favourably of the digital intervention to encourage use.<br>• Encourage friends and family to assist people with website use, if appropriate.<br>• Provide a booklet for quick reference and for those who do not have access to the internet.

For peer review only

For peer review only - http://bmjopen.bmj.com/site/about/guidelines.xhtml

Behavioural analysis

Purpose

To use behaviour change theory to systematically describe the maintenance intervention content, identify potential determinants of behaviour (i.e. what needs to change for a behaviour to occur), and map it onto the evidence derived from our scoping review, our qualitative study, and expert consultation.

Methods

Behavioural analysis involves comprehensively mapping out the elements of an intervention, linking the evidence-base to behaviour change theory and the intervention components. Providing a clear description of the intervention is essential for replication in research and practice, data extraction in systematic reviews, and process evaluation planning.[21,24,25] The Behaviour Change Wheel (BCW [37,38]) and Behaviour Change Techniques Taxonomy (BCTv1 [39]) were developed to standardise the classification and description of complex interventions and help identify an intervention's 'active ingredients' and behavioural determinants. Such standardisation provides a common language to avoid any confusion that may occur when different terminology are used for the same intervention technique or different techniques are referred to using the same terminology.[40] The BCW draws upon the COM-B model, which argues that behaviour is influenced by an individual's Capability, Opportunity, and Motivation to change behaviour.[38]

In addition to the four target behaviours identified from the outset, the behavioural analysis also identified one subsidiary behaviour (engaging with the digital MI) that is necessary to enact these target behaviours. Barriers and facilitators for each behaviour were identified from the primary qualitative research, scoping review, and expert opinion from the multidisciplinary project team. Intervention components that addressed each barrier and facilitator were selected.

For peer review only - http://bmjopen.bmj.com/site/about/guidelines.xhtml

These components are reported using patient-centred, autonomy-supportive language to emphasise the importance of delivering these components in a way that will enhance intrinsic motivation and ensure a positive intervention experience.[26] The intervention components were coded using the BCTv1 and mapped onto the BCW to identify their corresponding intervention function (ways an intervention can change behaviour, e.g. 'education'), and target construct (what needs to change for the behaviour to occur, e.g. 'psychological capability'). The BCTv1 and BCW were then examined to check for potentially useful additional intervention functions, target constructs, or behaviour change techniques.

Results

The behavioural analysis is presented in Appendix 2. The maintenance intervention will target all six behavioural sources included in the BCW (physical and psychological capability, reflective and automatic motivation, and physical and social opportunity), and employ six different BCW intervention functions (education, persuasion, modelling, training, enablement, environmental restructuring) using 18 different BCTs. Intervention components that received a mixed reaction from our qualitative research participants (i.e. foot checking reminders, pedometers) were made optional to promote patient autonomy.

Although participants would have liked additional health professional support, the support participants wanted was more clinical in nature (e.g. advice about foot health or when to self-refer). As such support would be provided in the website/booklet, this form of health professional support was deemed superfluous. Therefore, additional health professional support was not included in the intervention plan. One issue that arose from our qualitative study could only be addressed to a limited degree by the maintenance intervention, namely the difficulties people experienced contacting, and getting an appointment, with their DFU team. This will be addressed by educating patients about the national guidelines and local procedures for self-referrals, and how to communicate the reason for self-referral to their

For peer review only - http://bmjopen.bmj.com/site/about/guidelines.xhtml

DFU team. However, improving local self-referral pathways or modifying health professionals' behaviour is outside of the scope of this intervention.

Logic modelling

Purpose

To model the hypothesised mechanisms of action of the maintenance intervention (i.e. how it is thought to work).[25,28,29]

Methods

The logic model draws together findings from the scoping review, qualitative study, and behavioural analysis into a testable model that outlines how the different intervention components are hypothesised to impact on subsequent components and ultimately affect outcomes.

Results

The logic model (Figure 1) can be broken down into three major components.

Intervention techniques and processes: The intervention techniques summarise the behaviour change techniques outlined in the behavioural analysis and the seven processes they are hypothesised to affect: skills, self-efficacy, knowledge, positive outcome expectancies, sense of personal control, social support and physical opportunity. These are the psychosocial factors that need to be modified for the intervention's target behaviours to change and were identified through the behavioural analysis.

Each set of intervention techniques is hypothesised to mainly affect one of these processes, which subsequently affect one or more of the intervention's target behaviours. They are organised in order of importance, with more integral processes that were consistently

For peer review only

identified as key in the scoping review and qualitative study at the top and less integral processes at the bottom (e.g. optional features).

Purported mediators: Purported mediators are the target behaviours of the intervention that are hypothesised to directly affect DFUs in the long-term. These behaviours are divided into 'core behaviours' that are hypothesised to be most important in determining DFU outcomes (foot checking, rapid self-referral), and 'optional behaviours' that are only relevant for some patients (physical activity, emotional management). These behaviours' may impact either directly, as in the case of physical activity, or indirectly, via their effect on the other target behaviours, as is the case in emotional management. Emotional management is hypothesised to have an indirect effect on the other behaviours due to the negative effects that low mood (or negative thoughts) can have on behavioural engagement.

Outcomes: The logic model specifies three outcomes that the intervention is ultimately trying to change, the primary outcome of interest (ulcer free survival with limbs intact), and two interim outcomes that may be affected by the target behaviours and may, directly or indirectly, affect the primary outcome (severity of DFU at presentation and time taken for DFU healing in the event of a recurrence).

DISCUSSION

This paper describes the use of theory-, evidence- and person-based approaches [28] to developing an intervention plan for the REDUCE maintenance intervention, an intervention that aims to reduce re-ulceration risk by supporting patients to maintain behaviour change and emotional management. These different approaches provided complementary insights into how the intervention could be designed to maximise its acceptability, feasibility, and effectiveness. For example, the scoping review highlighted that patients experience difficult emotions following DFUs,[17,31–34], however, the qualitative interviews suggested that this

For peer review only - http://bmjopen.bmj.com/site/about/guidelines.xhtml

was only relevant for some patients, suggesting that this content should be made optional. In line with person- and evidence-based approaches, our scoping review and qualitative study deepened our understanding of the psychological and behavioural needs of people who have had DFUs and highlighted several barriers and facilitators to the intervention's target behaviours, some of which had been highlighted in the literature (e.g. lack of knowledge regarding what to look for when foot checking [17,30]) and some which had received little prior attention (e.g. lack of knowledge about when to self-refer). It also highlighted important advantages of, and barriers to, successful use of different intervention delivery methods (e.g. lack of confidence in ability to use digital interventions). Our qualitative study updated prior research published over a decade ago that highlighted concerns regarding limited computer access and poor computer skills among people at risk of DFUs.[32] Our guiding principles succinctly summarised the distinctive design objectives and features of the maintenance intervention, while our behavioural analysis and logic modelling comprehensively described the intervention and its potential mechanisms of action.

This is the first paper to use this methodology to provide a comprehensive plan of a DFU intervention. Transparent reporting of the intervention planning process will allow other researchers to easily understand how this methodology could be applied to different intervention contexts and facilitate comparison between different interventions.[12,23–25] The use of primary qualitative research allowed us to understand patients' views on the delivery methods for behaviour change interventions and three behaviours that have received little attention in the DFU literature to date: engaging in rapid self-referral, graded and regular physical activity, and emotional management. For example, participants had mixed reactions to some behaviours (i.e. physical activity and emotional management) and design features (e.g. email reminders), which were subsequently made optional. Participants also reported experiencing difficulties with accessing their DFU team when self-referring. Future

For peer review only - http://bmjopen.bmj.com/site/about/guidelines.xhtml

research should further explore and address any professional and organisational barriers to self-referral.

The qualitative research used purposive sampling which enabled us to explore the acceptability and feasibility of a digital intervention across a diverse set of people, including those who were frequent and infrequent internet users. Although the sample was representative of the population of people with DFUs (who tend to be older [14] and may therefore be retired), it would be helpful to explore the views of younger and employed people, as they may report different barriers to behaviour change. The rapid scoping review allowed scientific evidence to be quickly incorporated into the intervention plan, but it was not systematic, so it is possible that some literature was missed.

Recent NICE guidelines for the prevention and management of diabetic foot problems [2] identified a need to develop and evaluate new interventions targeting psychological and behavioural factors. Our research has provided a plan for such an intervention, as well as identified potential barriers to behaviour change and behaviour change techniques that are likely to be useful within clinical practice. In future work, we intend to use this intervention plan to develop the maintenance intervention and then conduct an effectiveness trial to evaluate the effectiveness and cost-effectiveness of the entire REDUCE intervention, whilst also examining if the intervention works as hypothesised.

ACKNOWLEDGMENTS

The research team acknowledges the support of the National Institute of Health Research Clinical Research Network (NIHR CRN). We are grateful to the members of the REDUCE PPI panel for their valuable input on the study materials and interview schedule, and the participants who took part in this research.

COMPETING INTERESTS

For peer review only - http://bmjopen.bmj.com/site/about/guidelines.xhtml

All authors have completed the ICMJE uniform disclosure form at www.icmje.org/coi_disclosure.pdf and declare: KG, KS, KV, LY, FG, TC, ND, GR and KB had financial support from NIHR for the submitted work; TC had other support from NIHR; no financial relationships with any organisations that might have an interest in the submitted work in the previous three years; and no other relationships or activities that could appear to have influenced the submitted work.

FUNDING

This article summarises independent research funded by the National Institute for Health Research (NIHR) under its Programme Development Grants Programme (Grant Reference Number RP-DG-0615-10005). The views expressed are those of the author(s) and not necessarily those of the NHS, the NIHR or the Department of Health.

CONTRIBUTIONS

KGre, KS, KV, LY, FG, TC, GR, ND, KGra, JW and KB designed the study. KG, KS, KB, and LY led the intervention planning, with input from the other co-authors. KGre and KS were responsible for recruitment, carrying out the interviews, and analysing the data, with support from KB. KGre and KS jointly drafted the manuscript with initial support from KB and LY. KGre, KS, KV, LY, FG, TC, GR, ND, KGra, JW and KB critically reviewed the manuscript, contributing important intellectual content, and approved the final manuscript.

DATA SHARING STATEMENT

No additional data are available.

REFERENCES

1 Singh N, Armstrong DG, Lipsky BA. Preventing foot ulcers. *J Am Med Assoc* 2005;293:217–28. doi:10.1001/jama.293.2.217

For peer review only - http://bmjopen.bmj.com/site/about/guidelines.xhtml

2 NICE. Diabetic foot problems: prevention and management. 2015

3 Kerr M. Improving footcare for people with diabetes and saving money: an economic study in England. 2017

4 Margolis D, Kantor J, Berlin J. Healing of diabetic neuropathic foot ulcers receiving standard treatment. *Diabetes Care* 1999;22:692–5.

5 Boulton AJM. The diabetic foot: Grand overview, epidemiology and pathogenesis. *Diabetes Metab Res Rev* 2008;24:S3–6. doi:10.1002/dmrr.833

6 Armstrong DG, Boulton AJM, Bus SA. Diabetic foot ulcers and their recurrence. *N Engl J Med* 2017;376:2367–75. doi:10.1056/NEJMra1615439

7 Ismail K, Winkley K, Stahl D, *et al.* A cohort study of people with diabetes and their first foot ulcer. *Diabetes Care* 2007;30:1473–9.

8 van Netten JJ, Price PE, Lavery LA, *et al.* Prevention of foot ulcers in the at-risk patient with diabetes: a systematic review. *Diabetes Metab Res Rev* 2016;32:84–98. doi:10.1002/dmrr.2701

9 Mason J, O'Keeffe C, McIntosh A, *et al.* A systematic review of foot ulcer in patients with Type 2 diabetes mellitus. I: prevention. *Diabet Med* 1999;16:801–12. doi:10.1046/j.1464-5491.1999.00133.x

10 Dorresteijn J, Kriegsman D, Assendelft W, *et al.* Patient Education for Preventing Diabetic Foot Ulceration. *Cochrane Database Syst Rev* Published Online First: 2012. doi:10.1002/14651858.CD001488.pub2

11 Hunt DL. Diabetes: foot ulcers and amputations. *BMJ Clin Evid* 2011;2011:1–44.

12 Hoogeveen RC, Dorresteijn JA, Kriegsman DM, *et al.* Complex interventions for

For peer review only - http://bmjopen.bmj.com/site/about/guidelines.xhtml

preventing diabetic foot ulceration. *Cochrane Database Syst Rev* 2015;8:1–42. doi:10.1002/14651858.CD007610.pub3

13 Vileikyte L. Psychosocial and behavioral aspects of diabetic foot lesions. *Curr Diab Rep* 2008;8:119–25. doi:10.1007/s11892-008-0022-1

14 Clincal Audit and Registries Management Service. National diabetes foot care audit report 2014-2015. 2016 doi:9781783866762

15 Armstrong DG, Lavery LA, Holtz-Neiderer K, *et al.* Variability in activity may precede diabetic foot ulceration. *Diabetes Care* 2004;27:1980–4.

16 Lemaster JW, Mueller MJ, Reiber GE, *et al.* Effect of weight-bearing activity on foot ulcer incidence in people with diabetic peripheral neuropathy: feet first randomized controlled trial. *Phys Ther* 2008;88:1385–98. doi:10.2522/ptj.20080019

17 Beattie AM, Campbell R, Vedhara K. 'What ever I do it's a lost cause.' The emotional and behavioural experiences of individuals who are ulcer free living with the threat of developing further diabetic foot ulcers: a qualitative interview study. *Heal Expect* 2014;17:429–39. doi:10.1111/j.1369-7625.2012.00768.x

18 Winkley K, Stahl D, Chalder T, *et al.* Risk factors associated with adverse outcomes in a population-based prospective cohort study of people with their first diabetic foot ulcer. *J Diabetes Complications* 2007;21:341–9. doi:10.1016/j.jdiacomp.2007.09.004

19 Monami M, Longo R, Desideri CM, *et al.* The diabetic person beyond a foot ulcer: healing, recurrence, and depressive symptoms. *J Am Podiatr Med Assoc* 2008;98:130–6. doi:98/2/130 [pii]

20 Vedhara K, Miles JN V, Wetherell MA, *et al.* Coping style and depression influence the healing of diabetic foot ulcers: observational and mechanistic evidence.

For peer review only - http://bmjopen.bmj.com/site/about/guidelines.xhtml

Diabetologia 2010;53:1590–8. doi:10.1007/s00125-010-1743-7

21 Craig P, Dieppe P, Macintyre S, *et al.* Developing and evaluating complex interventions: New guidance. 2008.

22 Vedhara K, Beattie A, Metcalfe C, *et al.* Development and preliminary evaluation of a psychosocial intervention for modifying psychosocial risk factors associated with foot re-ulceration in diabetes. *Behav Res Ther* 2012;50:323–32. doi:10.1016/j.brat.2012.02.013

23 Michie S, Abraham C. Advancing the science of behaviour change: a plea for scientific reporting. *Addiction* 2008;103:1409–10. doi:10.1111/j.1360-0443.2008.02291.x

24 Michie S, Abraham C. Interventions to change health behaviours: evidence-based or evidence-inspired? *Psychol Health* 2004;19:29–49. doi:10.1080/0887044031000141199

25 Moore G, Audrey S, Barker M, *et al.* Process evaluation of complex interventions. UK Medical Research Council (MRC) guidance. 2012.

26 Yardley L, Morrison L, Bradbury K, *et al.* The person-based approach to intervention development: application to digital health-related behavior change interventions. *J Med Internet Res* 2015;17:e30. doi:10.2196/jmir.4055

27 Kok G, Schaalma H. Using theory in psychological interventions. In: Michie S, Abraham C, eds. Health Psychology in Practice. 2004:203–29. doi:10.1002/9780470694008.ch11

28 Band R, Bradbury K, Morton K, *et al.* Intervention planning for a digital intervention for self-management of hypertension: a theory-, evidence- and person-based approach.

For peer review only - http://bmjopen.bmj.com/site/about/guidelines.xhtml

Implement Sci 2017;12:25. doi:10.1186/s13012-017-0553-4

29 Baxter SK, Blank L, Woods HB, *et al.* Using logic model methods in systematic review synthesis: describing complex pathways in referral management interventions. *BMC Med Res Methodol* 2014;14:62. doi:10.1186/1471-2288-14-62

30 Searle A, Gale L, Campbell R, *et al.* Reducing the burden of chronic wounds: prevention and management of the diabetic foot in the context of clinical guidelines. *J Health Serv Res Policy* 2008;13 Suppl 3:82–91. doi:10.1258/jhsrp.2008.008011

31 Hjelm K, Nyberg P, Apelqvist J. Gender influences beliefs about health and illness in diabetic subjects with severe foot lesions. *J Adv Nurs* 2002;40:673–84. doi:10.1046/j.1365-2648.2002.02427.x

32 Burnside J, Wells W, Smith M, *et al.* Education for the prevention of foot ulcers: views of those with neuropathy. 2006.

33 Kinmond K, McGee P, Gough S, *et al.* 'Loss of self': a psychosocial study of the quality of life of adults with diabetic ulceration. *World Wide Wounds* 2003;13:6–16. doi:10.1016/S0965-206X(03)80025-6

34 Searle A, Campbell R, Tallon D, *et al.* A qualitative approach to understanding the experience of ulceration and healing in the diabetic foot: Patient and podiatrist perspective. *Wounds* 2005;66:1–12.

35 Pollock RD, Unwin NC, Connolly V. Knowledge and practice of foot care in people with diabetes. *Diabetes Res Clin Pract* 2004;64:117–22. doi:10.1016/j.diabres.2003.10.014

36 Rowsell A, Muller I, Murray E, *et al.* Views of people with high and low levels of health literacy about a digital intervention to promote physical activity for diabetes: A

For peer review only - http://bmjopen.bmj.com/site/about/guidelines.xhtml

qualitative study in five countries. *J Med Internet Res* 2015;17:e230. doi:10.2196/jmir.4999

37 Michie S, Atkins L, West R. *The Behaviour Change Wheel: A Guide to Designing Interventions*. London: Silverback Publishing 2014

38 Michie S, van Stralen MM, West R. The behaviour change wheel: a new method for characterising and designing behaviour change interventions. *Implement Sci* 2011;6:42. doi:10.1186/1748-5908-6-42

39 Michie S, Richardson M, Johnston M, *et al.* The behavior change technique taxonomy (v1) of 93 hierarchically clustered techniques: building an international consensus for the reporting of behavior change interventions. *Ann Behav Med* 2013;46:81–95. doi:10.1007/s12160-013-9486-6

40 Michie S, Wood CE, Johnston M, *et al*. Behaviour change techniques: the development and evaluation of a taxonomic method for reporting and describing behaviour change interventions (a suite of five studies involving consensus methods, randomised controlled trials and analysis of qualitative data). *Health Technol Assess* 2015;19:1–188. doi:10.3310/hta19990

FIGURE LEGEND

Figure 1 REDUCE Maintenance Intervention Logic Model

SUPPLEMENTARY MATERIAL

Appendix 1: Interview schedule and prompt cards

Appendix 2: Behavioural analysis table

For peer review only - http://bmjopen.bmj.com/site/about/guidelines.xhtml

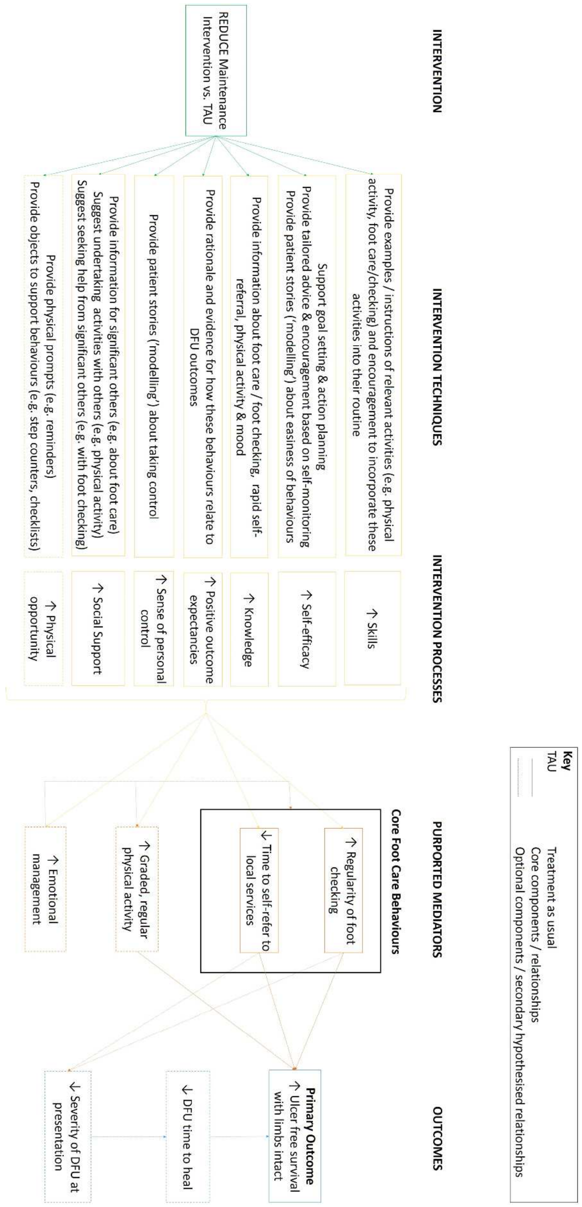

Figure 1 REDUCE Maintenance Intervention Logic Model

119x248mm (600 x 600 DPI)

APPENDIX 1: INTERVIEW SCHEDULE AND PROMPT CARDS

Interview schedule

Section 1: Context

Q1. Can you tell me a bit about what it has been like for you to have a foot ulcer?
Q2. Can you tell me about anything that you do to look after your feet currently?

Section 2: Acceptability and feasibility of the maintenance intervention

[Provide explanation of initiation phase and maintenance intervention]

1. Content of website/booklet

Card 1 (foot checking):

Q3. What do you think about these ideas? What do you like about these ideas? What do you dislike about these ideas?
Q4. The foot checker will ask you to check your feet every day for foot damage. What things might make it difficult for you to do this? Do you have any concerns about checking your feet every day?

Card 2 (help-seeking):

Q5. What do you think about these ideas? What do you like about these ideas? What do you dislike about these ideas?
Q6. The website may ask you to contact your diabetes team if you are concerned about any foot damage you have. What things might make it difficult for you to do this? Do you have any concerns about doing this?

Card 3 (physical activity):

Q7. What do you think about these ideas? What do you like about these ideas? What do you dislike about these ideas?
Q8. What things might make it difficult for you to get more active? Do you have any concerns about getting more active?

Card 4 (dealing with feelings):

Q9. What do you think about these ideas? What do you like about these ideas? What do you dislike about these ideas? Is there anything you do at the moment that helps you when you feel stressed / low?

Additional content:

Q10. What other things could we do to help you to look after your feet?

2. Delivery formats

Q11. What are your thoughts about the long-term support for maintaining habits being provided in **a booklet**? What do you like about this idea? What do you dislike about this idea?

For peer review only

Q12. What are your thoughts about the long-term support for maintaining habits being provided through **a website**? What do you like about this idea? What do you dislike about this idea?

Q13. If we were to deliver the programme through a website, what do you think about the idea of using **a computer or tablet**, such as an iPad, to access the website?

Q14. What do you think about the idea of using **a mobile phone** to access the website?

Card 5 (optional health professional support):

Q15. What do you think about this idea? What do you like about this idea? What do you dislike about this idea?

Q16. [If optional support would be helpful] What would you like to talk to the health professional about? Why would this be helpful?

Q17. You could contact the health professional in person, over the phone, and by email. Which one of these options would you prefer? Why?

Prompt Cards

Card 1 – Check your feet regularly

Why is this important?

It can be difficult to know when you might be developing a foot ulcer because some of the initial signs can be very small and hard to spot. It is important to examine your feet regularly so you are better able to spot any changes in your feet.

The website or booklet will:

- Ask you to check your feet every day
- Allow you to make a note of any changes in your feet

Set up regular reminders to check your feet which the foot checker can send to you by email or text messages to your mobile phone

Card 2 – What to do if you spot any foot damage

Why is this important?

It is important to report any changes in your feet to your diabetes team as soon as possible. This will allow them to check your feet and see if you need any treatment. The quicker your feet are treated, the more likely that any damage to your feet will heal.

The website or booklet will:

- Give you personalised advice on what to do if you spot any changes in your feet
- Advise you when you may need to contact your diabetes team

For peer review only

Card 3 – Getting active

Why is this important?

When you don't have a foot ulcer, it is safe and important to be mobile and active. This is because being active will improve your circulation and blood sugar, and reduce your chances of getting another ulcer. It is important that you pace your activity – little and often is best. Irregular activity (doing nothing and then doing too much) can be harmful because it increases the chances of injuring your feet.

The website or booklet will:

- Help you to slowly increase physical activity, such as using an exercise bike, seated exercise, walking, or any other things you like to do to be active.
- Set weekly physical activity goals, for example, going for a walk once or a few times a week.
- Ask you to enter in information about how you got on with your goals each week and provide you with personalised advice based on your progress.

A free step counter (or pedometer) that clips onto your belt and counts how many steps you take. You can use this to set yourself daily or weekly step goals if you would like to.

Card 4 – Dealing with your feelings when you get another ulcer

Why is this important?

We know that people who have had an ulcer can feel frustrated and cross if, and when, they get another one. Some people can feel down or stressed at this time and it can be difficult to look after yourself when you are feeling this way. Dealing with your feelings can make you feel better and will make sure you put you and your health first.

The website or booklet will:

- Teach you techniques that have been shown to help improve mood and reduce stress.
- Many people have found these techniques to be helpful.

Card 5 - Optional health professional support:

The website or booklet will also:

- Give people the option to contact a diabetes trained health professional if they wanted to.
- This contact could be in person, over the phone, or by email.

For peer review only - http://bmjopen.bmj.com/site/about/guidelines.xhtml

APPENDIX 2: BEHAVIOURAL ANALYSIS OF REDUCE MAINTENANCE INTERVENTION USING THE BEHAVIOUR CHANGE WHEEL (BCW) AND BEHAVIOUR CHANGE TECHNIQUES TAXONOMY (BCTv1)

Key: DFU = Diabetic Foot Ulcer; EO = Barrier emerged from expert opinion; LR = Barrier emerged from literature review; QR = Barrier emerged from qualitative primary research; N/A = theoretical mapping not applicable; * = intervention components and BCTs identified through examination of the BCTv1 and BCW to check for additional intervention functions, target constructs, or behaviour change techniques.

**Barriers/*facilitator* to target behaviour**	**Intervention components**	**Target construct (BCW)**	**Intervention function (BCW)**	**Behaviour Change Technique (using BCTv1)**
**Target behaviour: Engaging in regular foot checking**				
Belief that foot checking will <u>do little</u> to delay getting a DFU [EO; LR]	• Provide a rationale for the necessity of regular foot checking, including evidence that it is effective for delaying DFUs	Psychological capability; Reflective motivation	Education; Persuasion	5.1 Information about health consequences<br>5.6 Information about emotional consequences
	• Provide patient stories demonstrating how regular foot checking helped other patients to take control of their DFUs	Psychological capability; Reflective motivation; Social opportunity	Education; Persuasion; Modelling	5.1 Information about health consequences<br>6.2 Social comparison<br>6.3 Information about others' approval<br>9.1 Credible source
Lack of knowledge regarding DFUs and foot checking procedures/ Lack of confidence in ability to check feet [LR, QR]	• Provide information and pictures on what DFUs look like, what signs of DFUs to look out for, and how to check their feet	Physical capability; Psychological capability	Education; Training	4.1 Instructions on how to perform the behaviour<br>5.1 Information about health consequences
	• Provide an online and printable foot health checklist so patients can spot changes in their foot health<br>• Allow patients to record any changes in their foot health	Psychological capability; Physical opportunity	Training; Environmental structuring	4.1 Instructions on how to perform the behaviour<br>12.5 Adding objects to the environment
	• Provide patient stories demonstrating how easy it was for other patients to engage in regular foot checking	Reflective motivation; Social opportunity	Persuasion; Modelling	6.2 Social comparison<br>6.3 Information about others' approval<br>9.1 Credible source
Forgetting to check your feet [EO, QR]<br><br>*Reminders to check your feet [EO,QR]*	• Allow patients to set up regular reminders to check your feet daily by email or text messages and decide on the frequency of these reminders	Physical opportunity	Environmental structuring	7.1 Prompts/cues

For peer review only - http://bmjopen.bmj.com/site/about/guidelines.xhtml

**Barriers/*facilitator* to target behaviour**	**Intervention components**	**Target construct (BCW)**	**Intervention function (BCW)**	**Behaviour Change Technique (using BCTv1)**
Difficulties engaging in foot checking in the long-term [LR, QR]<br><br>*Integrating foot checking into your routine [QR]*	• Allow patients to set their own daily foot checking goals<br>• Encourage patients to make a foot checking action plan	Reflective motivation	Enablement	1.1 Goal setting (behaviour)<br>1.4 Action planning<br>1.5 Review behaviour goal(s)
	• Advise patients to regularly practice foot checking in the same context (e.g. after showering, when putting socks on)	Psychological capability; Automatic motivation	Training; Enablement	8.1 Behavioural practice/rehearsal<br>8.3 Habit formation
Physical limitations, deformities and barriers (e.g. limited mobility, foot deformities, poor eyesight) [LR, QR]<br><br>*Using a mirror [QR]; Getting someone to check your feet for you [QR]*	• Provide guidance on how to check your feet if you have physical limitations, including using a mirror to check the bottom of your feet and asking someone else to check for you<br>• Provide information on the signs of DFUs and foot checking procedures for significant others who are helping with foot checking	Physical capability; Psychological capability; Social opportunity	Training; Education; Enablement	3.2 Social support (practical)<br>4.1 Instructions on how to perform the behaviour
**Target behaviour: Engaging in rapid self-referral in the event of changes in foot health**				
Belief that self-referral will <u>do little</u> to aid DFU healing [EO, LR] /Lack of confidence in DFU team [QR]	• Provide a rationale for the necessity of reporting any signs of foot damage, including evidence that this is effective for DFU healing	Psychological capability; Reflective motivation	Education; Persuasion	5.1 Information about health consequences<br>5.6 Information about emotional consequences
	• Provide patient stories demonstrating how rapid self-referral helped other patients to take control of their DFUs, and how they overcame previous frustrations with the DFU team and feelings that it was not worth it	Psychological capability; Reflective motivation; Social opportunity	Education; Persuasion; Modelling	5.1 Information about health consequences<br>6.2 Social comparison<br>6.3 Information about others' approval<br>9.1 Credible source
Lack of understanding regarding when to seek help and who to contact [EO, QR]	• Provide advice on when you may need to contact your diabetes team<br>• Advise patients to find out the contact details of their DFU team	Psychological capability	Education	4.1 Instructions on how to perform the behaviour

For peer review only - http://bmjopen.bmj.com/site/about/guidelines.xhtml

**Barriers/*facilitator* to target behaviour**	**Intervention components**	**Target construct (BCW)**	**Intervention function (BCW)**	**Behaviour Change Technique (using BCTv1)**
Difficulty accessing the DFU team and getting a quick appointment [EO, QR]	• Provide information on the national guidelines regarding timeline for referrals to DFU team<br>• Invite patients to refer to their local procedure for self-referrals given in their REDUCE action plan in the initiation phase<br>• Provide advice on how to communicate the reason for self-referral when contacting the DFU team	Psychological capability	Education	4.1 Instructions on how to perform the behaviour
Forgetting the contact details for the foot care team [EO]	• Invite patients to record the contact details of their foot care team and print this record to act as a reminder	Physical opportunity	Environmental structuring	7.1 Prompts/cues<br>12.5 Adding objects to the environment
Forgetting to contact their foot care team [EO]	• Invite patients to set up reminders to contact their foot care team if they record any signs of foot damage into the maintenance intervention and decide on the frequency of these reminders	Physical opportunity	Environmental structuring	7.1 Prompts/cues
Concerns about looking foolish or wasting the DFU team's time when reporting changes in foot health that turn out to be normal/ Not wanting to bother the DFU team [EO, QR]	• Reassure patients that health professionals would rather they were contacted early so they are better able to treat the DFU<br>• Provide patient stories on how other patients overcame feelings of being a burden<br>• Provide personalised feedback on whether or not they should self-refer, based on the answers they give to the foot health checklist	Psychological capability; Reflective motivation	Education; Persuasion	4.1 Instructions on how to perform the behaviour<br>6.3 Information about others' approval
**Target behaviour: Engaging in graded and regular physical activity**				
Belief that physical activity will do little to delay getting a DFU [EO, LR]<br><br>*Awareness of non-DFU related benefits of physical activity that are immediate and salient [EO]*	• Provide a rationale for the necessity of graded and regular physical activity and evidence that it is effective for delaying DFUs<br>• Provide a quiz about the benefits of physical activity for delaying DFUs, including other physical and mental benefits (e.g. improved sleep and energy, alleviation from aches and pains)	Psychological capability; Reflective motivation	Education; Persuasion	5.1 Information about health consequences<br>5.6 Information about emotional consequences
	• Provide patient stories demonstrating how graded and regular physical activity helped other patients to take control of their DFUs and led to other salient benefits	Reflective motivation; Social opportunity	Education; Persuasion; Modelling	5.1 Information about health consequences<br>6.2 Social comparison<br>6.3 Information about others' approval<br>9.1 Credible source

For peer review only

**Barriers/*facilitator* to target behaviour**	**Intervention components**	**Target construct (BCW)**	**Intervention function (BCW)**	**Behaviour Change Technique (using BCTv1)**
Concerns regarding the safety of physical activity [EO, QR]	• Reassure patients that gradual physical activity is safe (e.g. shouldn't cause too much shoe rubbing) and can be done when you do not have a DFU<br>• Address patients' individual physical activity concerns in the initiation phase	Psychological capability	Education	4.1 Instruction on how to perform the behaviour<br>5.1 Information about health consequences
Lack of belief in one's ability to engage in physical activity [LR] /Physical limitations (e.g. arthritis, breathlessness, foot discomfort/pain) [QR]/Bad weather [QR]<br><br>*Finding a suitable activity [QR]*	• Provide patient stories demonstrating how easy it was for other patients to engage in graded and regular physical activity, even though they are at high risk of developing DFUs or have health problems	Reflective motivation; Social opportunity	Persuasion; Modelling	6.2 Social comparison<br>6.3 Information about others' approval<br>9.1 Credible source
	• Provide a variety of examples of safe low-to-moderate physical activity, including activities that are non-weight bearing and can be done in bad weather	Psychological capability	Education	4.1 Instruction on how to perform the behaviour
Difficulties engaging in physical activity in the long-term [LR, QR]<br><br>*Social support [LR]; Provision of pedometers [LR, QR]; Integrating physical activity into your routine [QR]*	• Invite patients to set their own weekly physical activity goals<br>• Invite patients to self-monitor physical activity and provide personalised advice on how to modify goals based on self-monitoring<br>• Invite patients to make a physical activity action plan<br>• Invite patients to set easy-to-perform tasks and make them increasingly more difficult over time*	Reflective motivation; Psychological capability	Enablement; Training	1.1 Goal setting (behaviour)<br>1.4 Action planning<br>1.5 Review behaviour goal(s)<br>2.2 Feedback on behaviour<br>2.3 Self-monitoring of behaviour<br>8.7 Graded tasks*
	• Provide a free pedometer to those who would like one and encourage people to set daily step goals	Reflective motivation; Psychological capability; Physical opportunity	Enablement; Training; Environmental structuring	1.1 Goal setting (behaviour)<br>2.2 Feedback on behaviour<br>2.3 Self-monitoring of behaviour<br>12.5 Adding objects to the environment
	• Suggest that patients ask a friend/relative to exercise with them	Social opportunity	Enablement	3.1 Social support (unspecified)
	• Invite patients to regularly practice physical activity in the same context (e.g. after lunch)	Psychological capability; Automatic motivation	Training; Enablement	8.1 Behavioural practice/rehearsal<br>8.3 Habit formation

For peer review only

**Barriers/*facilitator* to target behaviour**	**Intervention components**	**Target construct (BCW)**	**Intervention function (BCW)**	**Behaviour Change Technique (using BCTv1)**
Forgetting to engage in physical activity [EO]<br><br>*Reminders [EO, LR]*	• Invite patients to set up email reminders to engage in physical activity and decide on the frequency of these reminders	Physical opportunity	Environmental structuring	7.1 Prompts/cues
**Target behaviour: Engaging in emotional management**				
Belief that emotional management will do little to delay getting a DFU or help with difficult emotions [EO; QR]	• Explain the necessity of emotional management for promoting engagement with the other foot care behaviours and provide evidence that they are effective for dealing with difficult emotions	Psychological capability; Reflective motivation	Education; Persuasion	5.1 Information about health consequences<br>5.6 Information about emotional consequences
	• Provide patient stories demonstrating how emotional management techniques helped other patients to take control of their DFUs	Psychological capability; Reflective motivation; Social opportunity	Education; Persuasion; Modelling	5.1 Information about health consequences<br>6.2 Social comparison<br>6.3 Information about others' approval<br>9.1 Credible source
Belief that emotional management is not relevant to them [QR]	• Provide emotional management as an optional part of the intervention<br>• Remind patients about emotional management at times of stress (e.g. if the foot health checklist highlights that they may have signs of getting a DFU)	N/A	N/A	N/A
Belief that the emotional management techniques do not fit with their preferable approach to emotional management [QR]	• Provide a range of techniques that may fit with a patients' preferred approach to emotional management (e.g. cognitive and behavioural techniques)	N/A	N/A	N/A
Lack of understanding regarding how to do the emotional management techniques [EO]	• Provide guidance on how to do the emotional management techniques	Psychological capability	Training	4.1 Instructions on how to perform the behaviour
	• Provide guided audio recordings of emotional management exercises	Physical opportunity	Environmental structuring	12.5 Adding objects to the environment
Lack of confidence in ability to practice emotional management techniques [EO]	• Provide patient stories demonstrating how easy it was for other patients to practice the emotional management techniques	Reflective motivation; Social opportunity	Persuasion; Modelling	6.2 Social comparison<br>6.3 Information about others' approval<br>9.1 Credible source

For peer review only

**Barriers/*facilitator* to target behaviour**	**Intervention components**	**Target construct (BCW)**	**Intervention function (BCW)**	**Behaviour Change Technique (using BCTv1)**
Difficulties engaging in emotional management in the long-term [EO]	• Invite patients to set their own emotional management practice goals	Reflective motivation	Enablement	1.1 Goal setting (behaviour)<br>1.4 Action planning<br>1.5 Review behaviour goal(s)
**Subsidiary behaviour: Engaging with the digital maintenance intervention**				
Low confidence in ability to use digital interventions [LR; QR]<br><br>*Technical support to use digital interventions [EO]*	• Health professionals introduce the digital maintenance intervention in the initiation phase and provide technical support as required<br>• Suggest that family and friends could assist the patient with digital intervention use, if appropriate	Physical capability; Psychological capability; Social opportunity	Training; Education; Enablement	3.2 Social support (practical)<br>4.1 Instructions on how to perform the behaviour
	• Health professionals will speak favourably about digital intervention use and outline its advantages<br>• Build patients' confidence in using the digital intervention by demonstrating how easy it is to use the intervention, and addressing any self-doubts*	Reflective motivation	Education; Persuasion	9.1 Credible source<br>15.1 Verbal persuasion about capability*

For peer review only - http://bmjopen.bmj.com/site/about/guidelines.xhtml

Greenwell, Sivyer et al. Intervention planning for the REDUCE maintenance intervention: a digital intervention to reduce re-ulceration risk among patients with a history of diabetic foot ulcers

COREQ checklist

No	Item	Guide questions/description		Location in manuscript
**Domain 1: Research team and reflexivity**				
Personal Characteristics				
1.	Interviewer/facilitator	Which author/s conducted the interview or focus group?	KG & KS	Qualitative interviews Methods – Page 11
2.	Credentials	What were the researcher's credentials? *E.g. PhD, MD*	KG – PhD, MSc, CPsychol.<br>KS – DPhil (graduand), MSc	-
3.	Occupation	What was their occupation at the time of the study?	KG – Health Psychologist & Research Fellow<br>KS – Research Fellow	-
4.	Gender	Was the researcher male or female?	Both female	-
5.	Experience and training	What experience or training did the researcher have?	Both interviewers received training on qualitative research methods in their MScs and have carried out several qualitative interview research projects.	-
Relationship with participants				
6.	Relationship established	Was a relationship established prior to study commencement?	Participants were not known to the interviewers prior to recruitment.	-
7.	Participant knowledge of the interviewer	What did the participants know about the researcher? e.*g. personal goals, reasons for doing the research*	Participants were told that the research findings were being used to develop an intervention to support them to look after their feet and improve their foot health.	-
8.	Interviewer characteristics	What characteristics were reported about the interviewer/facilitator? e.g. *Bias, assumptions, reasons and interests in the research topic*	Both interviewers are psychologists with a specialist interest in digital health interventions, which could be a potential source of bias. No other interviewer-related biases identified.	-

For peer review only

Greenwell, Sivyer et al. Intervention planning for the REDUCE maintenance intervention: a digital intervention to reduce re-ulceration risk among patients with a history of diabetic foot ulcers

**Domain 2: study design**				
Theoretical framework				
9.	Methodological orientation and Theory	What methodological orientation was stated to underpin the study? *e.g. grounded theory, discourse analysis, ethnography, phenomenology, content analysis*	Pragmatism & thematic analysis.	Qualitative interviews Methods – Page 11
Participant selection				
10.	Sampling	How were participants selected? *e.g. purposive, convenience, consecutive, snowball*	Purposive sampling.	Qualitative interviews Methods – Page 10
11.	Method of approach	How were participants approached? e.g. *face-to-face, telephone, mail, email*	Mail. We also advertised the research via social media channels and the website for a national charity, and used opportunistic recruitment by health professionals during consultations, however, no participants were recruited using these methods.	Qualitative interviews Methods – Page 10
12.	Sample size	How many participants were in the study?	20	Qualitative interviews Methods – Page 10
13.	Non-participation	How many people refused to participate or dropped out? Reasons?	Of those approached, 26% (66/250) expressed an interest to take part. Of the 21 eligible people we contacted for interview, only one did not go on to complete the interview due to competing time commitments. There were no withdrawals.	Qualitative interviews Methods – Page 10
Setting				
14.	Setting of data collection	Where was the data collected? e.g. *home, clinic, workplace*	At participants houses or the university	Qualitative interviews Methods – Page 11
15.	Presence of non-participants	Was anyone else present besides the	Relatives were occasionally present	-

For peer review only

Greenwell, Sivyer et al. Intervention planning for the REDUCE maintenance intervention: a digital intervention to reduce re-ulceration risk among patients with a history of diabetic foot ulcers

		participants and researchers?	during the interviews.	
16.	Description of sample	What are the important characteristics of the sample? *e.g. demographic data, date*	Demographic information can be found in Table 2. Interviews were carried out between April to May 2017.	Qualitative interviews Methods – Table 2 – Pages 11&12
Data collection				
17.	Interview guide	Were questions, prompts, guides provided by the authors? Was it pilot tested?	The interview schedule and prompt cards can be found in Appendix 1. They were pilot tested with two people with a history of diabetic foot ulcers.	Qualitative interviews Methods – Pages 10&11
18.	Repeat interviews	Were repeat interviews carried out? If yes, how many?	Only single interviews were carried out.	Qualitative interviews Methods – Page 10
19.	Audio/visual recording	Did the research use audio or visual recording to collect the data?	The audio from the interviews was digitally recorded.	Qualitative interviews Methods – Page 11
20.	Field notes	Were field notes made during and/or after the interview or focus group?	No	-
21.	Duration	What was the duration of the interviews or focus group?	Interviews lasted between 36 and 99 minutes	-
22.	Data saturation	Was data saturation discussed?	Participants were recruited until data saturation was reached.	-
23.	Transcripts returned	Were transcripts returned to participants for comment and/or correction?	No	-
**Domain 3: analysis and findings**				
Data analysis				
24.	Number of data coders	How many data coders coded the data?	Two (KS & KG)	Qualitative interviews Methods – Page 11
25.	Description of the coding tree	Did authors provide a description of the coding tree?	No	-
26.	Derivation of themes	Were themes identified in advance or derived from the	Major themes were identified in advance.	-

Greenwell, Sivyer et al. Intervention planning for the REDUCE maintenance intervention: a digital intervention to reduce re-ulceration risk among patients with a history of diabetic foot ulcers

		data?		
27.	Software	What software, if applicable, was used to manage the data?	QSR's NVivo 11 was used.	-
28.	Participant checking	Did participants provide feedback on the findings?	No	-
Reporting				
29.	Participant checking	Were participant quotations presented to illustrate the themes / findings? Was each quotation identified? e.g. *participant number*	Quotations were provided to illustrate each key finding. They are identified by a participant number and their gender is noted.	Qualitative interviews Results – Table 3 – Pages 12&13
30.	Data and findings consistent	Was there consistency between the data presented and the findings?	Yes	Qualitative interviews Results – Table 3 – Pages 12&13
31.	Clarity of major themes	Were major themes clearly presented in the findings?	Yes	Qualitative interviews Results – Table 3 – Pages 12&13
32.	Clarity of minor themes	Is there a description of diverse cases or discussion of minor themes?	Diverse cases are discussed.	Qualitative interviews Results – Pages 12-16

For peer review only
